# Extended Conjugation Attenuates the Quenching of Aggregation‐Induced Emitters by Photocyclization Pathways

**DOI:** 10.1002/anie.202202193

**Published:** 2022-04-13

**Authors:** Andrew T. Turley, Promeet K. Saha, Andrew Danos, Aisha N. Bismillah, Andrew P. Monkman, Dmitry S. Yufit, Basile F. E. Curchod, Marc K. Etherington, Paul R. McGonigal

**Affiliations:** ^1^ Department of Chemistry Durham University Lower Mountjoy, Stockton Road Durham DH1 3LE UK; ^2^ Department of Physics Durham University Lower Mountjoy, Stockton Road Durham DH1 3LE UK; ^3^ Department of Mathematics Physics and Electrical Engineering Northumbria University Ellison Place Newcastle upon Tyne NE1 8ST UK

**Keywords:** Aggregation-Induced Emission, Carbocycles, Fluorescence, Molecular Rotors, Photochemistry

## Abstract

Herein, we expose how the antagonistic relationship between solid‐state luminescence and photocyclization of oligoaryl alkene chromophores is modulated by the conjugation length of their alkenyl backbones. Heptaaryl cycloheptatriene molecular rotors exhibit aggregation‐induced emission characteristics. We show that their emission is turned off upon breaking the conjugation of the cycloheptatriene by epoxide formation. While this modification is deleterious to photoluminescence, it enables formation of extended polycyclic frameworks by Mallory reactions. We exploit this dichotomy (i) to manipulate emission properties in a controlled manner and (ii) as a synthetic tool to link together pairs of phenyl rings in a specific sequence. This method to alter the tendency of oligoaryl alkenes to undergo photocyclization can inform the design of solid‐state emitters that avoid this quenching mechanism, while also allowing selective cyclization in syntheses of polycyclic aromatic hydrocarbons.

## Graphical abstract

Reactions to shorten and lengthen the conjugation between oligoaryl alkene units of aggregation‐induced emitters toggle between rapid photocyclization and efficient photoluminescence properties. Trienes are resistant to photocyclization, giving them higher photoluminescence quantum yields, whereas nonconjugated aryl alkene units cyclize readily to form extended polycyclic frameworks.

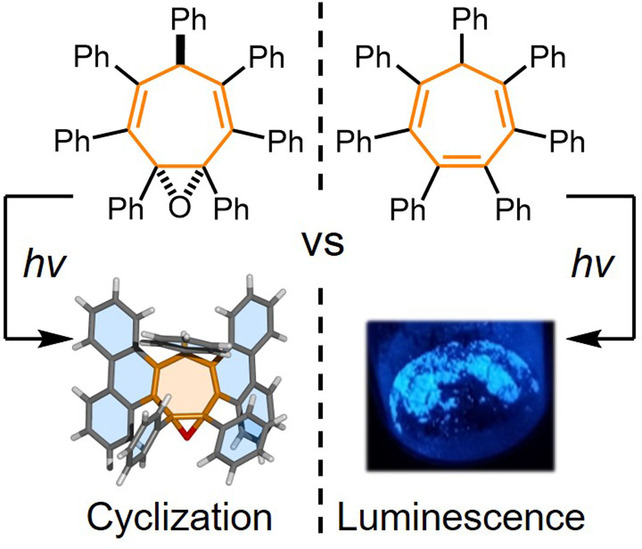

## Introduction

The quest to maximize photoluminescence efficiencies of organic luminogens has spurred progress from classical planar polycyclic aromatics, which are susceptible to aggregation‐caused quenching (ACQ),[^1^, ^2^, ^3^] toward non‐planar molecular rotor‐type frameworks that exhibit aggregation‐induced emission (AIE).[^4^, ^5^, ^6^, ^7^] The enhanced luminescence of AIE‐active materials in aggregated states is thought to arise from their restricted intramolecular motion (RIM),[^8^, ^9^] which minimizes the nonradiative decay of their excited states, thereby maximizing photoluminescent emission. However, this mechanistic description of AIE has been a topic of ongoing debate[^9^, ^10^, ^11^, ^12^, ^13^] that has progressed in parallel to the development of new AIE luminogens and their applications. Various possible quenching pathways that are “turned off” by RIM[^14^, ^15^] have been identified, such as through‐space aromatic dimerization,[^16^, ^17^, ^18^] *E*/*Z* isomerization^[19]^ and, more generally, restricted access to a conical intersection (RACI).[^20^, ^21^]

Oligoaryl alkenes, such as tetraphenylethylene (TPE), are a recurring structural motif in the pursuit of efficient emitters. These materials are regarded as the simplest, archetypal AIE‐active structures, possessing a molecular rotor‐type framework with rotatable phenyl rings.[^22^, ^23^] An added complication for these types of emissive materials is that their 1,2‐diphenyl ethylene (DPE) units are also known to undergo the Mallory reaction[^24^, ^25^] (a photochemical cyclization–elimination reaction that has been exploited synthetically to access polycyclic aromatic hydrocarbon structures).[^26^, ^27^] Indeed, the formation of Mallory reaction intermediates has been invoked as one of the quenching pathways for oligoaryl alkene AIE luminogens and has previously been studied through ultra‐fast spectroscopy,^[28]^ suggesting that photocyclization‐induced quenching (PIQ) is a dominant nonradiative loss pathway in the RACI mechanism.

Herein, we report synthetic modification of our previously reported^[16]^ AIE‐active molecular rotor compound *sym*‐heptaphenylcycloheptatriene (**Ph_7_C_7_H**) to study the effects of structural changes on its nonradiative energy loss pathways. Our strategy to modify the structure of **Ph_7_C_7_H** alters the extent of electronic conjugation present in the oligoaryl alkene rotor while limiting geometric changes to the system, allowing us to effectively decouple electronic and geometric effects. We demonstrate that by interrupting the conjugation of **Ph_7_C_7_H**, its characteristic dual‐state emission is quenched. Instead of luminescing, it undergoes facile photocyclization. Our investigations also lead to new understanding of the Mallory reaction in highly conjugated, oligoaryl alkene AIE luminogens. DPE units with extended π‐electron conjugation exhibit attenuated Mallory‐type reactivity, which correlates with suppressed PIQ and enhanced luminescence that is desirable for designing more efficient AIE materials. We exploit the facile photocyclization of “isolated” DPE units in a stepwise synthetic process that combines Mallory conditions with intermediate reactions to curtail or extend π‐conjugation. In doing so, we access fused polyaromatics that were previously inaccessible by direct Scholl oxidation or Mallory reactions alone.

## Results and Discussion

### Synthesis, Structure and Conformation

**Figure 1:**
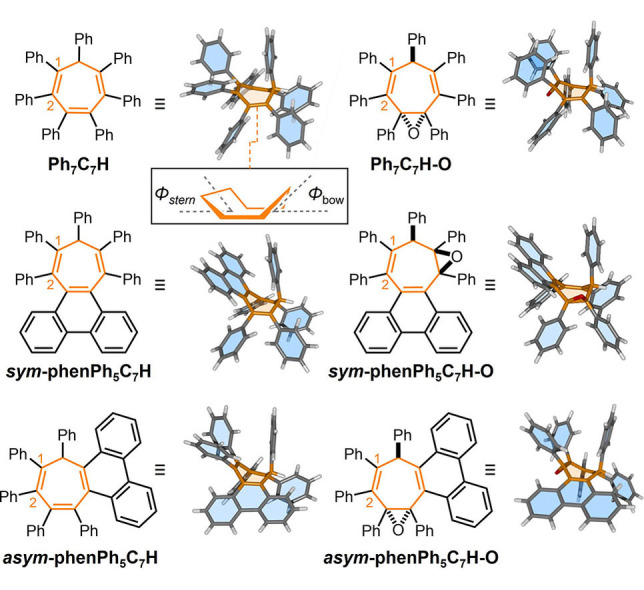
Structural formulas and X‐ray crystal structures of **Ph_7_C_7_H** derivatives. Inset: the boat conformation of a seven‐membered ring.

We initially synthesized modified derivatives of **Ph_7_C_7_H** (Figure 1) to further investigate its unusual dual‐state emission properties.^[16]^ See Scheme S1 of the Supporting Information for full synthetic details. Two types of structural modifications were introduced: (i) Formal ring fusion of two peripheral phenyl groups to form a phenanthrene unit or (ii) epoxidation of the central seven‐membered ring. Together, **Ph_7_C_7_H** and its symmetrical (***sym*** **‐phenPh_5_C_7_H**) and asymmetrical (***asym*** **‐phenPh_5_C_7_H**) 9,10‐phenanthryl analogs (Figure 1) make up a series of three triene rotors that differ by the introduction of a single C−C bond between two of the peripheral rings.

Subsequent epoxidation of each triene rotor expands the series to six compounds, giving rise (Figure 1) to **Ph_7_C_7_H‐O**, ***sym*** **‐phenPh_5_C_7_H‐O**, and ***asym*** **‐phenPh_5_C_7_H‐O**. Epoxidation occurs both stereoselectively and regioselectively in the presence of an excess of *meta*‐chloroperbenzoic acid (*m*CPBA). In each case, a singly epoxidized product was isolated. While epoxidation occurs at one end of the ***sym*** **‐phenPh_5_C_7_H** triene to give a diene system, the reaction occurs at the central double bonds of **Ph_7_C_7_H** and ***asym*** **‐phenPh_5_C_7_H**, splitting the trienes to give separated double bonds.

We gained insight into the conformations adopted by the rotor molecules by analyzing their X‐ray crystal structures (Figure 1).^[29]^ In the solid state, the cycloheptatriene rings exhibit similar, shallow boat‐like conformations across the series of six compounds. In each case, the phenyl group at the sp^3^‐C position occupies the bow of the boat conformation with a pseudo‐axial orientation. We have previously reported that this orientation influences the emissive excited state of **Ph_7_C_7_H** by formation of through‐space interactions between the phenyl rings at the bow and stern.^[16]^

In general, the proximity of the peripheral phenyl groups to one another disfavors conformations in which the rings are coplanar with the central double bonds, giving rise instead to perpendicular propeller‐like geometries. Nonetheless, enforcing coplanarity of two peripheral rings as part of a biphenylene substituent (forming a phenanthrene ring system) has little impact on the geometry (Table 1) of the central cycloheptatriene ring. For example, the interplane angles (Figure 1, inset) *Φ*_bow_=54.9° and *Φ*_stern_=35.3° that characterize the boat conformation of **Ph_7_C_7_H** vary (Table 1) by less than 10° for each of its five derivatives in Figure 1.

**Table 1:** Geometric parameters of the molecular rotors.

Rotor	*Φ*_bow_^[a]^ [°]	*Φ*_stern_^[a]^ [°]	BLA^[b]^ [pm]	*d*_1–2_^[c]^ [pm]	Δ*G* ^≠[d]^ (kJ mol^−1^)
**Ph_7_C_7_H**	54.9	35.3	12.2	135.1(3)	48.2
***sym*** **‐phenPh_5_C_7_H**	58.4	41.2	12.1	135.9(2)	44.5
***asym*** **‐phenPh_5_C_7_H**	50.9	43.9	13.5	135.5(5)	–
**Ph_7_C_7_H‐O**	49.0	33.1	–	134.1(2)	40.5
***sym*** **‐phenPh_5_C_7_H‐O**	53.2	42.2	14.8	135.4(2)	–
***asym‐*** **phenPh_5_C_7_H‐O**	46.1	35.2	–	133.8(2)	–

[a] Torsion angles and bond lengths were determined from X‐ray crystallographic data. [b] Measured by subtracting the average length of double bonds from the average length of single bonds that are part of the diene or triene system.^[30]^ [c] Estimated standard deviations in parentheses. [d] Measured by ^1^H VT NMR spectroscopy for rotation of the most hindered phenyl ring (Figures S25–S32).

Comparison of bond length alternation (BLA) parameters^[30]^ gives quantifiable insight into the influence of peripheral ring fusion and epoxidation on the conjugation of the central cycloheptatriene ring (Table 1). Higher BLA is indicative of reduced π‐electron delocalization of a conjugated system. Relative to **Ph_7_C_7_H**, the BLA of ***sym*** **‐phenPh_5_C_7_H** remains essentially unchanged, suggesting that the symmetrically placed phenanthrene ring has little impact on conjugation within the triene system. However, shortening this π‐system to a diene by epoxidation, i.e., ***sym*** **‐phenPh_5_C_7_H‐O**, causes a significant (≈20 %) increase in BLA, which demonstrates its reduced π‐electron delocalization. The asymmetrically substituted phenanthrene isomer, ***asym*** **‐phenPh_5_C_7_H**, is an intermediate case, exhibiting a slight increase (≈10 %) in BLA relative to **Ph_7_C_7_H**. On the other hand, epoxidation of either ***asym*** **‐phenPh_5_C_7_H** or **Ph_7_C_7_H** cleaves the triene system in two, producing at least one “isolated” double bond in each case, e.g., the C−C double bond between positions 1 and 2 of the seven‐membered ring (Figure 1). For both of these epoxides, the double bond length *d*_1–2_ (Table 1) has decreased by ≥1 pm relative to the triene precursor, which is consistent with its reduced conjugation.

The geometric similarities observed between the cycloheptatrienes in the solid state are reflected in the solution‐state dynamics for the 180° rotation of their most hindered phenyl rings. Variable‐temperature (VT) nuclear magnetic resonance (NMR) spectroscopic analysis (Figures S25–S32) reveals Gibbs energy barriers (Δ*G* ^≠^) to phenyl ring rotation at 298 K of 44.5 kJ mol^−1^ and 40.5 kJ mol^−1^ for ***sym*** **‐phenPh_5_C_7_H** and **Ph_7_C_7_H‐O**, respectively (Table 1). These values are similar to the Δ*G* ^≠^ measured for phenyl ring rotation in **Ph_7_C_7_H** of 48.2 kJ mol^−1^.^[16]^

In summary, neither epoxidation nor ring fusion causes large deviations in the overall geometries and conformational freedom of the seven‐membered rings or rotatable phenyl groups. However, epoxidation does cause significant changes to the lengths of the conjugated systems, modifying the electronic properties of the individual diaryl alkene units.

**Scheme 1:**
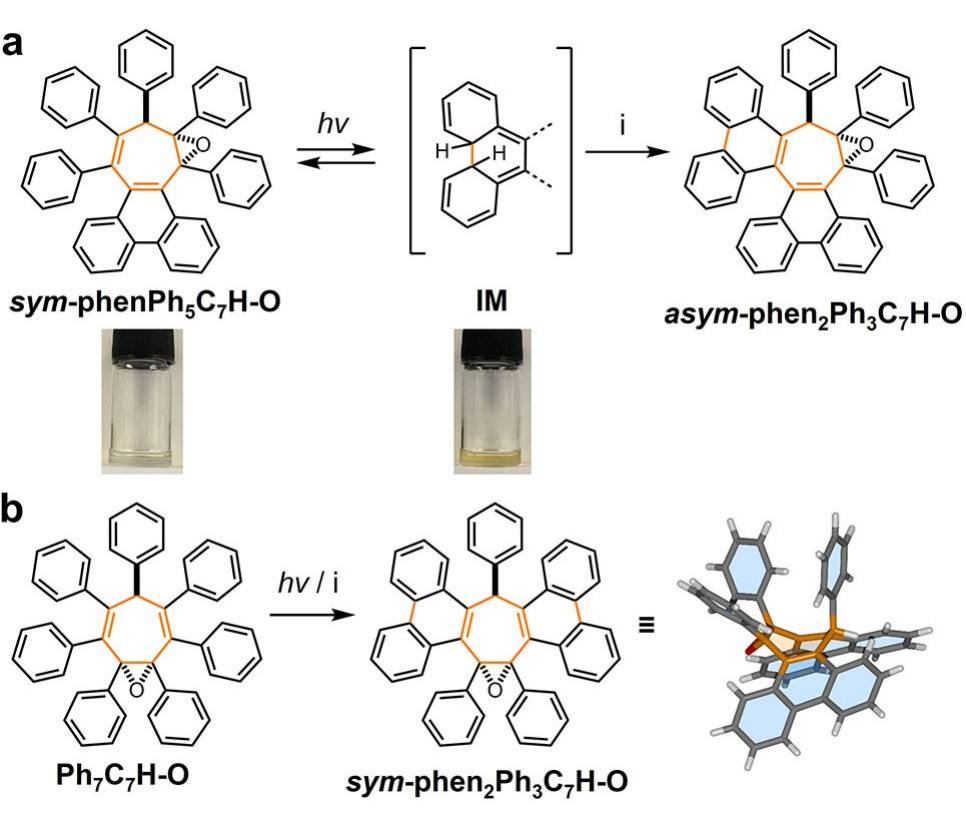
Photocyclizations of rotors containing “isolated” DPE units. Reagents and conditions: i) I_2_, propylene oxide, THF, *hv* (4.88 eV), 3 h. An X‐ray structure of ***sym*** **‐phen_2_Ph_3_C_7_H‐O** shows the presence of two new phenanthrene units.

We found that exposing a 1.5 mM toluene solution of ***sym*** **‐phenPh_5_C_7_H‐O** to UV irradiation causes a color change from colorless to yellow (Scheme 1a), which is reversed in ambient light over time. Similar reversible yellowing of TPE films has previously been attributed to formation of a photocyclized species.^[28]^ No such color change (or reactivity, vide infra) was observed for ***sym*** **‐phenPh_5_C_7_H**, which suggests that the broken conjugation of ***sym*** **‐phenPh_5_C_7_H‐O** significantly alters its photophysical behavior. The proposed intermediate species **IM** formed under irradiation was subsequently oxidized (vide infra) to ***asym*** **‐phen_2_Ph_3_C_7_H‐O** under Mallory reaction conditions, confirming the photocyclization reactivity. Similarly, a Mallory reaction of **Ph_7_C_7_H‐O** gives rise to ***sym*** **‐phen_2_Ph_3_C_7_H‐O**, which was confirmed by two‐dimensional NMR spectroscopy and X‐ray crystallography (Scheme 1b). To elucidate the origins of this enhanced photocyclization reactivity in the compounds with truncated conjugation, we conducted deeper photophysical analyses of the series of six rotors shown in Figure 1.

### Luminescence of the Triene Rotors

**Figure 2:**
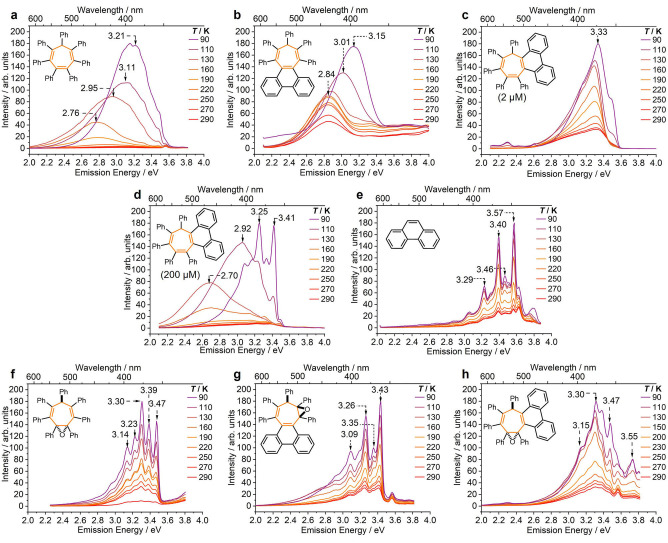
VT steady‐state photoluminescence spectra of 2‐MeTHF solutions of a) **Ph_7_C_7_H**, excitation energy *E*_ex_=3.94 eV, concentration *c*=2 μM, b) ***sym*** **‐phenPh_5_C_7_H**, *E*_ex_=4.13 eV, *c*=2 μM, c) ***asym*** **‐phenPh_5_C_7_H**, *E*_ex_=3.94 eV, *c*=2 μM, d) ***asym*** **‐phenPh_5_C_7_H**, *E*_ex_=3.94 eV, *c*=200 μM, e) phenanthrene, *E*_ex_=4.13 eV, *c*=2 μM, f) **Ph_7_C_7_H‐O**, *E*_ex_=4.35 eV, *c*=2 μM, g) ***sym*** **‐phenPh_5_C_7_H‐O**, *E*_ex_=3.94 eV, *c*=2 μM, and h) ***asym*** **‐phenPh_5_C_7_H‐O**, *E*_ex_=3.95 eV, *c*=2 μM.

We first confirmed that the triene molecular rotors are AIE‐active by preparing 1 % w/w dispersions in ZEONEX—an optically clear cyclic olefin polymer matrix. Photoluminescene quantum yield (*Φ*_film_) measurements carried out on the ZEONEX films show that all three fully conjugated rotors emit in the solid state with similar efficiencies (Table 2) of 1.7–6.7 %. We gained further insights into the influence of biphenylene substitution on the optical properties relative to **Ph_7_C_7_H** by acquiring (Figure 2) steady‐state emission spectra^[31]^ using dilute (2 μM) 2‐methyl tetrahydrofuran (2‐MeTHF) solutions of each rotor. The spectra were recorded at a series of temperatures (290 K to 90 K) to alter rates of nonradiative decay (*k*_nr_) through RIM and RACI in a controlled manner.

**Table 2:** Photophysical properties of the molecular rotors and phenanthrene.

Rotor	*E*_e*x*_^[a]^ [eV]	*E*_max_ [eV]			τ [ns]^[b,c]^	Δ*I*_290–90_^[b,d]^		*Φ*_film_^[e,f]^ [%]
		160 K^[b]^	90 K^[b]^	Film^[e]^	290 K	Sol^[b]^	Film^[e]^	
**Ph_7_C_7_H**	3.95	2.76	3.21	3.02	6.5	52.1	9.91	6.7
***sym*** **‐phenPh_5_C_7_H**	4.13	2.84	3.15	3.01	20.4	3.75	2.13	1.7
***asym*** **‐phenPh_5_C_7_H**	3.94	3.33	3.33	3.07	8.2	6.24	2.63	4.0
**Ph_7_C_7_H‐O** ^[e]^	4.35	3.30	3.30	3.47	12.6^[g]^	4.82	1.19	≤0.1
***sym*** **‐phenPh_5_C_7_H‐O** ^[e]^	3.94	3.43	3.43	3.34	11.9^[g]^	5.76	0.69	≤0.1
***asym‐*** **phenPh_5_C_7_H‐O** ^[e]^	3.94	3.30	3.30	3.47	9.0^[g]^	5.47	2.62	3.3
***sym‐*** **phen_2_Ph_3_C_7_H‐O**	4.00	3.72	3.76	3.43	–	1.67	1.50	6.4
***asym‐*** **phen_2_Ph_3_C_7_H‐O**	4.00	3.43	3.43	3.43	–	4.50	2.65	9.8
***sym‐*** **phen_3_PhC_7_H**	3.95	3.40	3.40	3.22	8.4	3.97	–	4.5
phenanthrene	4.13	3.56	3.56	3.06	15.0	5.13	0.56	–

[a] *E*_ex_ were chosen to match peaks in absorption spectra (Figure S48). [b] 2 μM solution in 2‐MeTHF. [c] See Table S4 for full lifetime data. [d] Difference in intensity between the emission peaks at 290 K and 90 K. [e] 1 % w/w film in ZEONEX. [f] Measured by integrating sphere under ambient conditions. [g] The τ values reported for the epoxide series are the apparent lifetimes of the weak emission observed upon excitation of a sample of the pure epoxide starting material, some of which may photocyclize during the measurement.

We measured (Table 2) the ratios between emission intensities at 290 K and 90 K, Δ*I*_290–90_. The emission intensities, *I*, of the three triene rotors increase at lower temperatures as *k*_nr_ is decreased. Both phenanthrenyl derivatives have much smaller Δ*I*_290–90_ than **Ph_7_C_7_H**. This observation is consistent with them having fewer intramolecular vibrational degrees of freedom, which renders them less susceptible to increased nonradiative decay at higher temperatures.

In common with **Ph_7_C_7_H** (Figure 2a), ***sym*** **‐phenPh_5_C_7_H** shows (Figure 2b) a gradual hypsochromic shift in the energy of the emission maximum (*E*_max_) from 2.84 eV to 3.15 eV as the temperature decreases (Table 2). By analogy to our previous investigation,^[16]^ we attribute this shift to two‐state emission that emerges from accessing a relaxed dimer state, whereby a face‐to‐face interaction develops between the phenanthrene moiety and the phenyl group at the bow of the seven‐membered ring after photoexcitation. The compound‐specific relationships between the *E*_max_ and temperature are plotted in Figure S52.

There is no indication of this dual emission in the analogous spectra (Figure 2c) of ***asym*** **‐PhenPh_5_C_7_H**. However, we do observe (Figure 2d) a bathochromic shift from 3.33 eV to 2.70 eV at 130 K using a higher solution concentration of 200 μM, suggesting the formation of intermolecular dimers rather than the intramolecular aromatic interactions. This behavior mirrors the dimerization of molecular phenanthrene.^[32]^ Presumably, the phenanthrene moiety of ***asym*** **‐phenPh_5_C_7_H** is sufficiently exposed and unhindered that it is available to undergo intermolecular dimerization in the ground state driven by solvophobic forces. Indeed, the vibronic structure of the emission from ***asym*** **‐PhenPh_5_C_7_H** at 200 μM matches closely with the emission from phenanthrene (Figure 2e). We note that no concentration‐dependent emission changes were observed in the spectra of other rotors in the series (Figure S54).

Overall, the photophysical characteristics of both ***sym***‐ and ***asym*** **‐phenPh_5_C_7_H** broadly resemble **Ph_7_C_7_H**. All three are AIE‐active rotors that give photoluminescence quantum yields of 1.7–6.7 % in the solid state (i.e., dispersed in ZEONEX films). The relative position of the biphenylene unit of the molecular rotor tunes the propensity for face‐to‐face interactions of their aromatic rings, which can occur intramolecularly in the excited state or intermolecularly in the ground state. Yet, there is no evidence in their optical spectra to suggest that these fully conjugated compounds are prone to photocyclization.

### Optical Properties of the Epoxide Rotors

Interrupting the conjugation of the triene systems by epoxidation causes significant changes in their emission properties. The broad fluorescence peaks observed for the trienes are replaced (Figures 2f–h) by structured emission at high energies (3.09–3.55 eV). The emission intensities are very weak in both the solution and solid states regardless of temperature (Figure S55), giving low *Φ*_film_ values (Table 2). ZEONEX films of **Ph_7_C_7_H‐O** and ***sym*** **‐phenPh_5_C_7_H‐O** both give quantum yields below the threshold for reliable measurement (≤0.1 %).^[33]^ Therefore, the reduced conjugation of the epoxides is associated with substantially diminished luminescence.

We ascribe these observations to PIQ occurring in both the solution state and amorphous ZEONEX films. Rather than undergoing photoluminescence, the excited rotors are prone to form a C−C bond to give **IM** structures (Scheme 1a), which enables the aforementioned Mallory reactivity. This enhanced PIQ pathway is responsible for the absence of the broad emission peak observed for the trienes.

**Figure 3:**
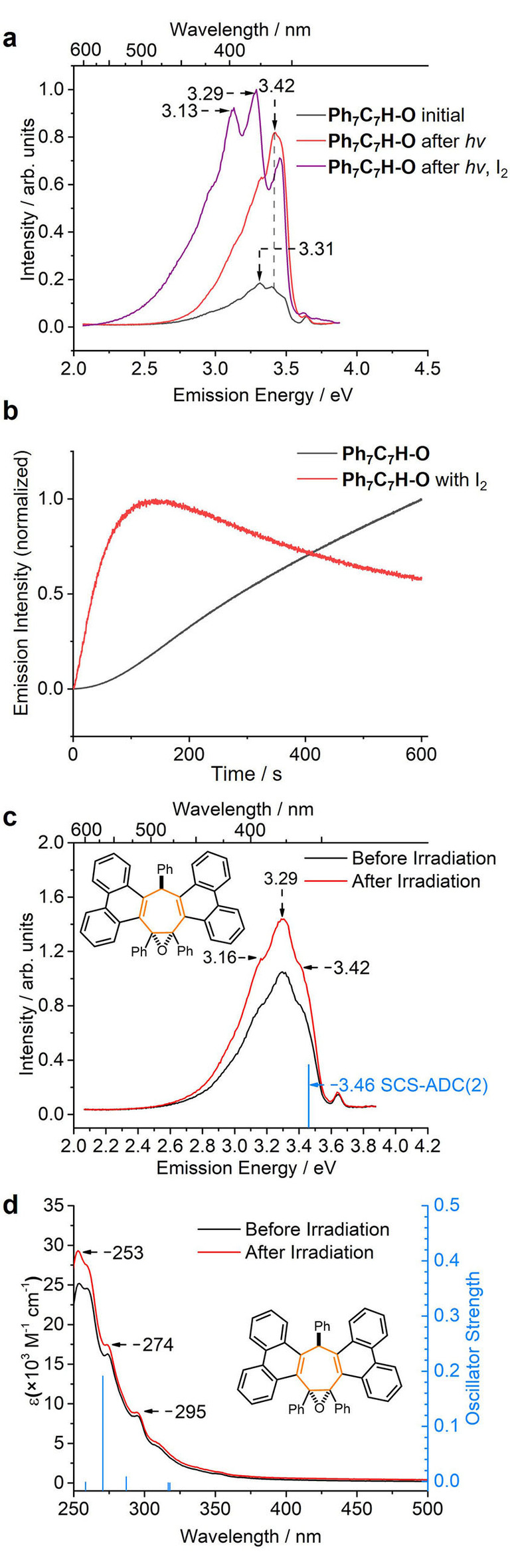
Steady‐state photoluminescence spectra of 2‐MeTHF solutions of **Ph_7_C_7_H‐O**, (*E*_ex_=4.35 eV, *c*=2 μM) a) before and after irradiation with 4.0 eV light for 10 min with and without I_2_; b) the change in the emission intensity at 3.30 eV over time, with and without I_2_; c) emission (2‐MeTHF, *c*=2 μM) and d) absorption (MeCN, *c*=20 μM) spectra of ***sym*** **‐phen_2_Ph_3_C_7_H‐O** before and after irradiation with 4.0 eV light for 10 min. Theoretical emission (vertical transition from the S_1_ geometry) and absorption (five lowest vertical transitions from the S_0_ geometry) transitions shown in panels c and d were obtained at the SCS‐ADC(2)/TZVP//SCS‐ADC(2)/SVP and SCS‐ADC(2)/TZVP//SCS‐MP2/SVP levels of theory, respectively.

A further experimental indication of this photocyclization occurring in solution came when sequentially repeated steady‐state photoluminescence measurements of a 2‐MeTHF solution of **Ph_7_C_7_H‐O** resulted in increased emission intensity. We compared (Figure 3a) the emission spectrum before and after irradiating a sample with 4.0 eV light for 5 min. Irradiation causes a fourfold increase in emission intensity, consistent with a photochemical reaction taking place to build up a higher concentration of a more emissive compound. An experiment monitoring the emission intensity at 3.30 eV over time as the sample is irradiated (Figure 3b) shows the buildup of the new species during continued irradiation. The emission intensity decreases when the irradiation is stopped, consistent with the reversible color change described above (Scheme 1).

Performing a similar series of experiments for the full series of six rotors (Figure S58), we found that the three epoxide rotors exhibit the same behavior, whereas the trienes require prolonged irradiation times of >1 h before changes in their emission profiles are observed.

These observations are all consistent with PIQ. To confidently rule out the possibility of the structured emission stemming from solvatochromic, or intermolecular interactions, we also acquired absorption (Figure S48) and emission spectra (Figure S56) using (i) solvents of different polarities and (ii) amorphous film samples prepared as 1 % w/w dispersions in ZEONEX (Figure S55). We observed that, in general, the spectral features are independent of solvent polarity and temperature.

In light of the proposed photocyclization pathway (Scheme 1), we tested whether atmospheric O_2_ was acting as an oxidant to complete the irreversible formation of ***sym‐*** **phen_2_Ph_3_C_7_H‐O** upon irradiating a 2‐MeTHF solution of **Ph_7_C_7_H‐O**. A solution sample of **Ph_7_C_7_H‐O** was deaerated by performing freeze‐pump‐thaw cycles and backfilling with N_2_, before irradiating with UV light. The same emission profile was observed under these conditions or using an aerated sample (Figure S56). It is, therefore, unlikely that significant oxidation of the planarized **IM** compounds is occurring under these conditions. Instead, the weak, structured emission spectra recorded in Figures 2f–h should be attributed to emission from the **IM** structures themselves. Trace amounts of **IM** compounds formed during the measurement may become photoexcited and subsequently luminesce.

### Epoxide Rotors Undergo Facile Mallory Reaction

We further probed the susceptibility of the epoxides to undergo photocyclization by purposefully applying Mallory reaction conditions (Scheme 1). An excess of the oxidants I_2_ and propylene oxide were added to a 15 mM solution of **Ph_7_C_7_H‐O** in tetrahydrofuran (THF) to trap transient **IM** compounds through the irreversible formation of a new phenanthrene ring system (Scheme 1b). The double photocyclization product, ***sym*** **‐phen_2_Ph_3_C_7_H‐O**, was isolated in quantitative yield after 3 h of irradiation with 4.88 eV light. Two new phenanthrene moieties form from the two “isolated” (i.e., non‐conjugated) DPE units of **Ph_7_C_7_H‐O**. Adding further equivalents of I_2_ and extending the irradiation time does not promote further photocyclization of the remaining phenyl groups.

The same reaction conditions were also applied to ***sym*** **‐phenPh_5_C_7_H‐O**, which has a diene system in its central seven‐membered ring. Photooxidation of its sole DPE group produced (Scheme 1a) ***asym*** **‐phen_2_Ph_3_C_7_H‐O** in 98 % yield.

**Scheme 2:**
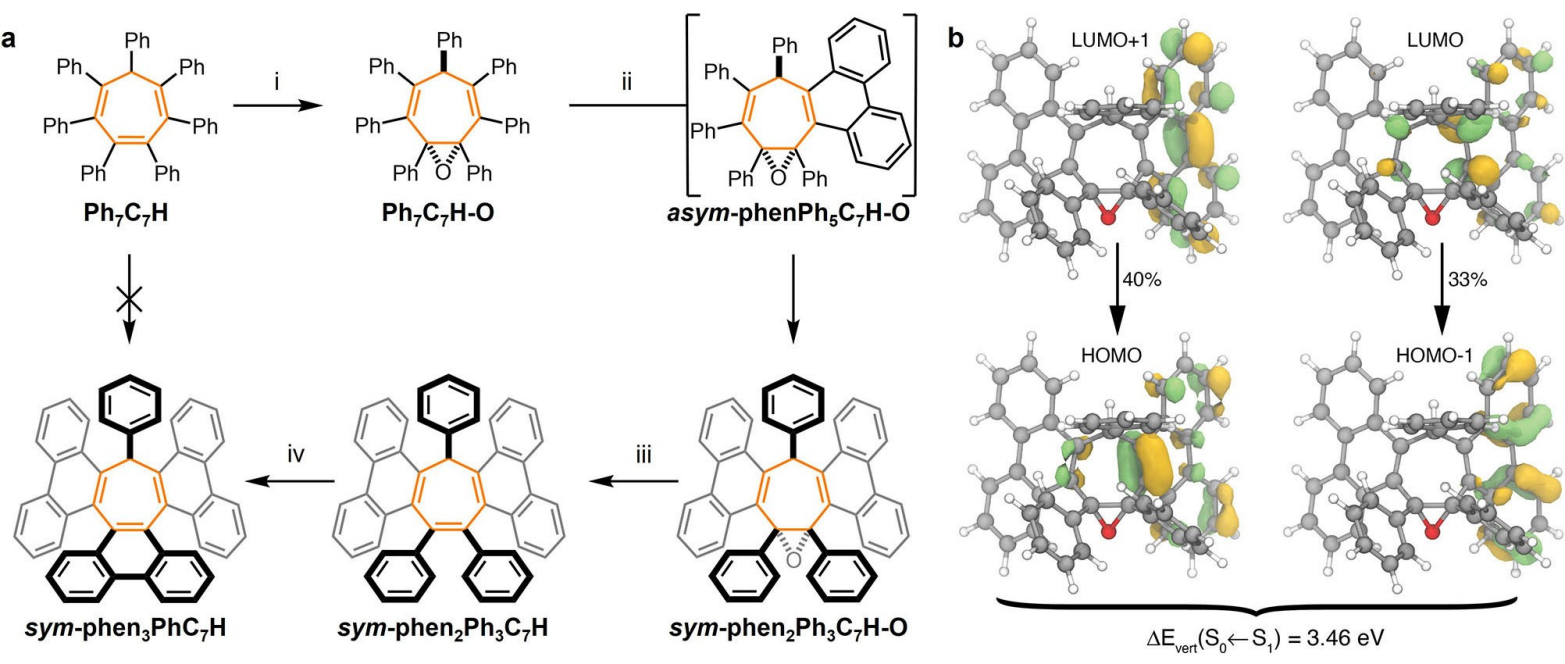
a) Sequential Mallory reactions by isolating and revealing DPE groups. Reagents and conditions: i) *m*CPBA, CHCl_3_, 0 °C to 55 °C, 24 h, 70 %; ii) I_2_, THF, propylene oxide, *hν* (4.88 eV), 3 h, >99 %; iii) LiAlH_4_, THF, 70 °C, 72 h, 47 %; iv) I_2_, THF, propylene oxide, *hν* (4.88 eV), 15 min, 81 %. b) Calculated MOs of ***sym*** **‐phen_2_Ph_5_C_7_H‐O**, SCS‐ADC(2)/TZVP.

These Mallory reaction conditions were also applied successfully to the third epoxide rotor, ***asym*** **‐phenPh_5_C_7_H‐O**, which can be considered as a monocyclized intermediate species that forms (Scheme 2a) during the irreversible transformation of **Ph_7_C_7_H‐O** to ***sym*** **‐phen_2_Ph_3_C_7_H‐O**. As expected, the photocyclization also gave ***sym*** **‐phen_2_Ph_3_C_7_H‐O**, doing so in quantitative yield.

Conversely, none of the triene rotors shown in Figure 1 underwent photooxidation under these reaction conditions. Even upon irradiating for extended periods, ^1^H NMR spectroscopic analyses of the crude reaction mixtures indicated that no observable reactions occur.

Therefore, these synthetic results are in agreement with our spectroscopic experiments. Photocyclization of isolated DPE units occurs rapidly. The diene ***sym*** **‐phenPh_5_C_7_H‐O** shows similar reactivity and photophysical properties to the isolated DPE compounds. But the extended conjugation of the three triene rotors improves their resistance to photocyclization, both under Mallory reaction conditions and in the context of a quenching pathway for photoluminescence.

### Exhaustive Photocyclization

Once formed, the doubly photocyclized product ***sym*** **‐phen_2_Ph_3_C_7_H‐O** displays (Figure 3a) an emission spectrum that is distinct from the spectrum of its **IM** precursors. The time‐dependent increase in emission intensity at 3.30 eV observed previously during irradiation of **Ph_7_C_7_H‐O** (Figure 3b) is also changed as the **IM** compounds are consumed by onward reaction with I_2_ to form ***sym*** **‐phen_2_Ph_3_C_7_H‐O**.

We tested the emission (Figure 3c) and absorption (Figure 3d) spectra of solutions of isolated ***sym*** **‐phen_2_Ph_3_C_7_H‐O** before and after irradiation at 4.88 eV to probe whether further photocyclization can occur. Only small differences in intensity were observed, which likely arise because of minor changes in temperature and concentration following irradiation. In keeping with the outcome of the Mallory reactions, it does not appear that the remaining phenyl groups are prone to undergo further photocyclization. The first five vertical transitions of ***sym*** **‐phen_2_Ph_3_C_7_H‐O** calculated with SCS‐ADC(2) (see the Supporting Information for computational details) are in good agreement with the experimental absorption spectrum (Figure 3d). These transitions, which are within the range of the photoexcitation energy used experimentally, involve orbitals on the phenanthrenes or the central seven‐membered ring, validating the observation that no further cyclization from the phenyl rings is to be expected. The vertical emission energy from the optimized S_1_ geometry obtained at the SCS‐ADC(2)/TZVP//SCS‐ADC(2)/SVP is calculated at 3.46 eV (Figure 3c), closely matching the high‐energy tail of the experimental spectrum. The orbitals involved in the emission (Scheme 2b) are located on the phenanthrene and central rings and do not extend to the remaining phenyl groups. The shape of the emission band of ***sym*** **‐phen_2_Ph_3_C_7_H‐O** was further analyzed by including non‐Condon effects using the nuclear ensemble approach combined with linear‐response time‐dependent density functional theory (Figure S63).

### Control of Conjugation Length Enables Synthesis

Based on the differing reactivities of the trienes and the epoxides, we hypothesized that by synthetically manipulating the conjugation lengths in our oligoaryl alkene systems we could enable otherwise inaccessible reactivity. Accordingly, we targeted (Scheme 2a) the *meso* double helicene ***sym*** **‐phen_3_PhC_7_H**. Attempts to prepare this compound directly from **Ph_7_C_7_H** using a range of classical intramolecular cyclodehydrogenation reaction conditions^[34]^ have been unsuccessful.^[35]^

After breaking the triene conjugation of **Ph_7_C_7_H** by epoxidation, the Mallory reaction of **Ph_7_C_7_H‐O** to give ***sym*** **‐phen_2_Ph_3_C_7_H‐O** proceeds smoothly on gram scale. The reaction was performed using I_2_ in THF solution while irradiating with 4.88 eV UV light. Having confirmed that no further photocyclization occurs in ***sym*** **‐phen_2_Ph_3_C_7_H‐O**, the epoxide unit was then reverted to an olefin to reestablish a DPE unit in the structure. We treated ***sym*** **‐phen_2_Ph_3_C_7_H‐O** with a 1 M solution of LiAlH_4_ in THF at 70 °C, which yielded ***sym*** **‐phen_2_Ph_3_C_7_H**.

Pleasingly, upon reformation of this DPE unit, further cyclization to give ***sym*** **‐phen_3_PhC_7_H** occurs readily—a CDCl_3_ solution sample of ***sym*** **‐phen_2_Ph_3_C_7_H** cyclized when left under ambient atmosphere and light for 7 d. The process is accelerated by applying our standard Mallory reaction conditions, which gives rise to the double helicene product in 81 % yield after 15 min. By analogy to the increased BLA of ***asym*** **‐PhenPh_5_C_7_H** (Table 1), we attribute the ease of this final photocyclization to reduced electron delocalization in the π‐system of the central seven‐membered ring, which is caused by the two phenanthrene units at either end of the conjugated system.^[36]^

Overall, the efficiency of the central ring epoxidation and subsequent “deprotection” to return the olefin functionality provides an attractive tactic to manipulate photocyclizations of DPE‐containing molecules. The method complements existing approaches for the synthesis of polycyclic aromatic hydrocarbons.^[34]^ Conceptually, the steps involved are: (1) Disrupting conjugation to form an “isolated” DPE unit, (2) carrying out a Mallory reaction, then (3) reestablishing the conjugated system by reversing step 1.

### Minimizing PIQ

Comparing the full series of DPE‐containing rotor compounds (Table 2), we suggest two strategies for molecular design to minimize PIQ and optimize their solid‐state emission. The propensity of DPE units to undergo photocyclization can be reduced by incorporating them as part of an extended conjugated system. **Ph_7_C_7_H**, ***sym***‐ and ***asym*** **‐PhenPh_5_C_7_H** have not shown spectroscopic evidence of rapid PIQ or synthetic evidence of Mallory reaction. They give *Φ*_film_ values of 1.7–6.7 %, which are higher than those measured for their epoxide counterparts with reduced conjugation. Alternatively, rotor compounds that are prone to PIQ can be treated with Mallory reaction conditions to purposefully exhaust the available photocyclization pathways. Our experiments have shown that ***sym*** **‐phen_2_Ph_5_C_7_H‐O**, ***asym*** **‐phen_2_Ph_5_C_7_H‐O** and ***sym*** **‐phen_3_PhC_7_H** are all resistant to photocyclization under Mallory conditions. Their three‐dimensional structures and remaining rotatable phenyl groups presumably contribute to them retaining their AIE properties. In the solid state, they give *Φ*_film_ values of 4.5–9.8 %, which exceed their precursors. Depending on the structure of the AIE compound and its intended use, one of these two strategies may be more appropriate than the other. Low molecular weight AIE luminogens may be required for use in some applications, which may place limitations on the length of the oligoalkenyl backbone that can be used.

## Conclusion

In summary, by making minor structural changes to an AIE‐active molecular rotor, **Ph_7_C_7_H**, we have exerted influence over the prevalence of PIQ and the formation of intramolecular phenyl‐ring dimer excited states. PIQ is disfavored in these rotor compounds when their individual DPE units form part of a larger conjugated system. A parallel can be drawn to the related Mallory photocyclization reaction; compounds that are prone to PIQ undergo facile reaction to form annulated derivatives under Mallory conditions. We have leveraged this property by reversibly disrupting conjugation through epoxidation, which gives access to fused polyaromatics that were previously inaccessible through conventional aryl‐aryl coupling reactions. These methods to manipulate the photophysical properties of oligoaryl alkene units can be exploited in the design of more efficient AIE materials or to improve photochemical syntheses of polycyclic aromatic hydrocarbons.

## Conflict of interest

The authors declare no conflict of interest.

1

## Supporting information

As a service to our authors and readers, this journal provides supporting information supplied by the authors. Such materials are peer reviewed and may be re‐organized for online delivery, but are not copy‐edited or typeset. Technical support issues arising from supporting information (other than missing files) should be addressed to the authors.

Supporting Information

Supporting Information

Supporting Information

Supporting Information

Supporting Information

Supporting Information

Supporting Information

Supporting Information

## Data Availability

The data that support the findings of this study are available in the Supporting Information of this article.
