# Supplementary material for: Extended Conjugation Attenuates the Quenching of Aggregation‐Induced Emitters by Photocyclization Pathways
Source: Angew Chem Int Ed Engl. 2022 Apr 13;61(24):e202202193. doi: 10.1002/anie.202202193 (PMC9325432; doi:10.1002/anie.202202193)
Supplement: Supplementary file 3 — Supporting Information [file ANIE-61-0-s001.pdf]

Angewandte Chemie
Eine Zeitschrift der Gesellschaft Deutscher Chemiker

Supporting Information

Extended Conjugation Attenuates the Quenching of Aggregation-Induced Emitters by Photocyclization Pathways

*A. T. Turley, P. K. Saha, A. Danos, A. N. Bismillah, A. P. Monkman, D. S. Yufit, B. F. E. Curchod, M. K. Etherington, P. R. McGonigal**

Table of Contents

1. General Methods S2
2. Synthetic Procedures S4
3. ^{1}H and ^{13}C NMR Spectroscopic Characterization of Synthesized Compounds S18
    3.1. Variable-Temperature (VT) NMR Spectroscopy S44
4. X-Ray Crystallographic Analysis S50
    4.1. **$Ph_3C_3{\cdot}HCl_2$** S51
    4.2. **Ph_7C_7H-O** S52
    4.3. ***sym*-phenPh_5C_7H** S54
    4.4. ***asym*-phenPh_5C_7H** S56
    4.5. ***sym*-phenPh_5C_7H-O** S58
    4.6. ***asym*-phenPh_5C_7H-O** S60
    4.7. ***sym*-$phen_2Ph_3C_7H$-O** S62
5. UV-Vis Absorption Spectroscopy S64
6. VT Fluorescence Spectroscopy S68
    6.1. Temperature-Dependent Solid-State Photoluminescence S73
7. Fluorescence Solvatochromism S75
8. Phenanthrene S77
9. Photooxidation S79
10. Time-Correlated Single Photon Counting (TCSPC) S81
11. New Photocyclized Rotors S82
12. Hyperchromism S83
13. Computational Details S84
14. References S85

Supporting Information

1. General Methods

Materials: All reagents were purchased from commercial suppliers (Sigma-Aldrich, Acros Organics, or Alfa Aesar) and used without further purification. *meta*-Chloroperbenzoic acid (*m*CPBA) was purchased and used as a mixture of >77% purity, where the remainder is *m*-chlorobenzoic acid and water.

Instrumentation and Analytical Techniques: Microwave reactions were performed using an automated Biotage reactor, Robot-Sixty, model: Initiator EXP EU, 355301, 11327-36W. Analytical thin-layer chromatography (TLC) was performed on neutral aluminium sheet silica gel plates and visualised under UV irradiation (254 nm). Nuclear magnetic resonance (NMR) spectra were recorded using a Bruker Advance (III)-400 (^{1}H 400.130 MHz and ^{13}C 100.613 MHz), Varian Inova-500 (^{1}H 500.130 MHz and ^{13}C 125.758 MHz), Varian VNMRS-600 (^{1}H 600.130 MHz and ^{13}C 150.903 MHz) or a Varian VNMRS-700 (^{1}H 700.130 MHz and ^{13}C 176.048 MHz) spectrometer, at a constant temperature of 298 K unless otherwise stated. For Variable Temperature (VT) measurements, operating temperatures were calibrated using an internal calibration solution of MeOH and glycerol. Chemical shifts (δ) are reported in parts per million (ppm) relative to the signals corresponding to residual non-deuterated solvents [$CDCl_3$: δ = 7.26 or 77.16. CD_2Cl_2: δ = 5.32 or 54.00. $(CD_3)_2CO$: δ = 2.05 or 29.84. CD_3CN: δ = 1.94 or 118.26]. Coupling constants (J) are reported in Hertz (Hz). ^{13}C NMR Experiments were proton decoupled. Assignment of ^{1}H and ^{13}C NMR signals were accomplished by two-dimensional (2D) NMR spectroscopy (COSY, NOESY, HSQC, HMBC). NMR spectra were processed using MestReNova version 11. Data are reported as follows: chemical shift; multiplicity; coupling constants; integral and assignment. Low-resolution ASAP-MS were performed using a Waters Xevo QTOF equipped with an Atmospheric Solids Analysis Probe (ASAP). High-resolution electrospray (HRESI) and ASAP (HRASAP) mass spectra were measured using a Waters LCT Premier XE high resolution, accurate mass UPLC ES MS (also with ASAP ion

source). Melting points were recorded using a Gallenkanp (Sanyo) apparatus and are uncorrected. The X-ray single crystal data have been collected at 120.0(2)K using λMoKα radiation (λCuKα for compound ***asym*-phenPh$_5$C$_7$H**; λ =0.71073 and 1.54178Å respectively) on a Bruker D8Venture (Photon100 CMOS detector, IμS-microsource (Incoatec Inc), focusing mirrors, 3-circle goniometer) diffractometer equipped with a Cryostream (Oxford Cryosystems) open-flow nitrogen cryostat. Data were integrated by SAINT (Bruker AXS) and scaled and corrected for absorption by SADABS (Bruker AXS). All structures were solved by direct method and refined by full-matrix least squares on F^2 for all data using Olex2[1] and SHELXTL[2] software. All non-disordered non-hydrogen atoms were refined in anisotropic approximation, hydrogen atoms in structure ***sym*-phenPh$_5$C$_7$H** were refined isotropically, in all other structures hydrogen atoms were placed in the calculated positions and refined in riding mode. Crystal data and parameters of refinement are listed in Sections 4.3–4.7. Crystallographic data for the structure have been deposited with the Cambridge Crystallographic Data Centre as supplementary publication CCDC-2144322-2414328. Steady-state photoluminescence of films and solutions were measured using Jobin Yvon Fluoromax and Fluorlog with machine-specific calibration curves. The low temperature and temperature-dependent photoluminescence spectra were measured using a Janis Research Co. Inc. nitrogen filled cryostat. Thus, at low temperature, films were in a nitrogen atmosphere and solutions in a sealed long-neck cuvette. Photocyclization reactions were performed with a Rio 36 W UV lamp fitted with 4 × 9W 254 nm bulbs.

2. Synthetic Procedures

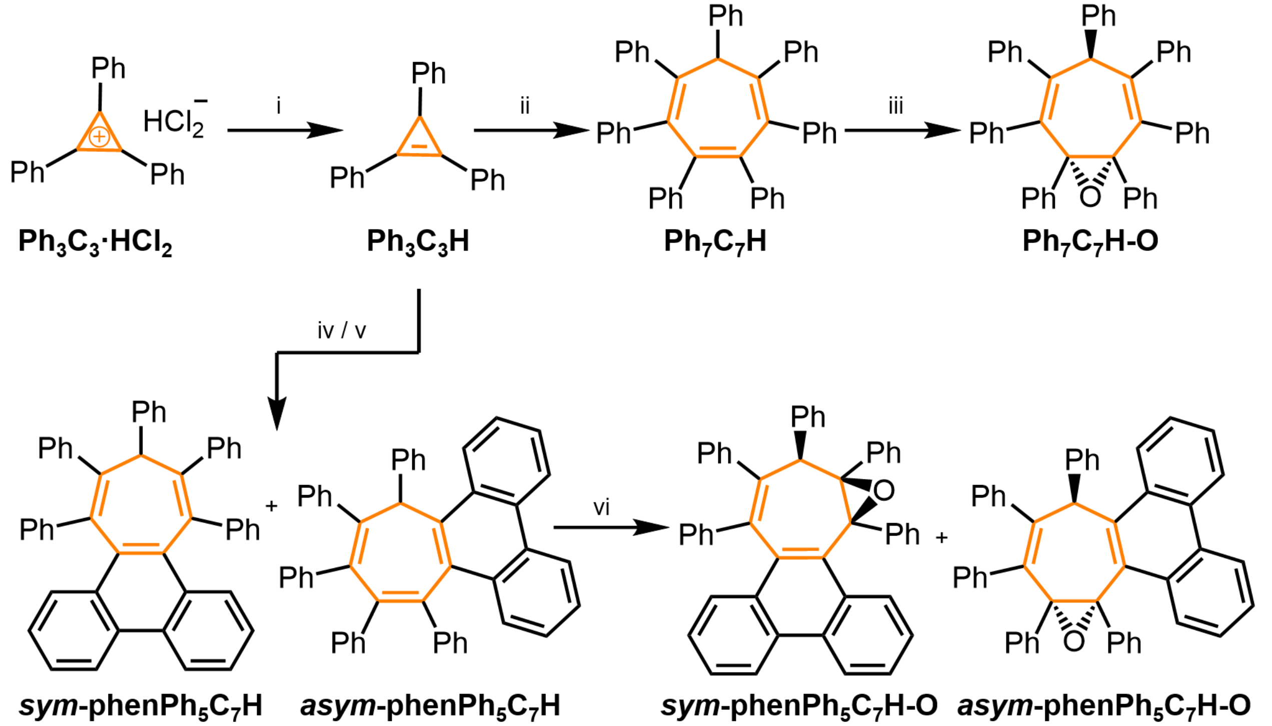

Scheme S1. Synthetic route used to prepare the functionalized cycloheptatrienes. Reagents and conditions: (i) $NaBH_4$ / EtOH / rt / 16 h / 97%; (ii) tetraphenylcyclopentadieneone / *p*-xylene / 160 °C / 24 h / 74%; (iii) *m*CPBA / $CHCl_3$ / 0 °C to 55 °C / 24 h / 70%; (iv) phencyclone / *p*-xylene / 165 °C / 66 h / 26% ***sym*-phenPh_5C_7H**; (v) phencyclone / *p*-xylene / 200 °C / 12 h / 24% ***sym*-phenPh_5C_7H**, 48% ***asym*-phenPh_5C_7H**; (vi) *m*CPBA / $CHCl_3$ / 0 °C to 55 °C / 22 h / 5% ***sym*-phenPh_5C_7H-O**, 55% ***asym*-phenPh_5C_7H-O**.

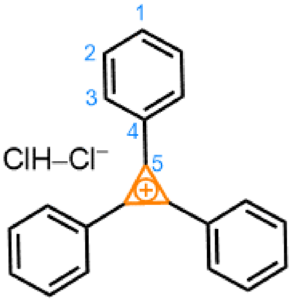

$Ph_3C_3{\cdot}HCl_2$

Triphenylcyclopropenyl hydrogen dichloride ($Ph_3C_3{\cdot}HCl_2$): Diphenylacetylene (18.5 g, 104 mmol) and potassium *tert*-butoxide (35.0 g, 312 mmol) were placed in a two-necked oven-dried 500 mL round-bottomed flask fitted with a septum, under a N_2 atmosphere. Dry benzene (200 mL) was added, and the mixture was stirred. α,α-Dichlorotoluene (25.0 g, 155 mmol) was added to the reaction mixture uniformly over a period of 30 min using a syringe pump. The reaction mixture was then heated to reflux for 3 h at 80 °C. After cooling, H_2O (200 mL) was added to dissolve the inorganic salts. The layers were separated and the aqueous layer was extracted with Et_2O (2 × 200 mL). The ether extracts were combined with the benzene layer and dried over $MgSO_4$, before the solvent was removed under

reduced pressure to give a solid, orange residue. This crude mixture was dissolved in a mixture of 2:1 Et_2O–CH_2Cl_2 (200 mL) and then sparged with gaseous HCl, leading to the formation of a colorless precipitate. Sparging was continued until no more precipitation was observed, then the mixture was filtered through a sintered glass funnel and the solid washed with Et_2O. The solid was dried under vacuum to yield **Ph_3C_3·HCl_2** as cream powder (18.6 g, 61.4 mmol, 43%). The HCl_2^- anion is evident in X-ray diffraction data obtained from single crystals (Figure S34) and a broad ^1H NMR resonance was observed with concentration-dependent chemical shift. **M.P.** 186 – 188 °C. **^1H NMR** (600 MHz, CD_3CN) δ 8.83 – 8.39 (m, 6H, H_3), 8.20 – 8.00 (m, 3H, H_1), 7.97 – 7.86 (m, 6H, H_2). **^{13}C NMR** (151 MHz, CD_3CN) δ 154.1 (C_5), 139.1 (C_1), 136.6 (C_2), 131.4 (C_3), 121.6 (C_4). **HRESI-MS** *m/z* = 267.1169 $[M]^+$ (calculated for $C_{21}H_{15}^+$ = 267.1168).

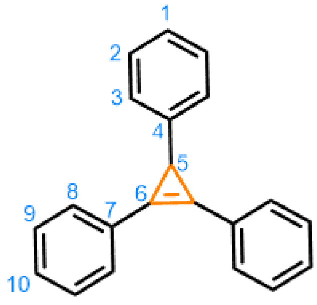

Ph_3C_3H

***sym*-Triphenylcyclopropene (Ph_3C_3H)**: Sodium borohydride (9.30 g, 246 mmol) was added to a 0 °C solution of **Ph_3C_3·HCl_2** (18.6 g, 61.4 mmol) in EtOH (460 mL). The mixture was allowed to stir overnight at rt. The reaction was quenched with H_2O (500 mL) then extracted with Et_2O (3 × 200 mL). The organic layers were combined and washed with H_2O (500 mL) and brine (500 mL). The combined organic extracts were then dried over $MgSO_4$, and the solvent was removed under reduced pressure to give **Ph_3C_3H** as a colorless solid (14.2 g, 53.1 mmol, 97%). **M.P.** 113 – 115 °C (lit.[3] 114 – 115.5 °C). **^1H NMR** (400 MHz, $CDCl_3$) δ 7.71 – 7.66 (m, 4H, H_8), 7.47 – 7.40 (m, 4H, H_9), 7.38 – 7.32 (m, 2H, H_{10}), 7.24 (br s, 2H, H_2), 7.23 – 7.22 (m, 2H, H_3), 7.17 – 7.10 (m, 1H, H_1), 3.27 (s, 1H, H_5). **^{13}C NMR** (101 MHz, $CDCl_3$) δ 144.6 (C_4), 130.0 (C_8), 128.9 (C_9), 128.8 (C_{10}), 128.7 (C_7), 128.3 (C_3), 126.0 (C_2), 125.6 (C_1), 112.7 (C_6), 24.5 (C_5). **HRESI-MS** *m/z* = 267.1172 $[M-H]^+$ (calculated for $C_{21}H_{15}^+$ = 267.1168).

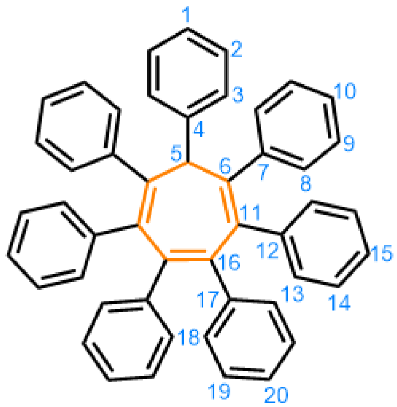

Ph$_7$C$_7$H

***sym*-Heptaphenylcycloheptatriene (Ph$_7$C$_7$H)**: To a 20 mL microwave vial was added **Ph$_3$C$_3$H** (1.2 g, 4.47 mmol), tetraphenylcyclopentadienone (1.8 g, 4.47 mmol) and anhydrous *p*-xylene (13.5 mL). The reaction vessel was sealed, and the mixture deoxygenated (3 × freeze–pump–thaw cycles under N_2) then stirred for 24 h at 160 °C in a microwave reactor. Upon cooling to rt, a crystalline solid formed, which was isolated by filtration through a sintered glass funnel, washing with Et_2O (3 × 10 mL). The solid was dried under vacuum to yield **Ph$_7$C$_7$H** as a pale pink solid (2.3 g, 3.68 mmol, 82%) in high purity (no observable impurities by ^{1}H NMR spectroscopy). Samples for UV-vis and fluorescence measurements were further purified by recrystallization. A saturated solution of **Ph$_7$C$_7$H** in 1:1 $CHCl_3$–EtOH was allowed to slowly evaporate, yielding colorless crystals. **M.P.** 291 – 292 °C (lit.[3] 285 – 287.5 °C). **^{1}H NMR** (700 MHz, $CDCl_3$) δ 8.17 – 7.99 (m, 2H, H_3), 7.62 – 7.46 (m, 2H, H_2), 7.44 – 7.34 (m, 1H, H_1), 7.20 – 7.12 (m, 4H, H_8), 7.03 – 6.96 (m, 16H, $H_{9+13+14+10+15}$), 6.70 – 6.61 (m, 6H, H_{18+20}), 6.30 – 6.21 (m, 4H, H_{19}), 5.36 (s, 1H, H_5). **^{13}C NMR** (176 MHz, $CDCl_3$) δ 143.6 (C_{16}), 143.5 (C_7), 143.4 (C_4), 140.8 (C_{12}), 140.5 (C_{17}), 139.7 (C_6), 137.1 (C_{11}), 131.7 (C_{13}), 131.4 (C_{18}), 129.9 (C_8), 128.5 (C_2), 127.7 (C_9), 127.3 (C_{14}), 126.9 (C_3), 126.9 (C_1), 126.4 (C_{10}), 126.2 (C_{19}), 126.1 (C_{15}), 125.2 (C_{20}), 57.8 (C_5). **HRESI-MS** *m/z* = 625.2892 $[M+H]^+$ (calculated for $C_{47}H_{37}$ = 625.2890).

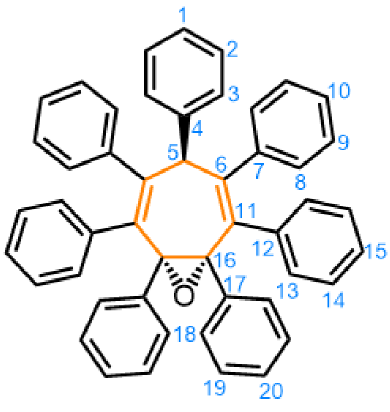

Ph_7C_7H-O: *sym*-Heptaphenylcycloheptatriene (1.0 g, 1.6 mmol) was dissolved in $CHCl_3$ (50 mL) and cooled to 0 °C. The mixture was stirred for 5 min. A cooled solution of *m*CPBA (1.6 g, 6.5 mmol) in $CHCl_3$ (20 mL) was added and stirred for a further 5 min before being allowed to warm to rt. The mixture was slowly heated to 55 °C and left to stir for 16 h. The mixture was allowed to cool to rt and a further equivalent of *m*CPBA (0.28 g, 1.6 mmol) was added. The mixture was heated to 55 °C for 3 h before allowing to cool to rt. A saturated aqueous solution of $NaHCO_3$ (20 mL) was added and the resulting biphasic mixture was stirred for 15 min. The organic layer was separated and then washed with a saturated aqueous solution of $NaHCO_3$ (2 × 20 mL) and brine (30 mL), before being dried over $MgSO_4$, filtered and evaporated to dryness to give a crude orange solid. The crude mixture was recrystallized by slow evaporation of a saturated EtOAc solution to yield the title compound as a crystalline colorless solid (0.72 g, 1.12 mmol, 70%). **M.P.** > 350 °C. **^{1}H NMR** (700 MHz, $CDCl_3$) δ 8.11 – 8.05 (m, 2H, H_3), 7.60 (t, *J* = 7.7 Hz, 2H, H_2), 7.49 – 7.43 (m, 1H, H_1), 7.14 – 7.12 (m, 4H, H_8), 7.11 – 7.08 (m, 4H, H_{13}), 7.06 – 6.98 (m, 12H, H_9, H_{10}, H_{14} and H_{15}), 6.81 – 6.75 (m, 2H, H_{20}), 6.72 – 6.66 (m, 4H, H_{19}), 6.63 – 6.52 (m, 4H, H_{18}), 5.14 (s, 1H, H_5). **^{13}C NMR** (176 MHz, $CDCl_3$) δ 146.7 (C_6), 144.6 (C_4), 144.5 (C_7), 140.4 (C_{12}), 138.8 (C_{11}), 137.4 (C_{17}), 131.0 (C_{13}), 129.0 (C_8), 128.9 (C_2 or C_3), 128.8 (C_2 or C_3), 128.8 (C_{18}), 128.0 (C_9 or C_{14}), 127.4 (C_9 or C_{14}), 127.2 (C_1), 126.6 (C_{10} or C_{15} or C_{20}), 126.5 (2C, C_{10} and/or C_{15} and/or C_{20}), 126.4 (C_{19}), 74.7 (C_{16}), 58.4 (C_5). **HRASAP-MS** *m/z* = 641.2834 $[M+H]^+$, calculated for $C_{49}H_{37}O^+$: 641.2839.

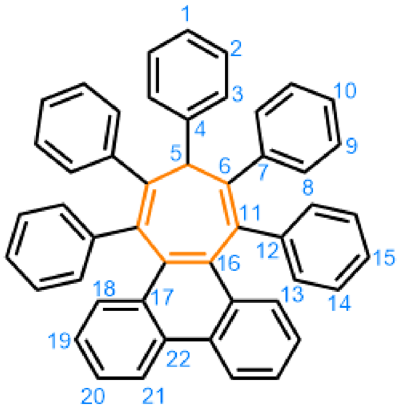

***sym*-phenPh$_5$C$_7$H**

***sym*-phenPh$_5$C$_7$H**: Phencyclone (0.71 g, 1.86 mmol), *sym*-triphenylcyclopropene (0.50 g, 1.86 mmol) and anhydrous *p*-xylene (30 mL) were added to a 100 mL round bottom flask. The mixture was deoxygenated (3 × freeze-pump-thaw cycles under argon) before being heated to reflux at 165 °C for 66 h. The mixture was allowed to cool to rt, producing a white precipitate. Me_2CO (20 mL) was added to induce further precipitation. The solid was isolated by filtration and washed with cold *p*-xylene (3 mL), then dried under vacuum to give ***sym*-phenPh$_5$C$_7$H** as a colorless solid (0.30 g, 0.48 mmol, 26%). Where possible, ^{1}H and ^{13}C NMR resonances have been assigned using bidimensional NMR techniques. However, some resonances could not be assigned because of overlap between peaks and the similarity of some nuclear environments. **M.P.** 318 – 321 °C. **^{1}H NMR** (700 MHz, 1:7 CD_2Cl_2–CS_2) δ 8.31 (d, *J* = 8.3, 1.2 Hz, 2H, H_{21}), 7.82 (d, *J* = 8.4, 1.2 Hz, 2H, H_{18}), 7.46 (d, *J* = 8.1, 1.2 Hz, 2H, H_3), 7.35 – 7.29 (m, 2H, H_{20}), 7.15 – 7.11 (m, 4H, $H_{8\text{-}10}$ or $H_{13\text{-}15}$), 7.11 – 7.08 (m, 2H, H_{19}), 7.08 – 7.04 (m, 11H, $H_{8\text{-}10}$ or $H_{13\text{-}15}$), 7.04 – 7.02 (m, 5H, $H_{8\text{-}10}$ or $H_{13\text{-}15}$), 6.63 – 6.58 (m, 2H, H_2), 6.56 – 6.53 (m, 1H, H_1), 5.21 (s, 1H, H_5). **^{13}C NMR** (176 MHz, 1:7 CD_2Cl_2–CS_2) δ 146.4, 143.1, 143.1, 140.0 (C_4), 137.3 (C_{16}), 133.8, 131.2, 130.3 (C_{17}), 130.3 (C_{18}), 129.9, 129.4 (C_{22}), 128.0, 127.8 (C_2), 127.3, 126.7, 126.0, 125.8 (C_{20}), 125.5 (C_1), 125.4 (C_{19}), 124.8 (C_3), 122.0 (C_{21}), 60.5 (C_5). **HRASAP-MS** *m/z* = 622.2685 $[M]^+$, calculated for $C_{49}H_{34}^+$: 622.2655.

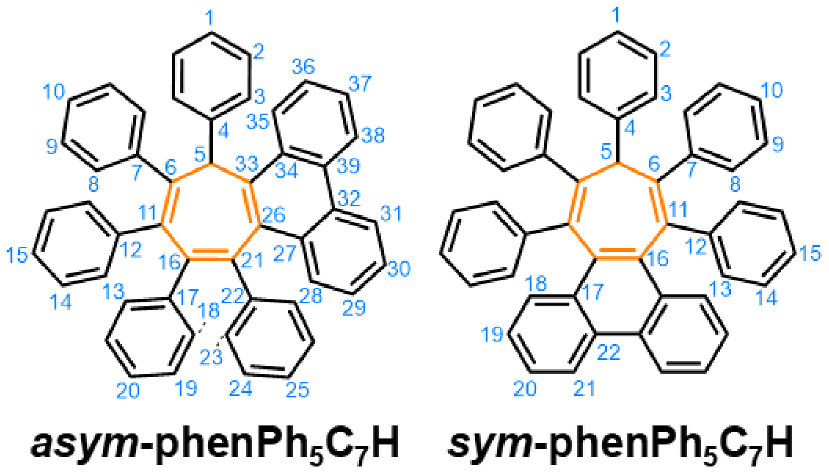

A 2:1 mixture of ***asym*-phenPh$_5$C$_7$H** and ***sym*-phenPh$_5$C$_7$H**: *sym*-Triphenylcyclopropene (297 mg, 1.10 mmol), phencyclone (424 mg, 1.10 mmol) and *p*-xylene (2.20 mL) were added to a microwave vial. The vial was sealed with a septum and the mixture degassed (3 × freeze-pump-thaw cycles under argon), then stirred for 12 h at 200 °C in a microwave reactor. Upon cooling, a precipitate formed, which was isolated by filtration, washing with cold *p*-xylene (3 × 5 mL). This solid was dried under vacuum to yield a light-yellow solid, which was recrystallized by slow evaporation of a 2:1 CH_2Cl_2–EtOH solution to give the first batch of a mixture of ***asym*-phenPh$_5$C$_7$H** and ***sym*-phenPh$_5$C$_7$H** as a colorless solid (262 mg, 0.421 mmol, 38%). Separately, the filtrate was concentrated under reduced pressure and the resulting residue was purified by column chromatography (Teledyne Isco CombiFlash Rf+ system, 40 g SiO_2, petroleum ether−EtOAc, gradient elution). A fraction containing a mixture of ***asym*-phenPh$_5$C$_7$H** and ***sym*-phenPh$_5$C$_7$H** was isolated, which was concentrated under reduced pressure yielding a colorless solid (84 mg, 0.14 mmol, 12%). A second fraction was isolated containing the desired products along with impurities. This fraction was further purified by recrystallization by slow evaporation of a 5:3 CH_2Cl_2–EtOH solution, yielding a third batch of ***asym*-phenPh$_5$C$_7$H** and ***sym*-phenPh$_5$C$_7$H** as a colorless solid (181 mg, 0.29 mmol, 26%). The three batches were combined, giving ***asym*-**

phenPh$_5$C$_7$H and ***sym*-phenPh$_5$C$_7$H** (527 mg, 0.76 mmol, 72% overall yield). **^{1}H NMR** spectroscopic analysis (Figure S11) showed the two isomers were present in a 2:1 ratio of ***asym*-phenPh$_5$C$_7$H** to ***sym*-phenPh$_5$C$_7$H**. This ratio corresponds to a statistical mixture, as ***asym*-phenPh$_5$C$_7$H** is present as a racemic mixture of *R*- and *S*-stereoisomers. **M.P.**: 288 – 290 °C. **HRASAP-MS** *m/z* = 622.2668 $[M]^+$, calculated for $C_{49}H_{34}^+$: 622.2655.

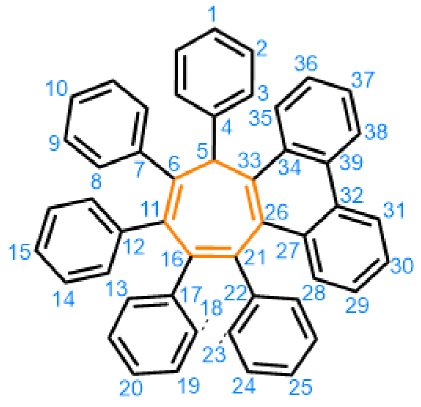

***asym*-phenPh$_5$C$_7$H:** A pure sample of ***sym*-phenPh$_5$C$_7$H** (300 mg, 0.48 mmol) was placed in a sealed 5 mL vial. The solid was heated using a heat gun until a melt formed (~320 °C), before being allowed to cool to rt and solidify. This heat/cool cycle was repeated five times. The resulting black solid was sonicated in Me_2CO (4 mL), and the supernatant was collected. Evaporating to dryness to give a crude yellow solid of a 6:1 mixture of *asym*:*sym* isomers. The crude solid was purified by column chromatography (Teledyne Isco CombiFlash Rf+ system**,** high performance 24 g SiO_2, hexanes). Pure factions were identified by TLC and combined. The title compound was isolated as a colorless crystalline solid of the ***asym*-phenPh$_5$C$_7$H** isomer (5 mg, 8.0 μM, 1.7%). The remaining solid was isolated as a mixture of the *asym:sym* isomers. **M.P.** 302 – 304 °C. **^{1}H NMR** (700 MHz, $CDCl_3$) δ 8.76 (dd, *J* = 8.4, 1.2 Hz, 2H, H_{28}), 8.64 (dd, *J* = 8.4, 1.2 Hz, 1H, H_{38}), 8.16 (d, *J* = 8.4 Hz, 2H, H_{31}), 7.96 – 7.92 (m, 1H, H_{35}), 7.66 (ddd, *J* = 8.2, 6.9, 1.2 Hz, 1H, H_{29}), 7.58 (d, *J* = 7.6 Hz, 2H, H_{18}), 7.55 (m, 1H, H_{30}), 7.46 (m, 1H, H_{37}), 7.28 (m, 2H, H_{19}), 7.25 (m, 1H, H_{36}), 7.23 (m, 1H, H_{20}), 7.11 (m, 3H, H_{13+15}), 7.02 – 6.95 (m, 5H, $H_{8\text{-}10}$), 6.86 (m, 1H, H_1), 6.78 (m, 1H, H_{25}), 6.73 (m, 2H, H_{25}), 6.71 (m, 2H, H_{23}), 6.68 (m, 2H, H_2), 6.36 (s, 1H, H_5), 5.70 (d, *J* = 7.7 Hz, 2H, H_3). **^{13}C NMR** (176 MHz, $CDCl_3$) δ 143.8 (C_6),

143.5 (C_{26}), 143.4 (C_{16}), 143.1 (C_{11}), 142.7 (C_{21}), 142.5 (C_{33}), 142.1 (C_4), 140.7 (C_{22}), 140.2 (C_{17}), 139.5 (C_{12}), 138.4 (C_7), 138.0 (C_{39}), 132.2 (C_{32}), 131.7 (C_{23}), 131.5 (C_{34}), 131.5 (C_{27}), 131.2 (C_3), 129.5 (C_{35}), 129.43 (C_{14}), 129.0 ($C_{8 \text{ or } 9 \text{ or } 10}$), 128.7 ($C_{36}$), 127.7 ($C_{8 \text{ or } 9 \text{ or } 10}$), 127.67 ($C_{15}$), 127.6 ($C_{30}$), 127.1 ($C_{18}$), 126.9 ($C_{29}$), 126.79 ($C_{24}$), 126.7 ($C_{13}$), 126.5 ($C_{20}$), 126.5 ($C_2$), 126.3 ($C_{8 \text{ or } 9 \text{ or } 10}$), 125.9 ($C_{19}$), 125.8 ($C_{37}$), 125.7 ($C_{25}$), 125.7 ($C_1$), 124.4 ($C_{31}$), 123.2 ($C_{28}$), 122.7 ($C_{38}$), 50.3 ($C_5$). **HR-ASAP MS** *m/z* = 622.2662 $[M]^+$, calculated for $C_{49}H_{34}^+$: 622.2655.

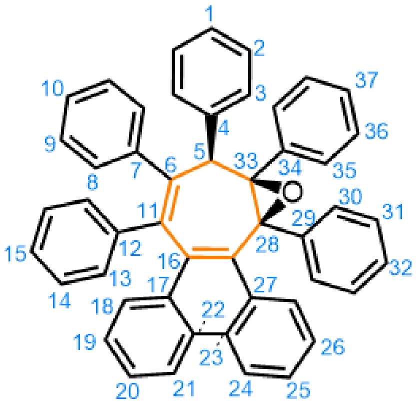

***sym*-phenPh$_5$C$_7$H-O**

***sym*-phenPh$_5$C$_7$H-O:** An isomerically pure sample of ***sym*-phenPh$_5$C$_7$H** (400 mg, 0.64 mmol) was dissolved in $CHCl_3$ (20 mL) and cooled to 0 °C before adding a saturated aqueous solution of $NaHCO_3$ (0.5 mL). Solid *m*CPBA (0.8 g, 4.6 mmol) was added. The biphasic mixture was heated to 70 °C and left to stir for 22 h before allowing to cool to rt. A saturated aqueous solution of $NaHCO_3$ (30 mL) was added and the resulting biphasic mixture was stirred for 15 min. The reaction mixture was diluted with $CHCl_3$ (50 mL) and the organic layer was separated and washed with a saturated aqueous solution of $NaHCO_3$ (2 × 25 mL) and brine (5 mL), before being dried over $MgSO_4$, filtered and evaporated to dryness to give a crude yellow solid. The crude solid was purified by column chromatography (Teledyne Isco CombiFlash Rf+ system, 24 g SiO_2, hexanes–CH_2Cl_2, 0 – 25% gradient elution). The title compound was isolated as a colorless crystalline solid (30 mg, 0.048 mmol, 8%) **M.P.** > 350 °C. **^{1}H NMR** (700 MHz, $CDCl_3$) δ ^{1}H NMR (700 MHz, $CDCl_3$) δ 8.45 (m, 2H, H_{18+27}), 8.05 (d, *J* = 8.4 Hz, 1H, H_{24}), 8.00 (d, *J* = 8.4 Hz, 1H, H_{25}), 7.49 – 7.45 (m, 2H, H_{37}), 7.45 – 7.39 (m, 2H, H_{19+26}), 7.31 – 7.25 (m, 6H, $H_{3+20+25+32}$), 7.23 (m, 2H, H_{14}), 7.14 (m, 2H, H_8), 7.10 (m, 3H, H_{1+2}), 7.08 – 7.03 (m, 6H,

$H_{9+10+13+15}$), 7.00 (m, 3H, H_{33+34}), 6.72 (m, 2H, H_{38}), 6.66 (m, 1H, H_{39}), 4.90 (s, 1H, H_5). **^{13}C NMR** (176 MHz, $CDCl_3$) δ 143.8 (C_{11}), 143.6 (C_{16}), 142.5 (C_6), 140.9 (C_{12}), 140.1 (C_{31}), 138.8 (C_{36}), 136.4 (C_7), 135.9 (C_4), 131.5 (C_{28}), 131.5 (C_{23}), 131.1 (C_{17}), 130.7(C_{22}), 129.9 (C_{29}), 129.6 (C_8), 129.5 (C_{21}), 129.4 (C_{20}), 128.6 (C_{37}), 128.2 (C_{24}), 127.9 (C_2), 127.9 ($C_{9\text{ or }10}$), 127.8 ($C_{9\text{ or }10}$), 127.6 (C_3), 127.6 (C_{15}), 127.2 (C_{14}), 127.1 (C_{13}), 126.9 (C_{38}), 126.9 (C_{33}), 126.9 (C_{34}), 126.7 (C_1), 126.5 (C_{25}), 126.4 (C_{19}), 126.3 (C_{26}), 125.6 (C_{32}), 125.4 (C_{39}), 122.5 (C_{18}), 122.2 (C_{27}), 78.9 (C_{35}), 66.3 (C_{30}), 58.0 (C_5). **HR-ESI MS** *m/z* = 639.2664 [M+H]$^+$, calculated for $C_{49}H_{35}O^+$: 639.2688.

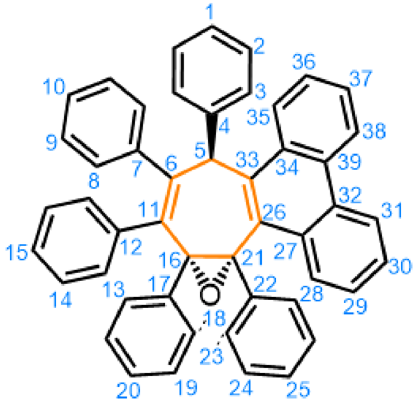

***asym*-phenPh$_5$C$_7$H-O**

***asym*-phenPh$_5$C$_7$H-O**: The previously obtained 2:1 mixture of ***asym*-phenPh$_5$C$_7$H** and ***sym*-phenPh$_5$C$_7$H** (20 mg, 32 μmol) was dissolved in $CHCl_3$ (0.5 mL). Solid *m*CPBA (40 mg, 230 μmol) was added. The mixture was heated to 70 °C and left to stir for 22 h before allowing to cool to rt. A saturated aqueous solution of $NaHCO_3$ (2 mL) was added and the resulting biphasic mixture was stirred for 15 min. The reaction mixture was diluted with $CHCl_3$ (5 mL) and the organic layer was separated and then washed with a saturated aqueous solution of $NaHCO_3$ (2 × 5 mL) and brine (5 mL), before being dried over $MgSO_4$, filtered and evaporated to dryness to give a crude off-white solid. The crude mixture was purified by column chromatography (Teledyne Isco CombiFlash Rf+ system, 24 g SiO_2, hexanes–CH_2Cl_2, 0 – 30% gradient elution). The title compound was isolated as a colorless crystalline solid (5 mg, 8 μmol, 25%). **M.P.** > 350 °C. **^{1}H NMR** (700 MHz, $CDCl_3$) δ 8.82 (dd, *J* = 8.4, 1.2 Hz, 1H, H_{38}), 8.72 (d, *J* = 8.3 Hz, 1H, H_{31}), 8.22 (dd, *J* = 8.3, 1.2 Hz, 1H, H_{28}), 8.16 (d, *J* = 8.4 Hz, 1H, H_{35}), 7.76 – 7.67 (m, 3H, H_{3+37}), 7.61 (ddd, *J* = 8.3, 6.9, 1.3 Hz, 1H, H_{36}), 7.57 (ddd, *J* = 8.3, 6.9, 1.3 Hz, 1H, H_{29}), 7.45

(m, 3H, H_{1+2}) 7.31 (m, 1H, H_{10}), 7.10 – 6.94 (m, 12H, $H_{8+9+13+14+23+24}$), 6.91 (tt, J = 7.4, 1.3 Hz, 1H, H_{15}), 6.84 – 6.75 (m, 1H, H_{25}), 6.78 – 6.64 (m, 6H, $H_{18+19+20,30}$), 6.41 (s, 1H, H_5). **^{13}C NMR** (176 MHz, $CDCl_3$) δ 145.8 (C_{11}), 144.4 (C_{33}), 143.2 (C_4), 140.1 (C_{22}), 139.5(C_{17}), 139.1 (C_7), 138.9 (C_6), 137.9 (C_{27}), 135.6 (C_{32}), 132.5 (C_{39}), 132.3 (C_8), 132.3 (C_9), 131.3 (C_{14}), 130.9 (C_{26}), 130.9 (C_{34}), 130.5 (C_{12}), 128.9 (C_{24}), 128.8 (C_3), 128.7 (C_{13}), 128.2 ($C_{18\ or\ 20}$), 128.1 (C_2), 127.8 (C_{28}), 127.4 (C_{36}), 127.1 (C_{23}), 126.9 (C_{37}), 126.83 (C_{15}), 126.8 (C_{10}), 126.6 (C_1), 126.5 ($C_{20\ or\ 18}$), 126.5 (C_{25}), 126.4 (C_{29}), 126.23 (C_{19+37}), 124.7 (C_{30}), 123.3 (C_{35}), 122.6 (C_{38}), 122.5(C_{31}) 74.2 (C_{16}), 71.9 (C_{21}), 50.5 (C_5). **HR-ESI MS** *m/z* = 639.2671 $[M+H]^+$, calculated for $C_{49}H_{35}O^+$: 639.2688.

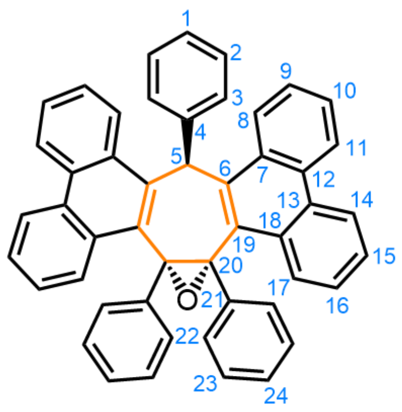

sym-phen$_2$Ph$_3$C$_7$H-O

***sym*-phen$_2$Ph$_3$C$_7$H-O**: **Ph$_7$C$_7$H-O** (55 mg, 90 μmol) and I_2 (51 mg, 20 μmol, 2.1 equiv) were added to a 7 mL quartz tube, which was fitted with a septum and purged with $N_{2(g)}$. Anhydrous THF (6 mL) was deoxygenated (3 × freeze-pump-thaw cycles under argon). The mixture was sparged with $N_{2(g)}$ for 10 min followed by the addition of propylene oxide (0.5 mL) and sparging with $N_{2(g)}$ for a further 5 min. The reaction mixture was then irradiated by 4 × 9W 254 nm bulbs for 3 h, while being sparged with $N_{2(g)}$. A saturated aqueous solution of $Na_2S_2O_3$ (2 mL) was added and the resulting biphasic mixture was stirred for 2 min. The reaction mixture was diluted with $CHCl_3$ (5 mL) and the organic layer was separated and then washed with a saturated aqueous solution of $Na_2S_2O_3$ (2 × 5 mL) and brine (5 mL), before being dried over $MgSO_4$, filtered and evaporated to dryness to give a dark solid. The crude solid was purified by column chromatography (Teledyne Isco CombiFlash Rf+ system, 12 g SiO_2, hexanes–CH_2Cl_2, 0 – 30% gradient elution). The title compound was isolated as a colorless

crystalline solid (54 mg, 90 µmol, >99%). **M.P.** > 350 °C. **^{1}H NMR** (600 MHz, $CDCl_3$) δ 8.80 (m, 2H, H_8), 8.72 (m, 2H, H_{14}), 8.61 (m, 2H, H_{11}), 8.52 (m, 2H, H_{17}), 7.91 (s, 1H, H_5), 7.72 (m, 2H, H_{16}), 7.67 (m, 2H, H_{17}), 7.54 (m, 6H, H_{2+9+10}), 7.32 (m, 2H, H_3), 7.29 (m, 1H, H_1), 7.18 (m, 4H, H_{22}), 6.85 (m, 2H, H_{24}), 6.80 (m, 4H, H_{23}).**^{13}C NMR** (151 MHz, $CDCl_3$) δ 141.8 (C_6), 138.2 (C_4), 137.4 (C_{21}), 133.4 (C_{18}), 133.2 (C_{13}), 131.4 (C_{12}), 130.9 (C_{19}), 130.9 (C_7), 129.9 (C_9), 129.4 (C_{22}), 128.5 (C_3), 127.9 (C_{16}), 127.9 (C_8), 127.9 (C_2), 126.9 (C_{24}), 126.9 (C_{10}), 126.8 (C_{23}), 126.7 (C_{15}), 126.7 (C_1), 124.7 (C_{17}), 123.4 (C_{14}), 122.6 (C_{11}), 71.3 (C_{20}), 44.0 (C_5). **HRMS-ASAP** *m/z* = 637.2505 $[M+H]^+$, calculated for $C_{49}H_{33}O^+$: 637.2531.

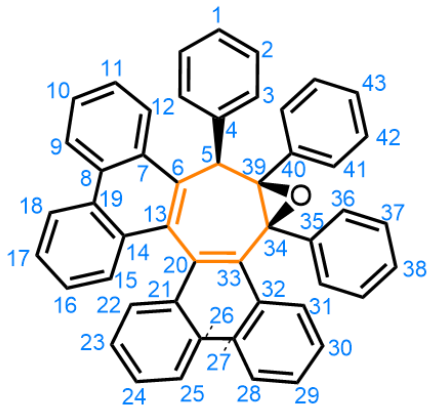

***asym*-phen$_2$Ph$_3$C$_7$H-O**

***asym*-phenPh$_5$C$_7$H-O:** ***sym*-phenPh$_5$C$_7$H-O** (30 mg, 46 µmol) and iodine (13 mg, 51 µmol, 1.1 equiv) were added to a 10 mL quartz tube, which was fitted with a septum and sparged wit h $N_{2(g)}$. Anhydrous THF (8 mL) was degassed through 3 × freeze-pump-thaw cycles and added. The mixture was sparged with $N_{2(g)}$ for 10 min followed by the addition of propylene oxide (1 mL) and sparging with $N_{2(g)}$ for a further 5 min. The reaction mixture was then irradiated by 4 × 9W 254 nm bulbs for 2 h, while being sparged with $N_{2(g)}$. A saturated aqueous solution of $Na_2S_2O_3$ (5 mL) was added and the resulting biphasic mixture was stirred for 2 min. The reaction mixture was diluted with $CHCl_3$ (5 mL) and the layers were separated. The organic layer was washed with a saturated aqueous solution of $Na_2S_2O_3$ (2 × 10 mL) and brine (10 mL), before being dried over $MgSO_4$, filtered and evaporated to dryness to give a yellow solid. The crude solid was washed with MeOH (5 mL) and Et_2O (5 mL) to afford the title compound as a colorless crystalline solid (29 mg, 45 µmol, 98%). **^{1}H NMR** (600 MHz, $CDCl_3$): δ 8.90 (d, *J* = 8.4 Hz, 1H, H_{14}), 8.88 (d, *J* = 8.4 Hz, 1H, H_{11}), 8.56 – 8.51 (m, 2H, $H_{25,28}$), 8.15 – 8.11 (m, 1H, H_{31}),

7.77 – 7.69 (m, 3H, $H_{8,15,17}$), 7.67 – 7.63 (m, 1H, H_{10}), 7.54 – 7.48 (m, 2H, $H_{24,29}$), 7.48 – 7.44 (m, 1H, H_{16}), 7.44 – 7.40 (m, 1H, H_{30}), 7.40 – 7.36 (m, 1H, H_9), 7.21 – 7.17 (m, 1H, H_{23}), 7.12 – 7.08 (m, 3H, $H_{3,22}$), 7.07 – 7.03 (m, 2H, H_{36}), 7.03 – 6.53 (m, 8H, $H_{37,38,41–43}$), 6.52 (t, J = 7.2 Hz, H_1), 6.49 – 6.44 (m, 2H, H_2), 5.88 (s, 1H, H_5). **^{13}C NMR** (151 MHz, $CDCl_3$): δ 140.3 (C_6), 138.8 (C_{35}), 137.7 (C_7), 136.8 (C_4), 134.3 (C_{19}), 134.2 (C_{20}), 133.0 (C_{18}), 132.5 (C_{13}), 131.3 (C_{33}), 130.6 (C_{26}), 130.3 (C_{27}), 130.1 ($C_{21/40}$), 130.1 ($C_{40/21}$), 129.8 ($C_{12/32}$), 129.8 ($C_{32/12}$), 129.1 (C_{22}), 128.1 (C_{17}), 128.0 (C_3), 127.5 ($C_{41/42/43}$), 127.4 ($C_{42/41/43}$), 127.3 ($C_{43/42/41}$), 127.2 (C_9), 127.0 (C_{31}), 126.8 (C_{10}), 126.8 ($C_{15/16}$), 126.8 ($C_{16/15}$), 126.8 ($C_{30/38}$), 126.8 ($C_{38/30}$), 126.6 (C_{37}), 126.6 (C_{36}), 126.6 ($C_{24/29}$), 126.6 ($C_{29/24}$), 126.5 (C_2), 126.1 (C_{23}), 125.0 (C_1), 124.3 (C_8), 123.3 (C_{14}), 123.2 (C_{11}), 122.6 ($C_{25/28}$), 122.4 ($C_{28/25}$), 79.9 (C_{39}), 66.9 (C_{34}), 49.0 (C_5). **HRMS-ASAP** *m/z* = 637.2538 $[M+H]^+$, calculated for $C_{49}H_{33}O^+$: 637.2531

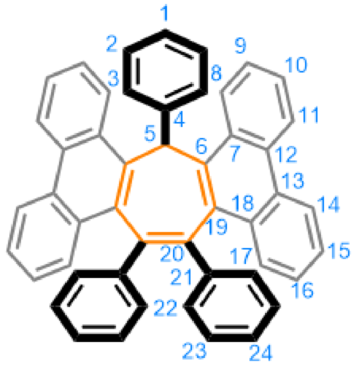

***sym*-phen₂Ph₃C₇H**

***sym*-phen$_2$Ph$_3$C$_7$H**: To a stirred solution of ***sym*-phen$_2$Ph$_3$C$_7$H-O** (50 mg, 0.079 mmol) in anhydrous THF (3 mL) was added a solution of $LiAlH_4$ (1 M in THF, 0.40 mL, 0.40 mmol) under an $N_{2(g)}$ atmosphere, and the mixture was heated to refluxed for 48 h. The mixture was then allowed to cool to rt and another aliquot of $LiAlH_4$ (1 M in THF, 0.40 mL, 0.40 mmol) was added. The mixture was refluxed for an additional 24 h. Upon cooling to rt, the reaction was quenched by the dropwise addition of a saturated aqueous solution of NH_4Cl (10 mL) and extracted with CH_2Cl_2 (5 × 5 mL). The combined organic extracts were dried over MgSO4, filtered and evaporated to dryness to give a pale-yellow solid. The crude material was washed with cold Et2O (3 × 5 mL) to yield the title compound as a colorless solid (23 mg, 0.037 mmol, 47%). **M.P.** > 350 °C. **^{1}H NMR** (600 MHz, $CDCl_3$) δ 8.67 (d, J = 8.2 Hz, 2H, H_{11}), 8.56 (d, J = 8.4 Hz, 2H, H_8), 8.54 (d, J = 8.3 Hz, 2H, H_{14}),

8.17 (d, J = 8.2 Hz, 2H, H_{17}), 7.72 – 7.66 (m, 2H, H_9), 7.66 – 7.60 (m, 2H, H_{10}), 7.49 – 7.43 (m, 3H, $H_{5,15}$), 7.42 – 7.37 (m, 2H, H_{16}), 7.13 (d, J = 7.7 Hz, 2H, H_3), 7.06 – 7.00 (m, 1H, H_1), 6.99 – 6.89 (m, 4H, $H_{2,24}$), 6.86 – 6.74 (m, 8H, $H_{22,23}$). **^{13}C NMR** (151 MHz, $CDCl_3$): δ 142.3 (C_4), 142.2 (C_6), 140.6 ($C_{20/21}$), 140.4 ($C_{21/20}$), 135.5 (C_{19}), 132.1 (C_{18}), 131.9 (C_7), 131.4 (C_{22}), 130.6 (C_{12}), 130.3 (C_{13}), 129.2 (C_{17}), 128.6 (C_2), 127.7 (C_9), 127.4 (C_{23}), 127.1 (C_3), 126.6 (C_{24}), 126.5 (C_{10}), 126.1 (C_{16}), 126.1 (C_1), 126.0 (C_{15}), 123.6 (C_8), 123.4 (C_{11}), 122.7 (C_{14}), 43.3 (C_5). **HRMS-ASAP** *m/z* = 621.2567 $[M+H]^+$, calculated for $C_{49}H_{33}^+$: 621.2582.

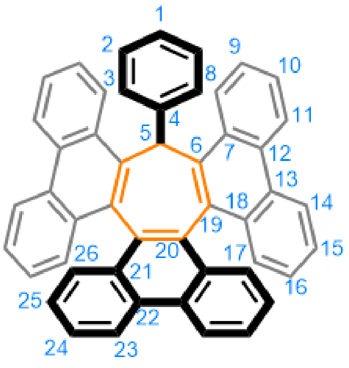

***sym*-phen$_3$PhC$_7$H**

***sym*-phen$_3$PhC$_7$H**: ***sym*-Phen$_2$Ph$_3$C$_7$H** (15 mg, 24 µmol) and I_2 (6.5 mg, 25 µmol) were added to a 4 mL quartz cuvette, which was fitted with a septum and sparged with $N_{2(g)}$. Anhydrous THF (1.2 mL) was deoxygenated (5 × freeze-pump-thaw cycles) and added. The mixture was sparged with $N_{2(g)}$ for 10 min followed by the addition of propylene oxide (0.1 mL, 2.1 mmol) and sparging with $N_{2(g)}$ for a further 5 min. The reaction mixture was then irradiated by 4 × 9W 254 nm bulbs for 15 min, while being sparged with $N_{2(g)}$. A saturated aqueous solution of $Na_2S_2O_3$ (5 mL) was added and the resulting biphasic mixture was stirred for 2 min. The reaction mixture was diluted with $CHCl_3$ (5 mL) and the organic layer was separated and then washed with a saturated aqueous solution of $Na_2S_2O_3$ (2 × 5 mL) and brine (5 mL), before being dried over $MgSO_4$, filtered and evaporated to dryness to give a colorless solid (12 mg, 20 µmol, 81%). **M.P.** > 350°C. **^{1}H NMR** (500 MHz, $CDCl_3$, 313 K) δ 8.77 (d, J = 8.3 Hz, 2H, H_{11}), 8.70 (d, J = 8.3 Hz, 2H, H_8), 8.62 (d, J = 8.4 Hz, 2H, H_{14}), 8.54 (d, J = 8.4 Hz, 2H, H_{23}), 7.77 – 7.71 (m, 2H, H_9), 7.71 – 7.66 (m, 2H, H_{10}), 7.66 – 7.61 (m, 2H, H_{17}), 7.53 (s, 1H, H_5), 7.51 – 7.44 (m, 4H, $H_{15,24}$), 7.29 – 7.21 (m, 4H, $H_{16,26}$),

7.21 – 7.15 (m, 2H, H_{25}), 6.75 (d, *J* = 7.7 Hz, 2H, H_3), 6.36 (t, *J* = 7.4 Hz, 1H, H_1), 6.27 – 6.20 (m, 2H, H_2). **^{13}C NMR** (126 MHz, $CDCl_3$, 313 K): δ 144.3 (C_6), 140.2 (C_4), 135.1 ($C_{19/20}$), 132.9 (C_{18}), 132.0 ($C_{20/19}$), 131.5 (C_7), 130.8 (C_{12}), 130.6 (C_{21}), 130.0 (C_{22}), 129.5 (C_{26}), 129.4 (C_{13}), 128.9 (C_{17}), 127.5 (C_9), 126.7 (C_{10}), 126.6 (C_2), 126.5 (C_{16}), 126.3 (C_{24}), 126.2 (C_{15}), 125.9 (C_{25}), 125.4 (C_3), 124.7 (C_1) 123.8 (C_8), 123.6 (C_{11}), 122.7 (C_{14}), 122.4 (C_{23}), 43.3 (C_5). **HRMS-ASAP** *m/z* = 619/2435 $[M+H]^+$, calculated for $C_{49}H_{31}^+$: 619.2426.

3. ^{1}H and ^{13}C NMR Spectroscopic Characterization of Synthesized Compounds

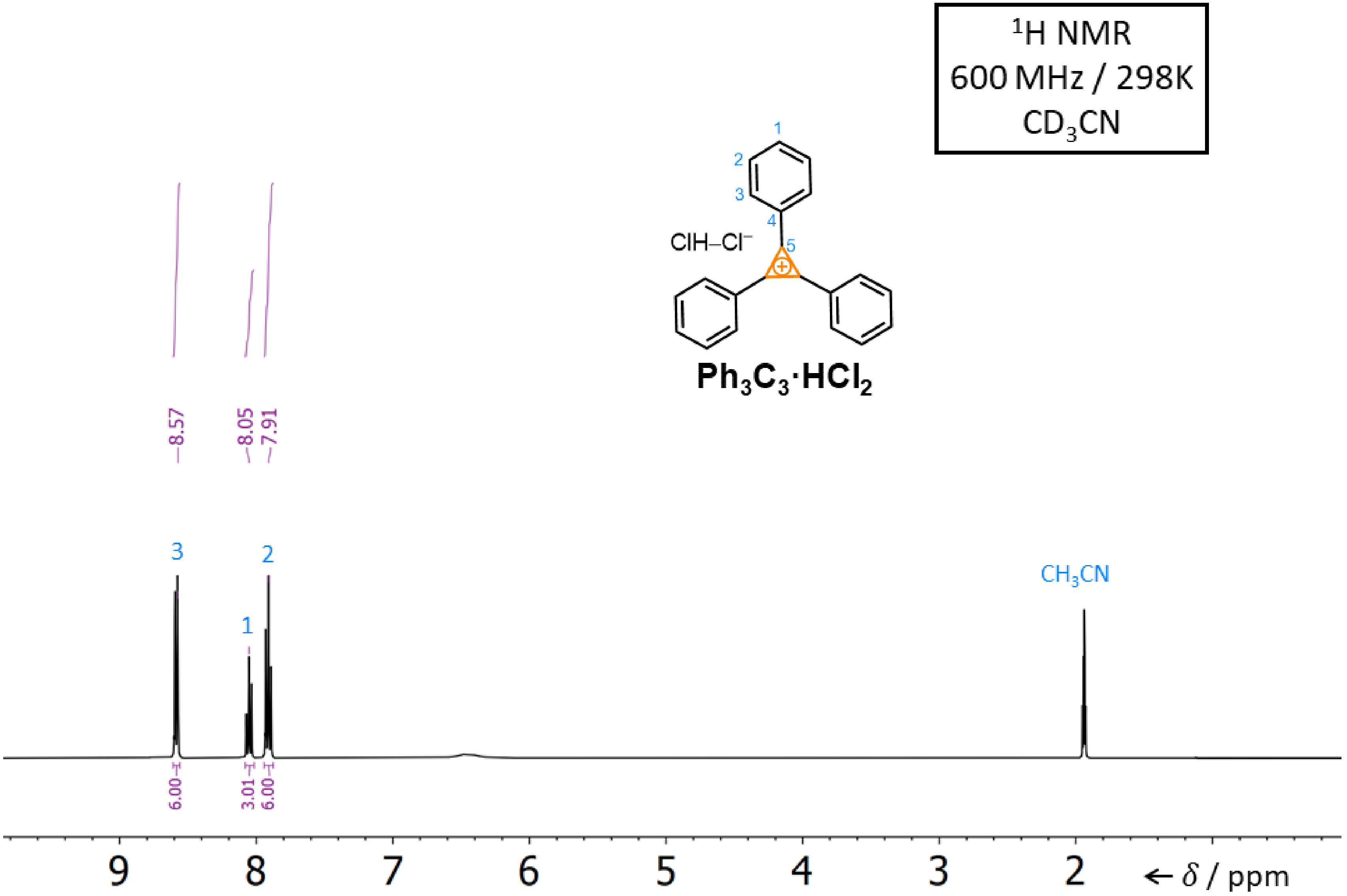

Figure S1. ^{1}H NMR Spectrum of **$Ph_3C_3·HCl_2$**.

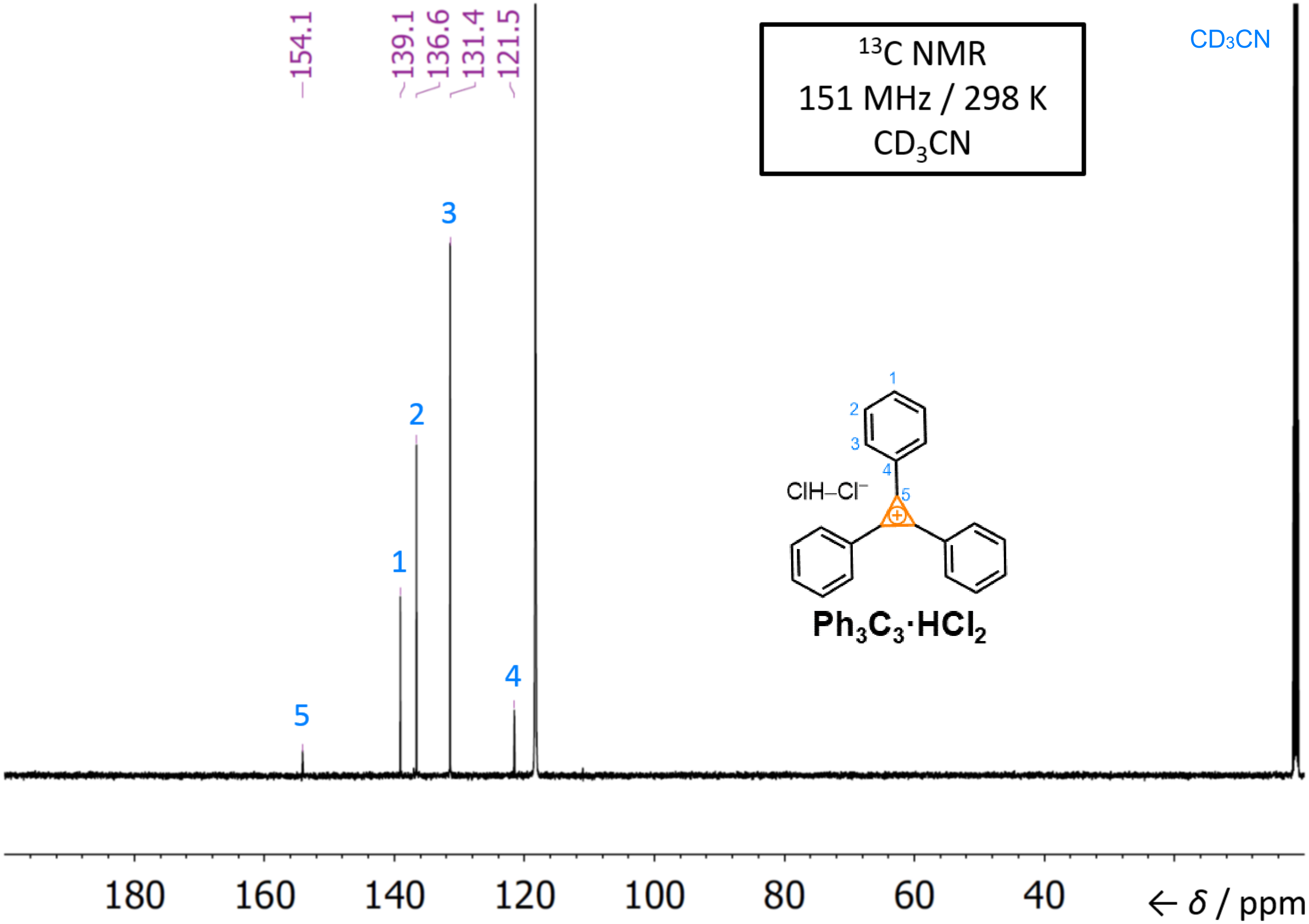

Figure S2. ^{13}C NMR Spectrum of **$Ph_3C_3{\cdot}HCl_2$**.

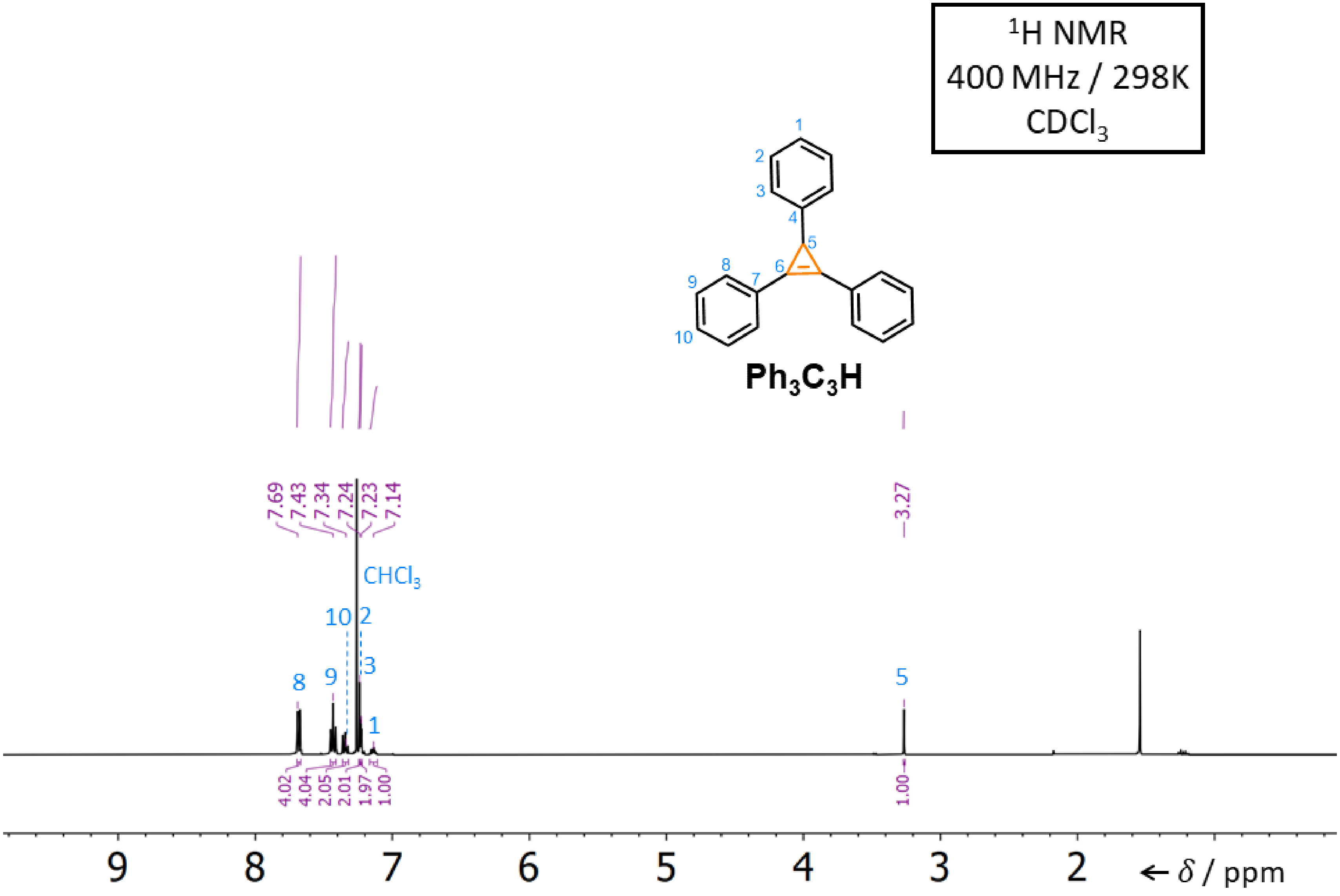

Figure S3. ^{1}H NMR Spectrum of **Ph_3C_3H**.

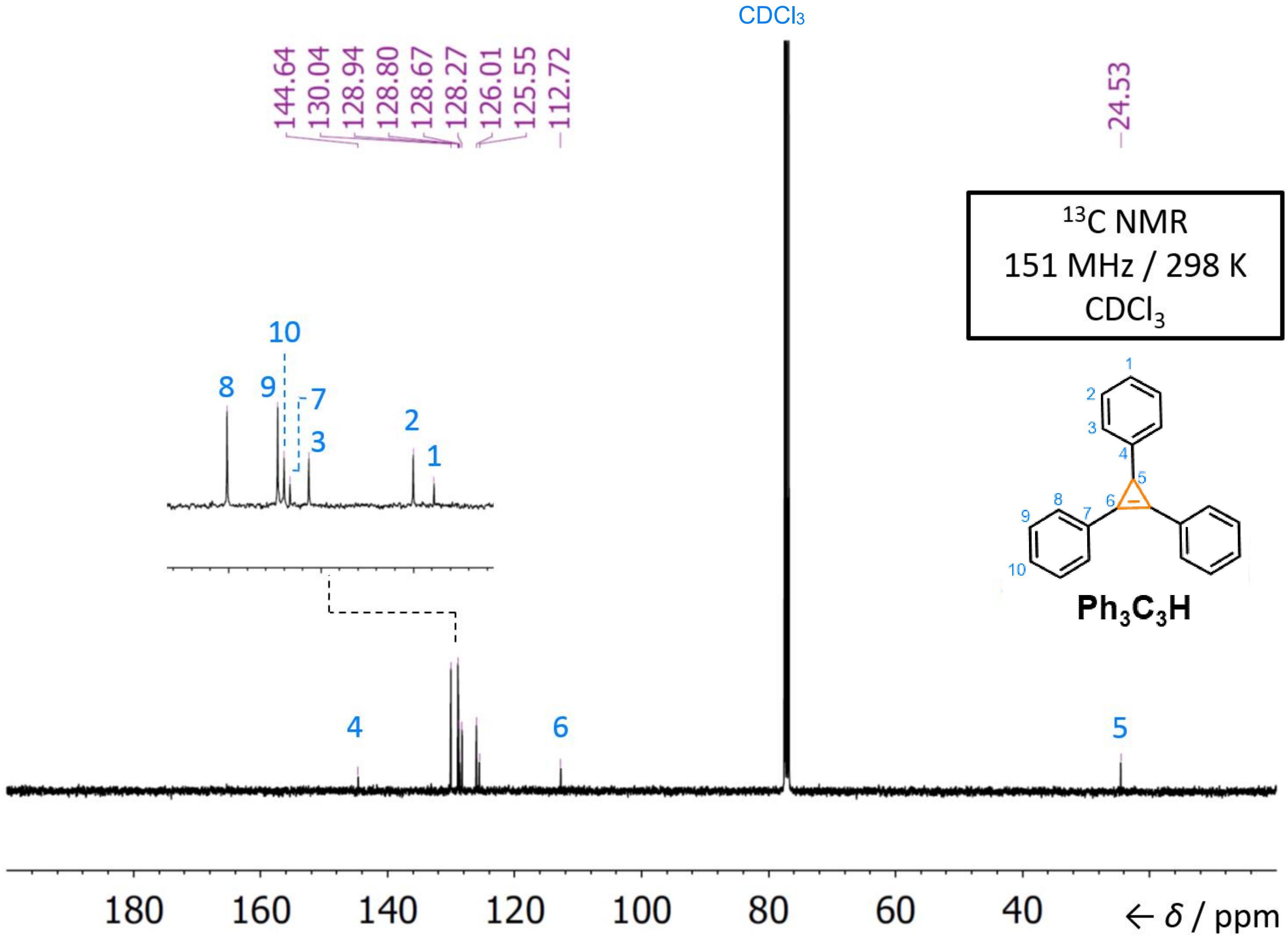

Figure S4. ^{13}C NMR Spectrum of **Ph_3C_3H**.

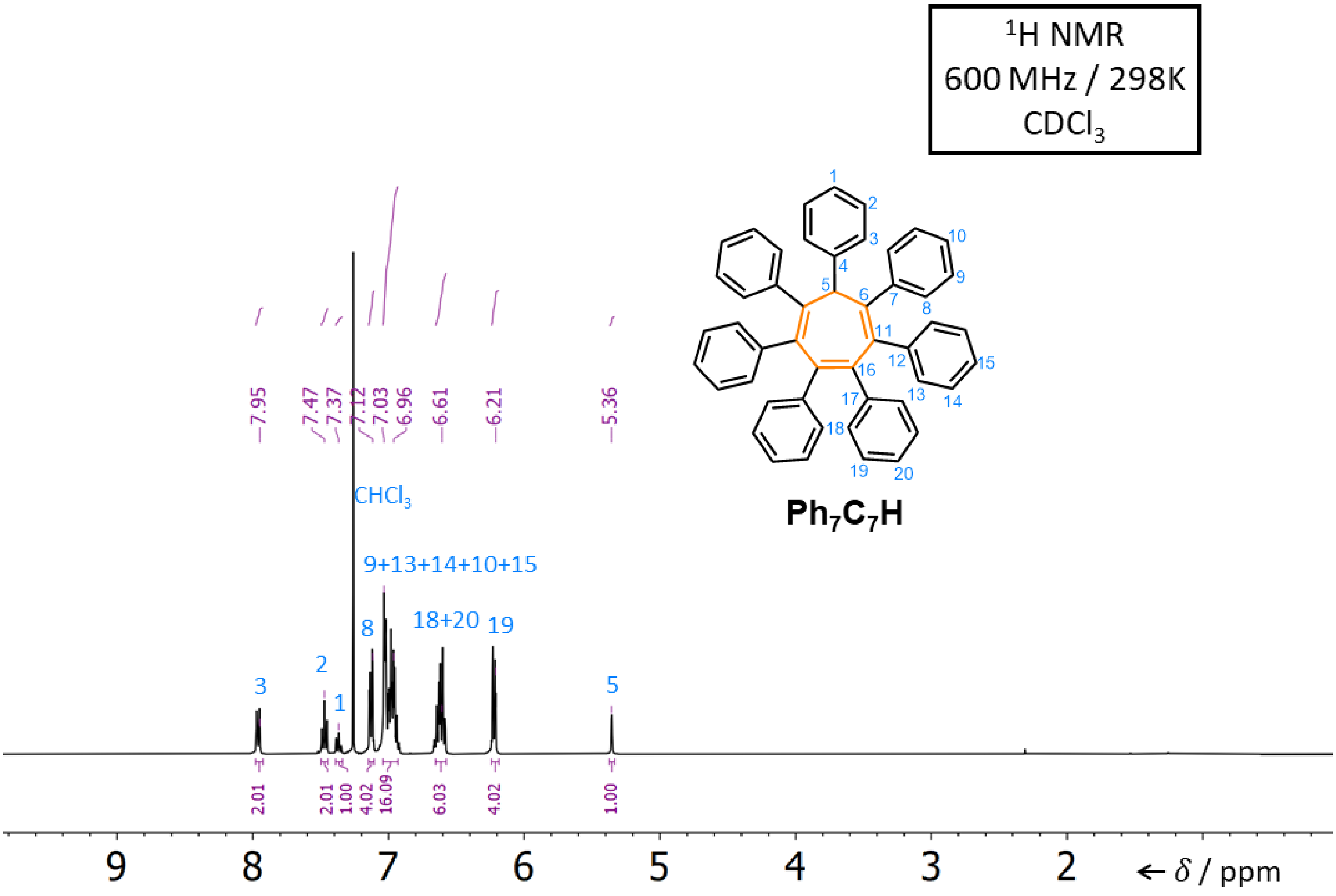

Figure S5. ^1H NMR Spectrum of **Ph_7C_7H**.

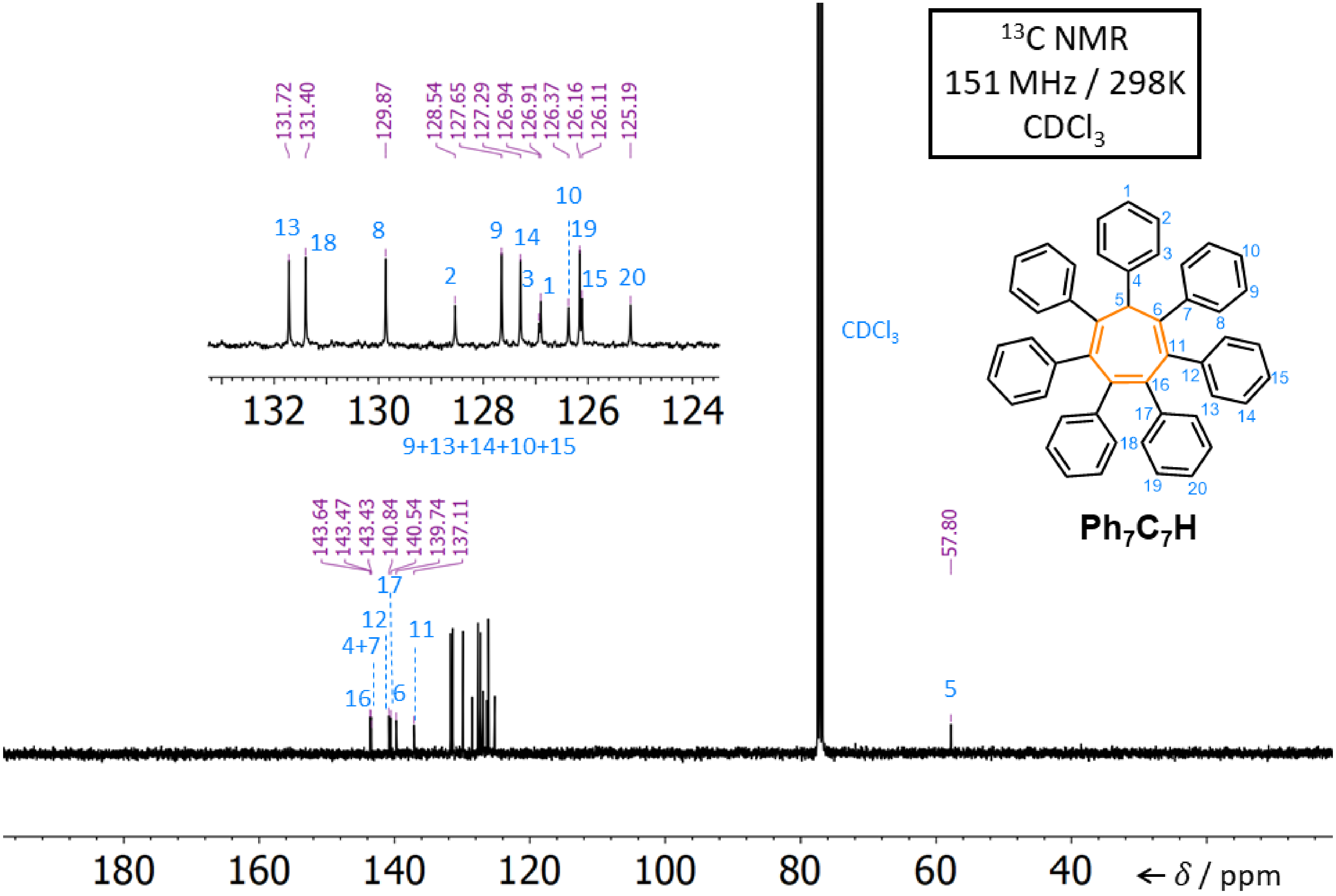

Figure S6. ^{13}C NMR Spectrum of **Ph$_7$C$_7$H**.

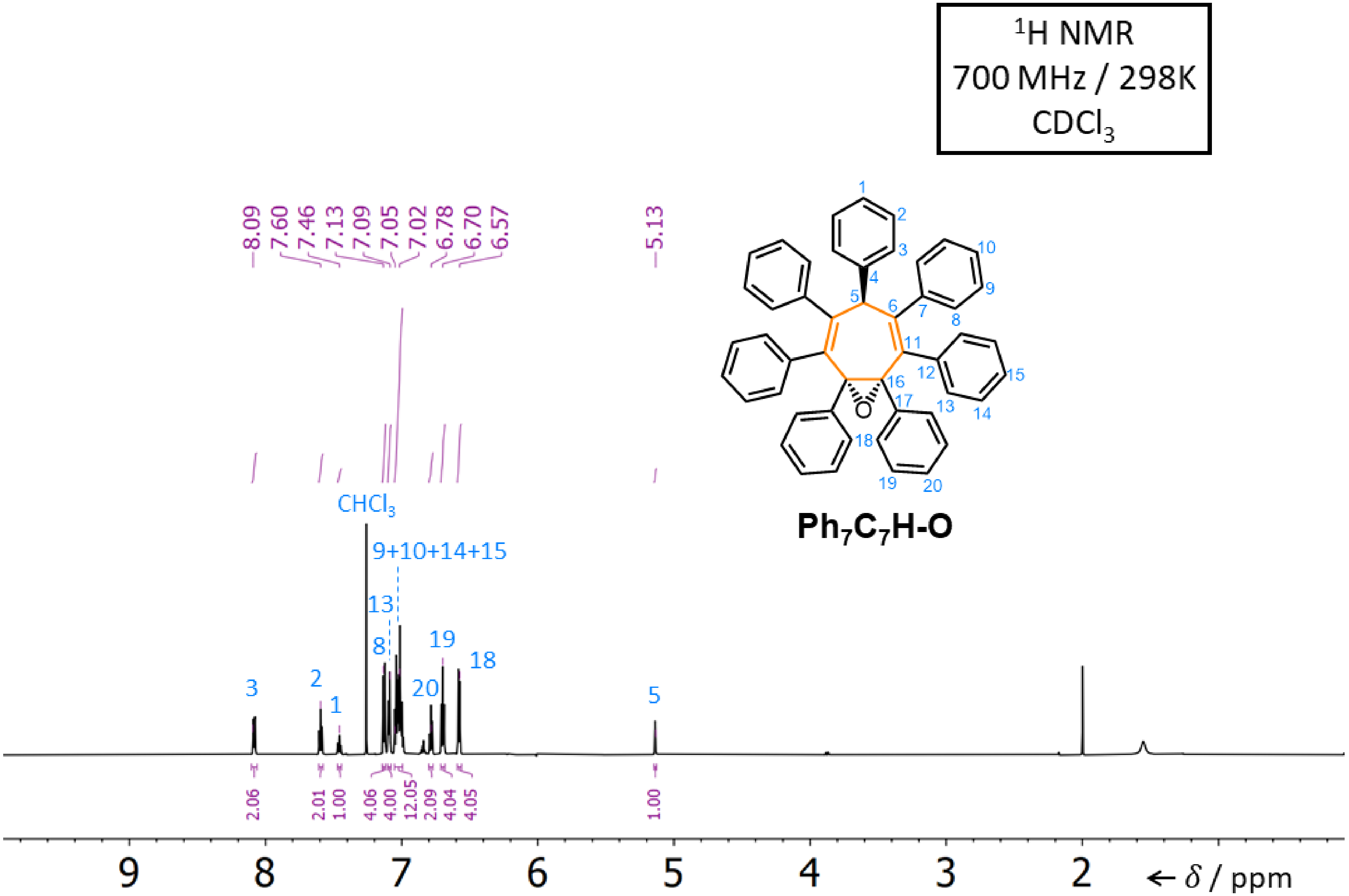

Figure S7. ^{1}H NMR Spectrum of **Ph_7C_7H-O**.

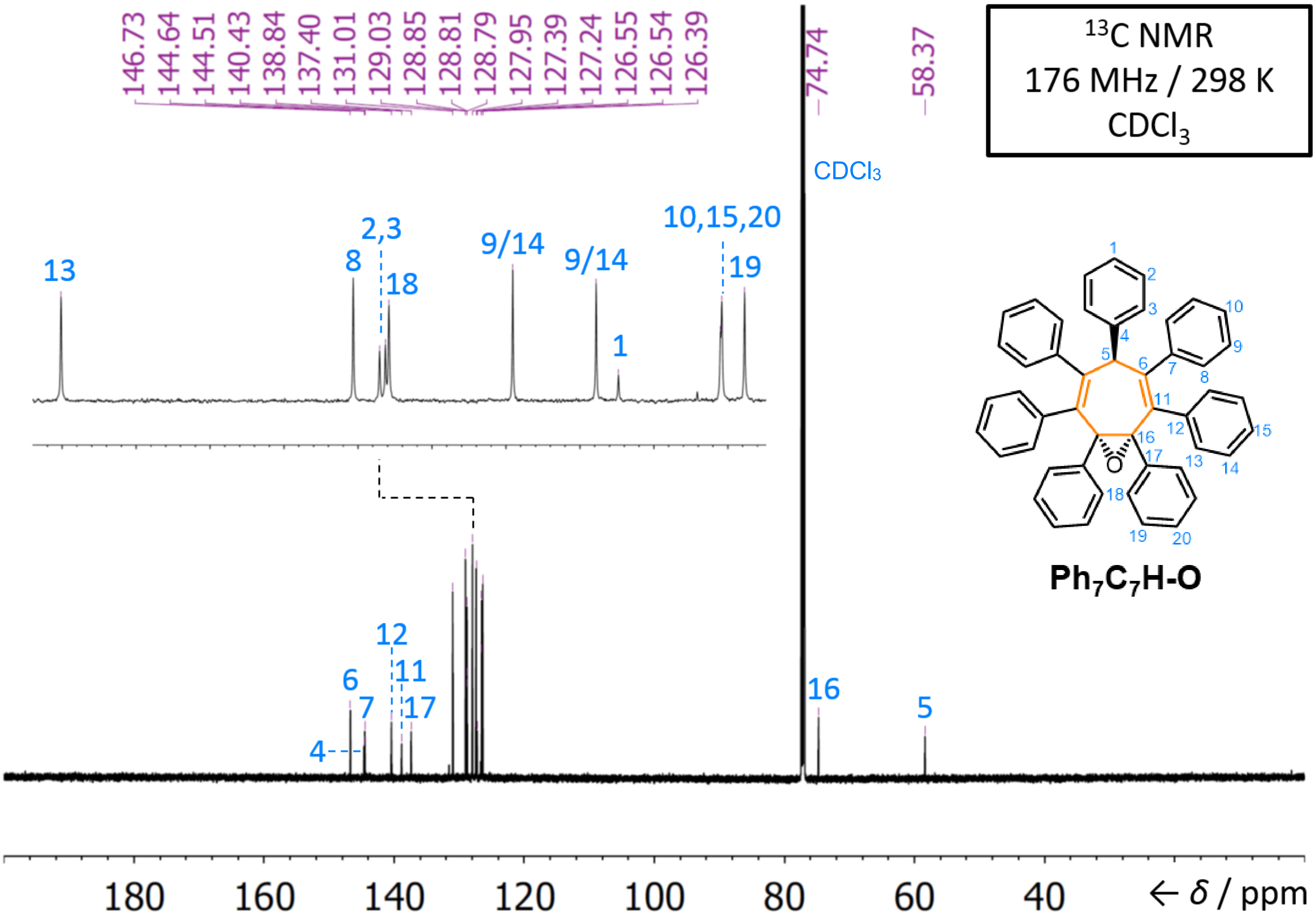

Figure S8. ^{13}C NMR Spectrum of **Ph_7C_7H-O**.

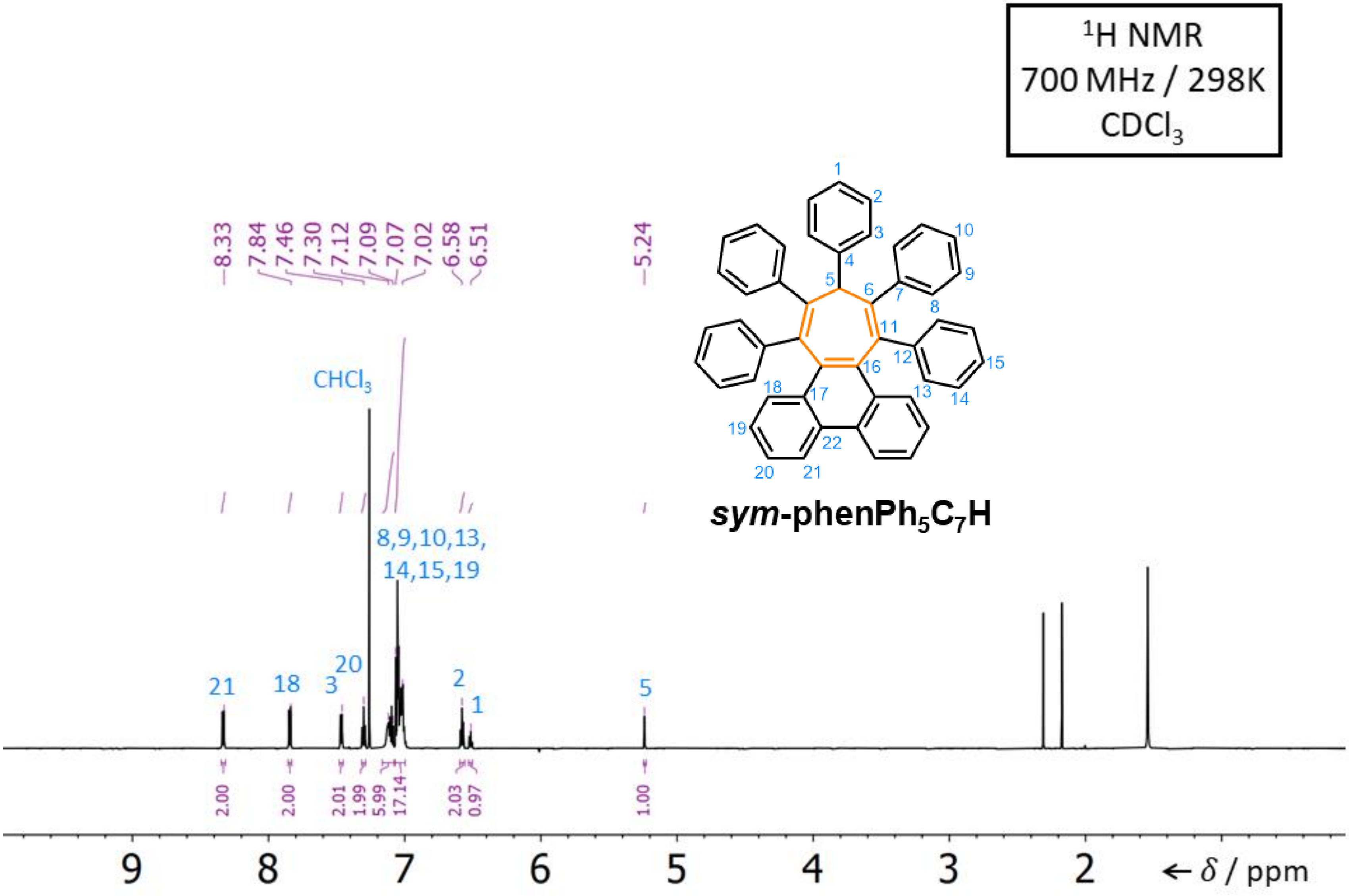

Figure S9. ^{1}H NMR Spectrum of ***sym*-phenPh$_5$C$_7$H**.

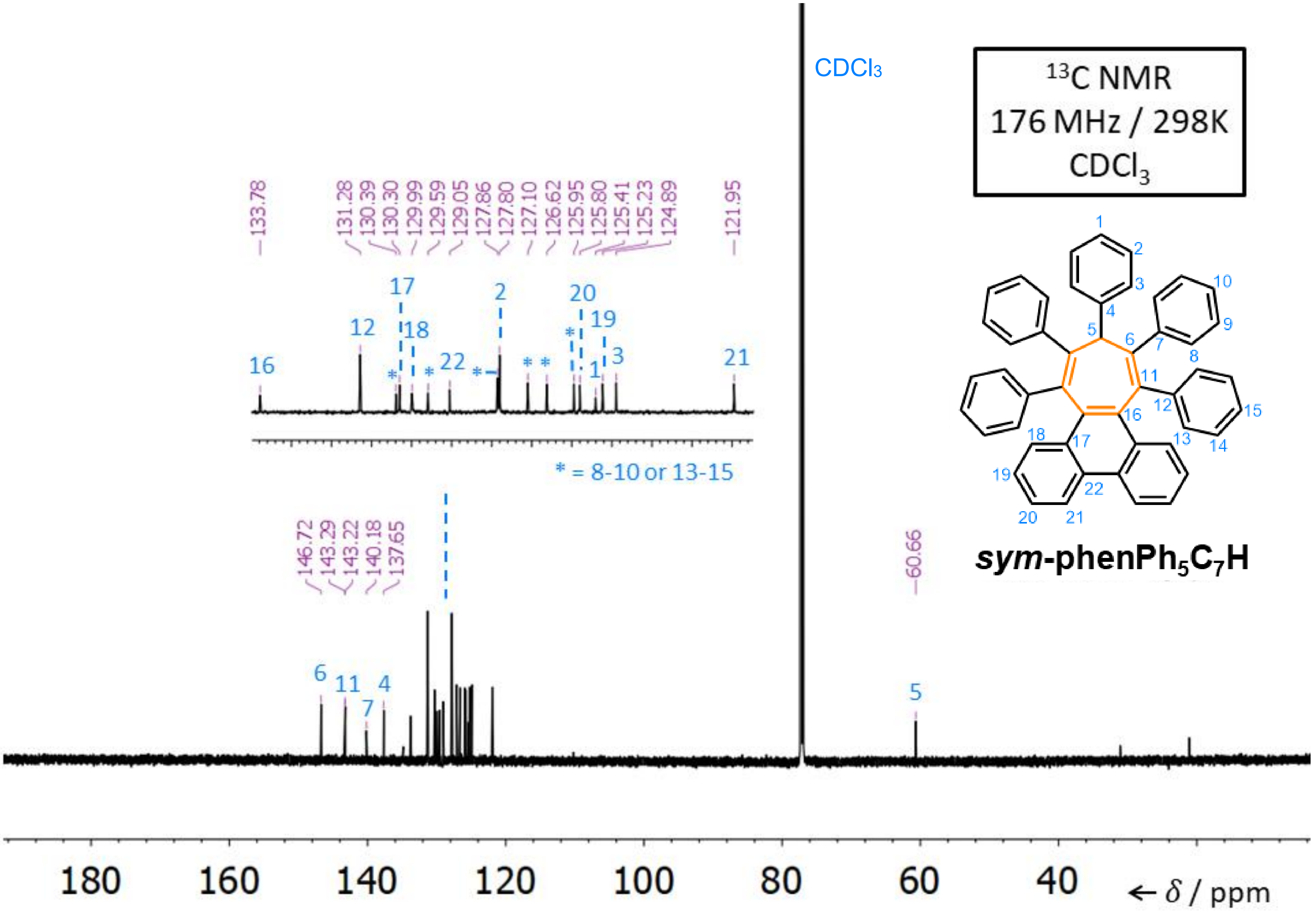

Figure S10. ^{13}C NMR Spectrum of ***sym*-phenPh$_5$C$_7$H**.

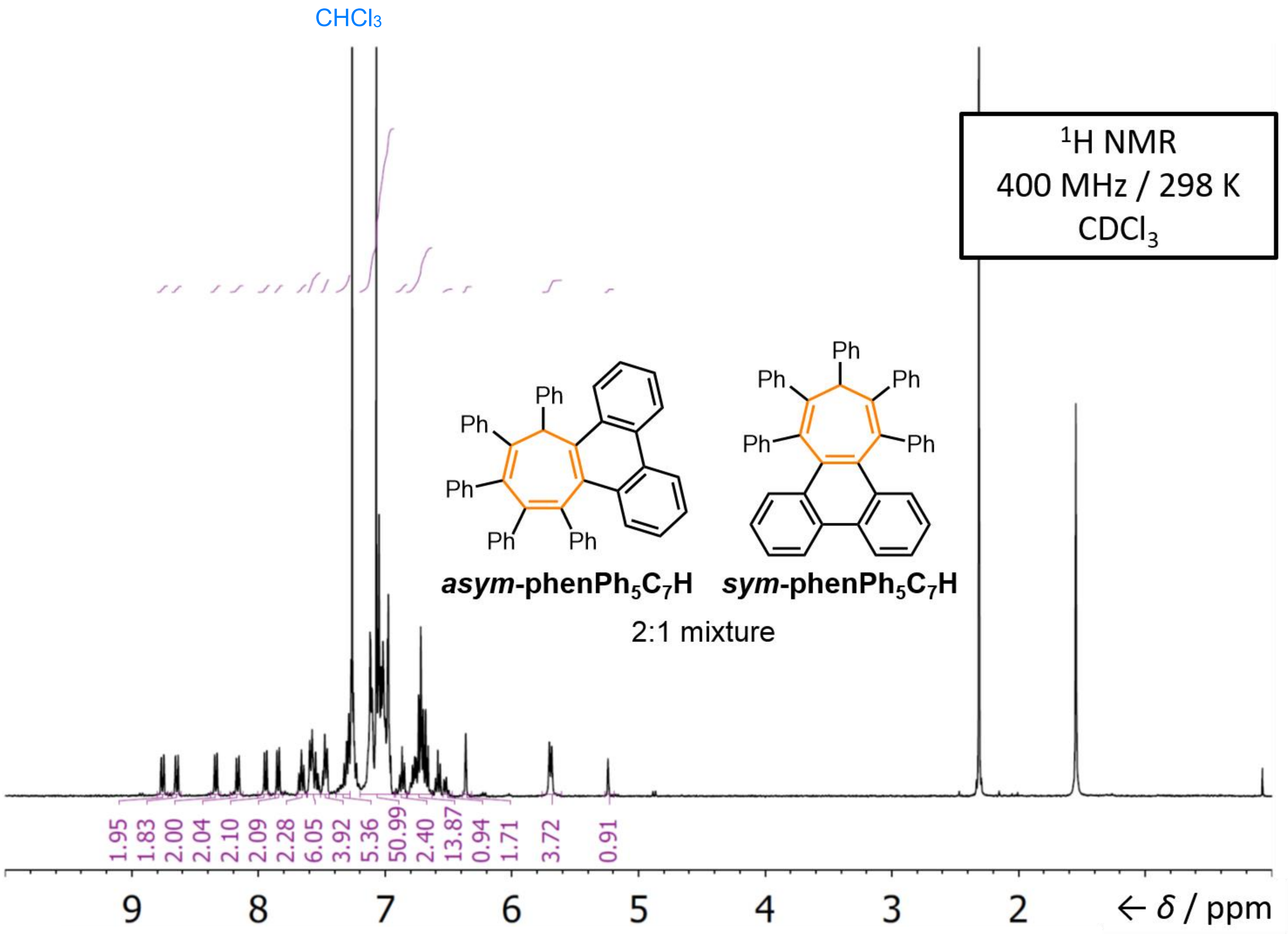

Figure S11. ^{1}H NMR Spectrum of a 2:1 mixture of ***asym*-phenPh$_5$C$_7$H** and ***sym*-phenPh$_5$C$_7$H**.

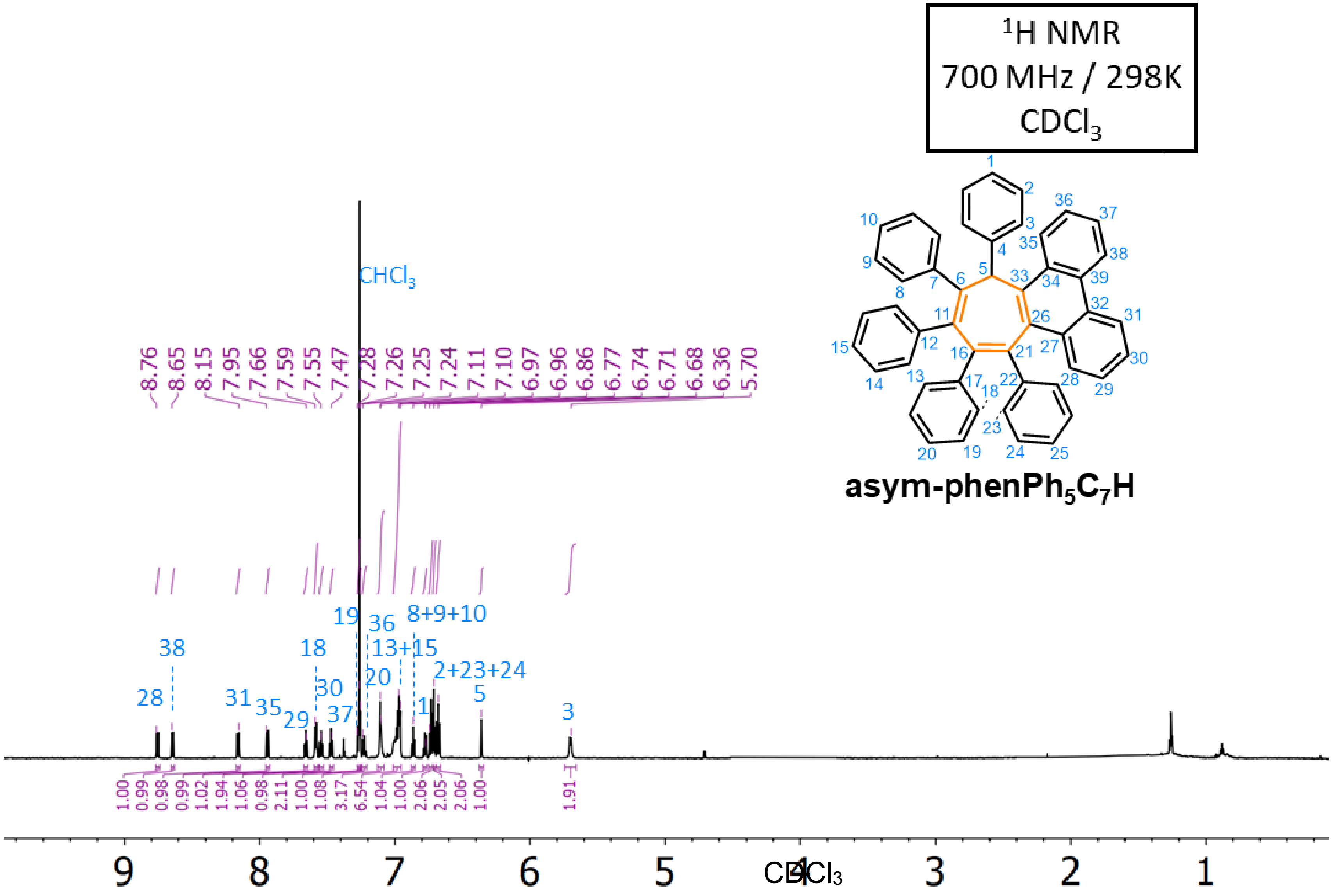

Figure S12. ^{1}H NMR Spectrum of ***asym*-phenPh$_5$C$_7$H**.

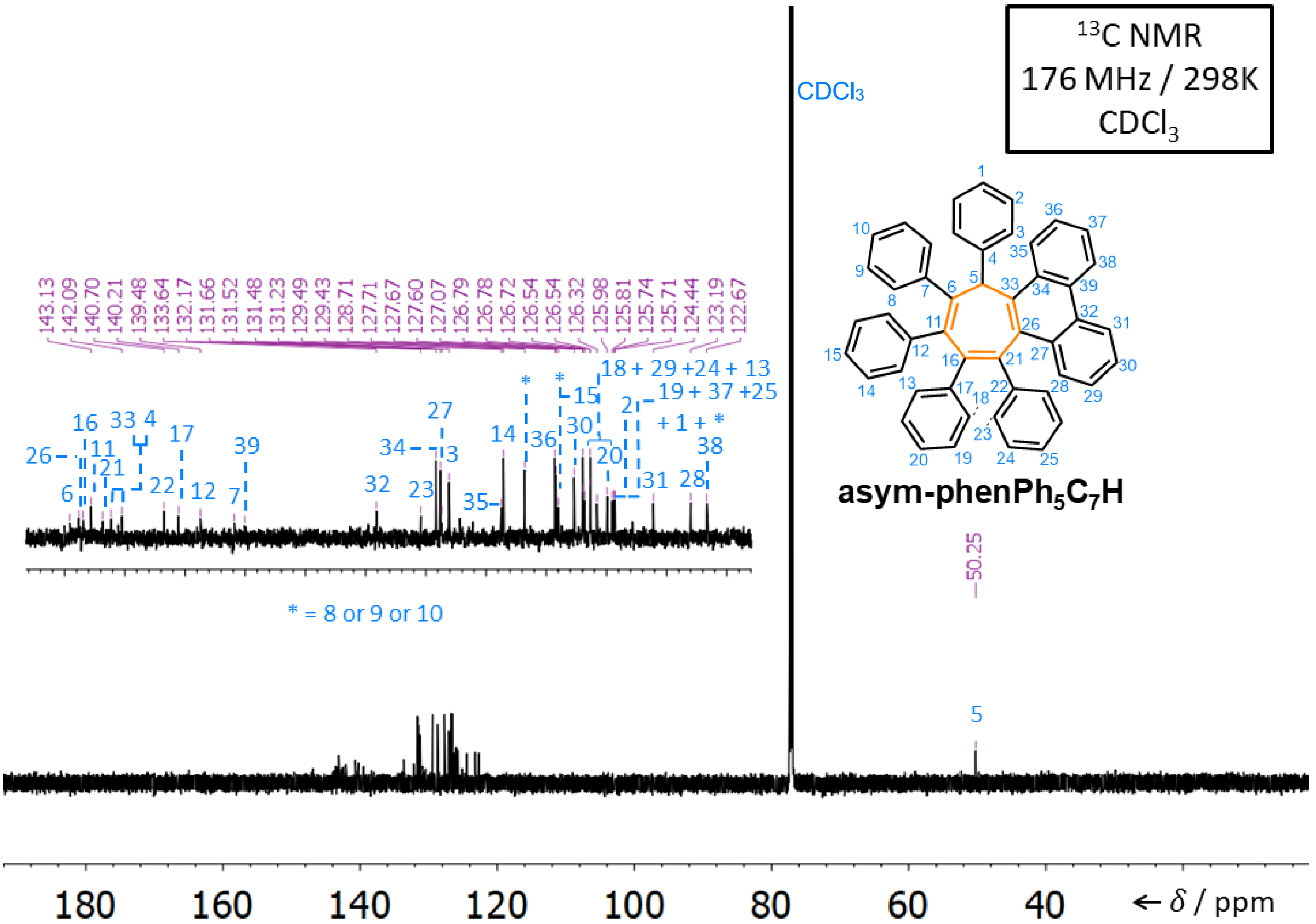

Figure S13. ^{13}C NMR Spectrum of ***asym*-phenPh$_5$C$_7$H**.

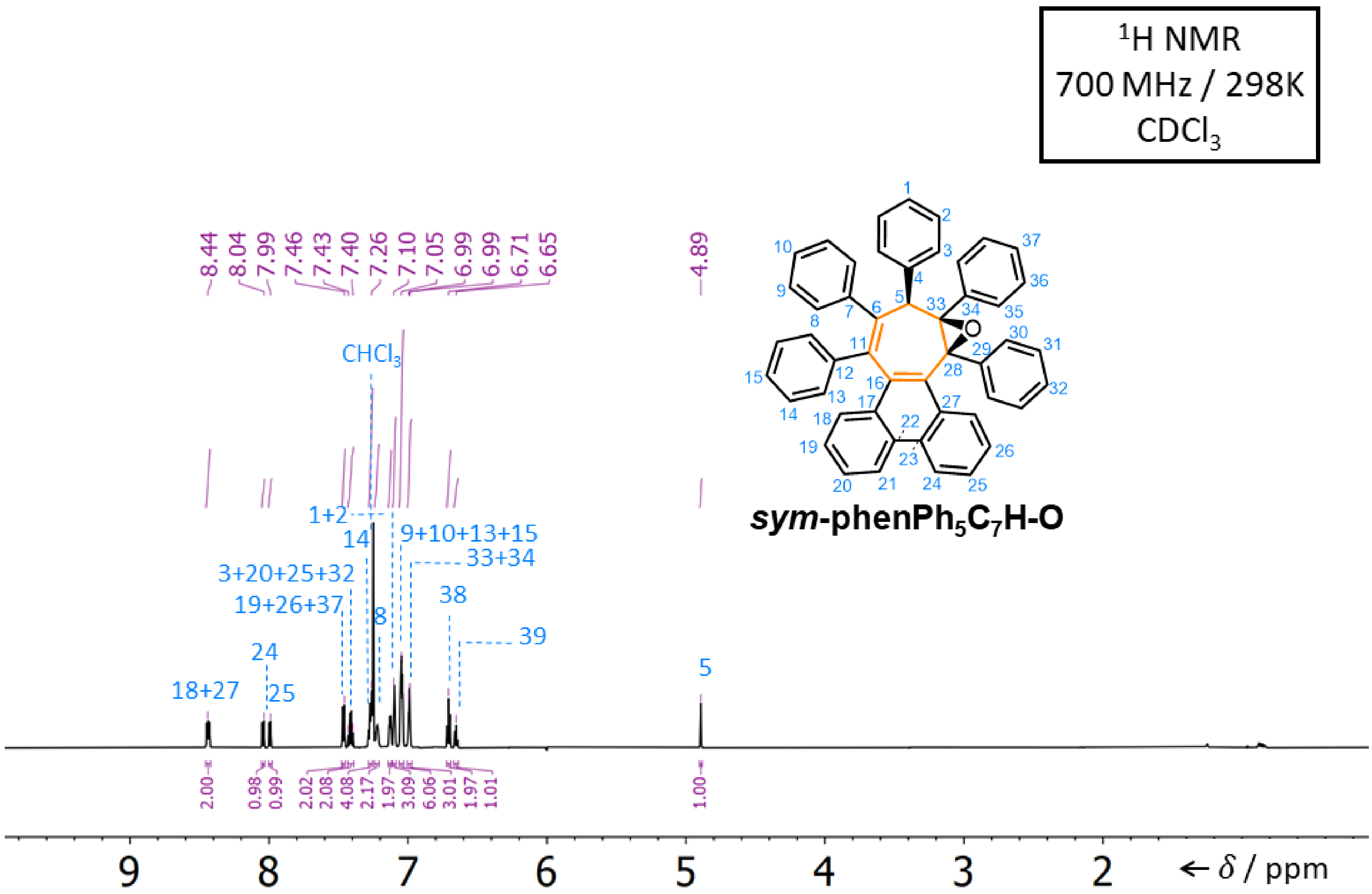

Figure S14. ^{1}H NMR Spectrum of ***sym*-phenPh$_5$C$_7$H-O**.

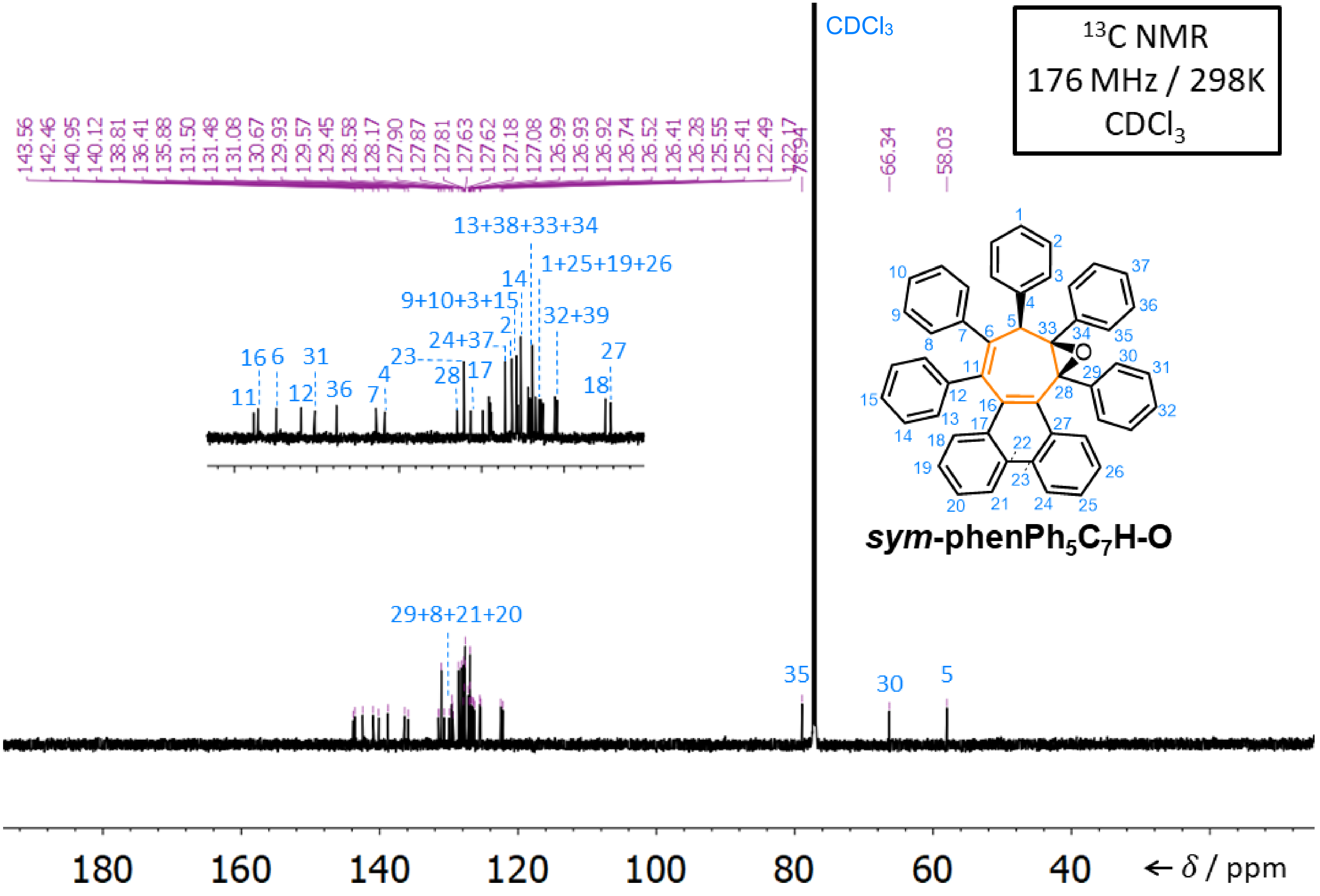

Figure S15. ^{13}C NMR Spectrum of ***sym*-phenPh$_5$C$_7$H-O**.

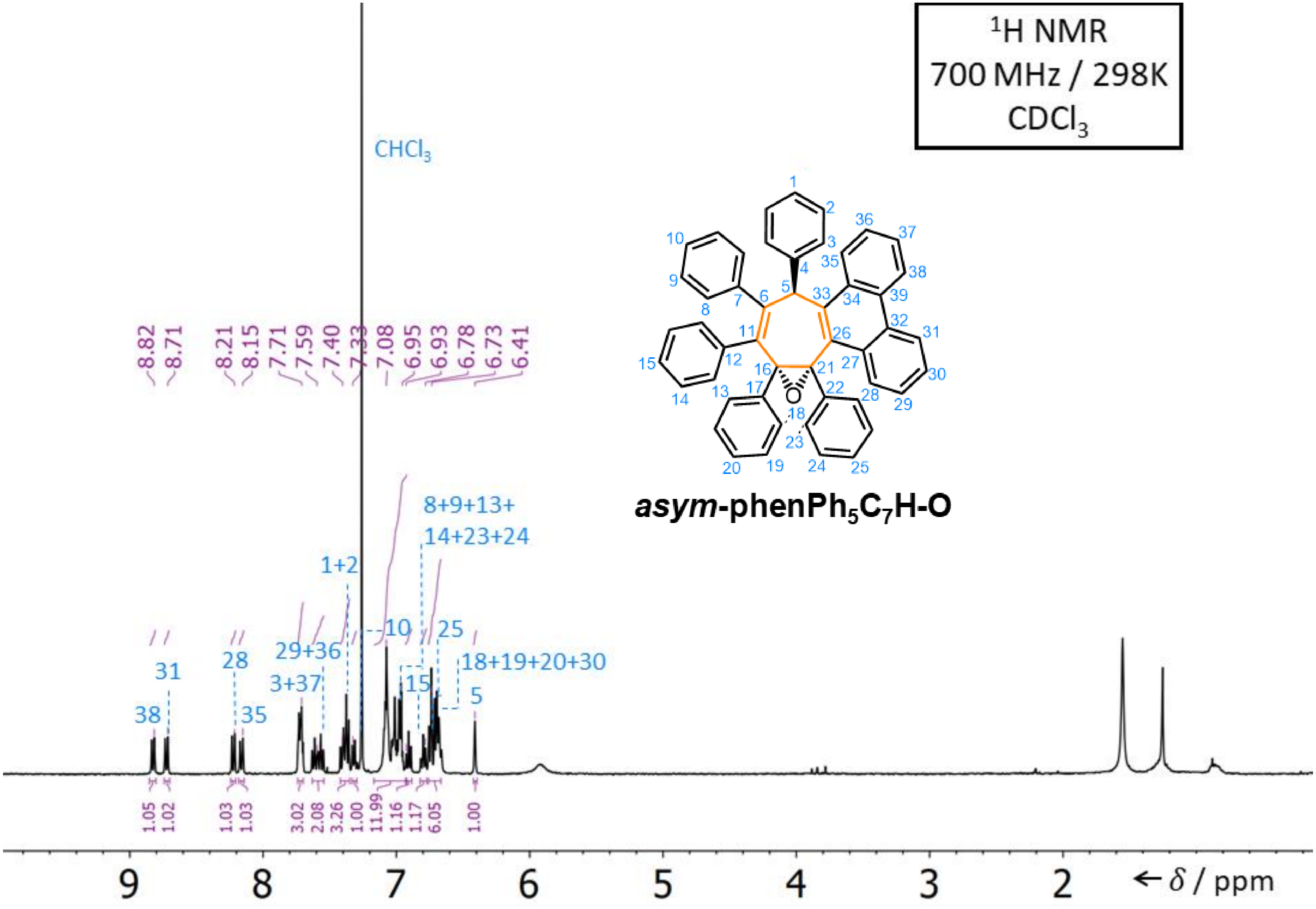

Figure S16. ^{1}H NMR Spectrum of ***asym*-phenPh$_5$C$_7$H-O**.

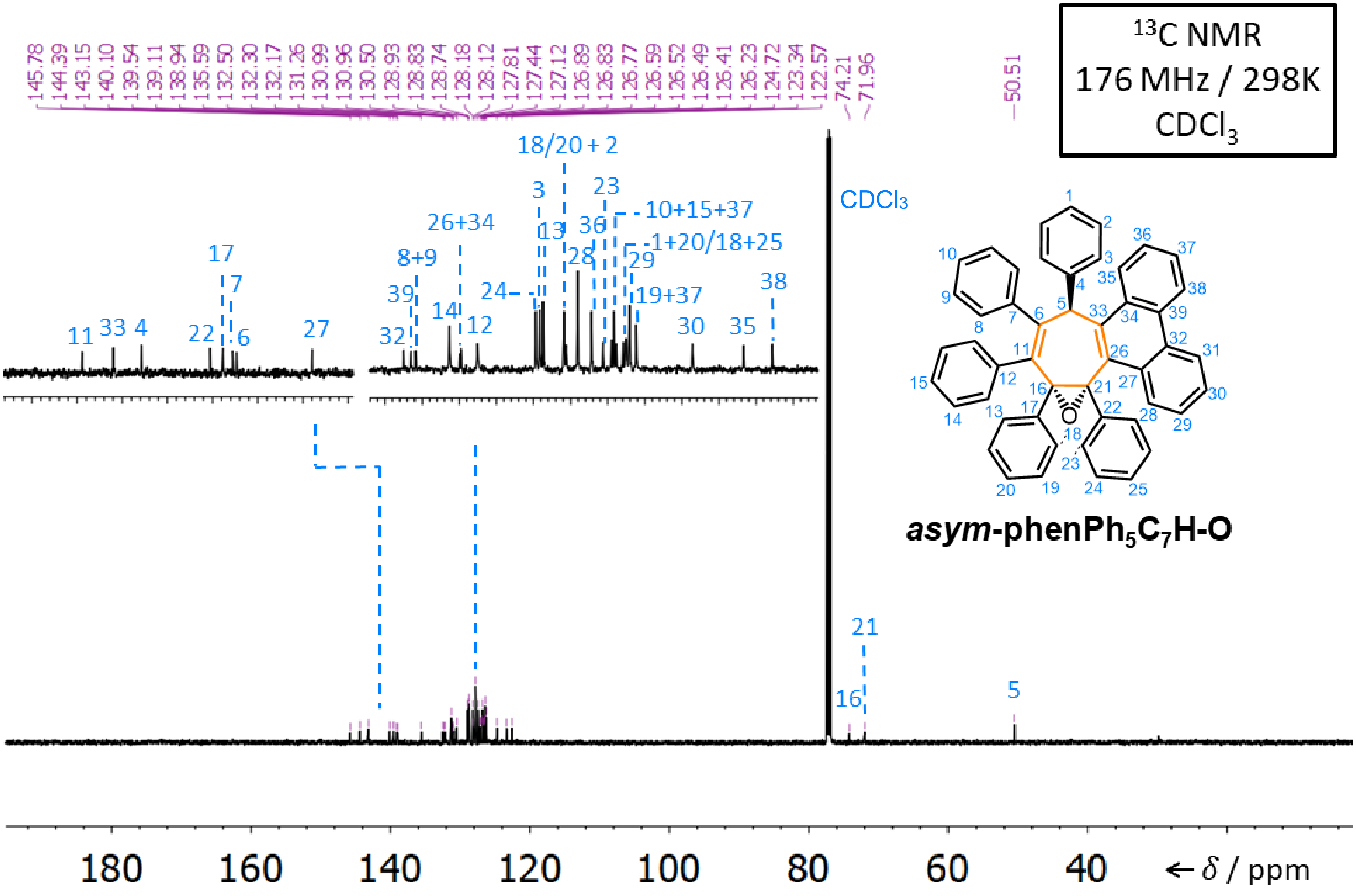

^{13}C NMR
176 MHz / 298K
$CDCl_3$
145.78 144.39 143.15 140.10 139.54 139.11 138.94 135.59 132.50 132.30 132.17 131.26 130.99 130.96 130.50 128.93 128.83 128.74 128.18 128.12 127.81 127.44 127.12 126.89 126.83 126.77 126.59 126.52 126.49 126.41 126.23 124.72 123.34 122.57
74.21
71.96
50.51
18/20 + 2
3
23
10+15+37
13
36
1+20/18+25
26+34
28
29
8+9
24
19+37
17
39
7
38
14
11
33
4
22
6
27
32
12
30
35
$CDCl_3$
21
16
5
180
160
140
120
100
80
60
40
← δ / ppm
asym-phenPh$_5$C$_7$H-O

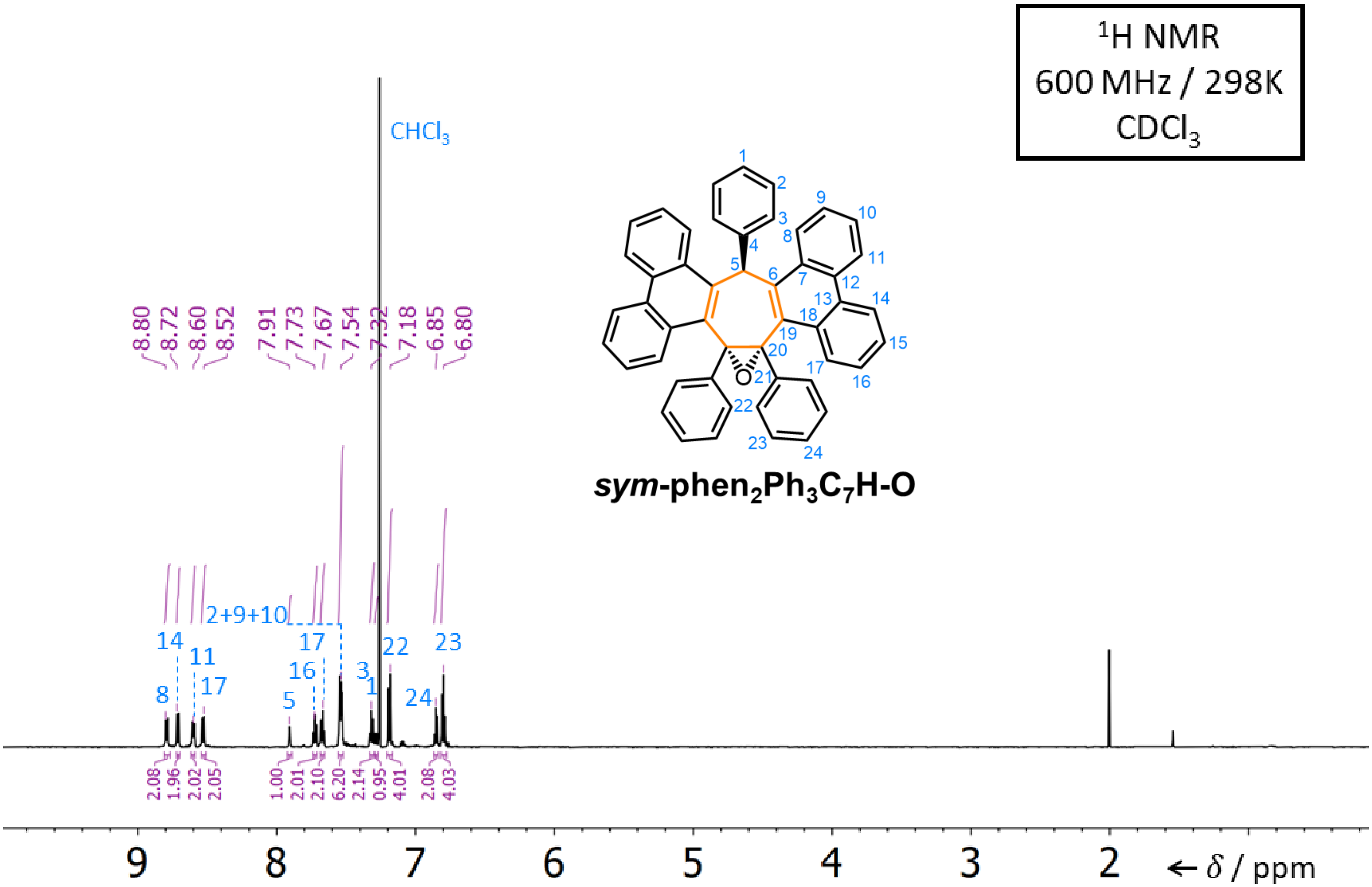

Figure S18. ^{1}H NMR Spectrum of ***sym*-phen$_2$Ph$_3$C$_7$H-O**.

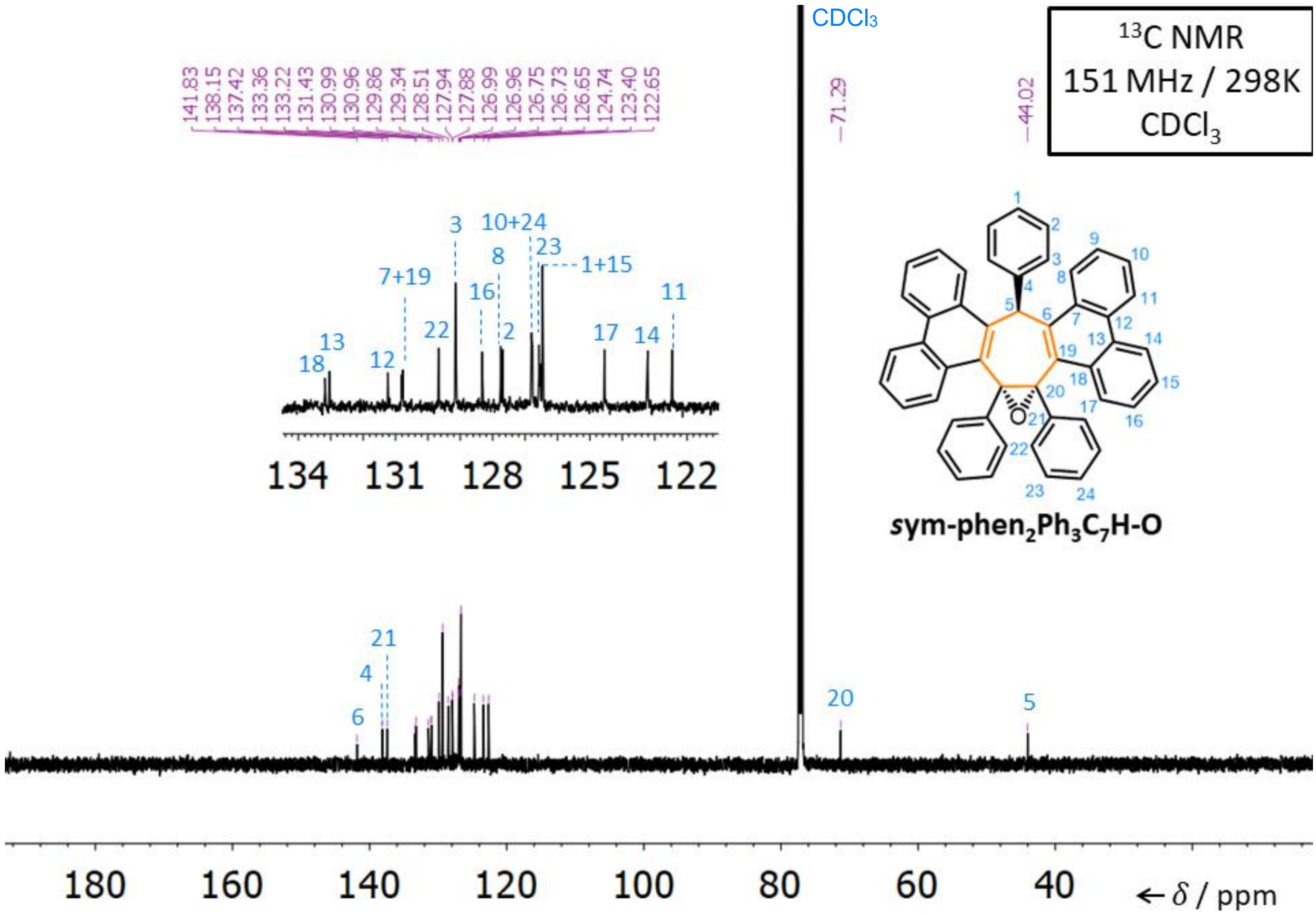

Figure S19. ^{13}C NMR Spectrum of ***sym*-phen$_2$Ph$_3$C$_7$H-O**.

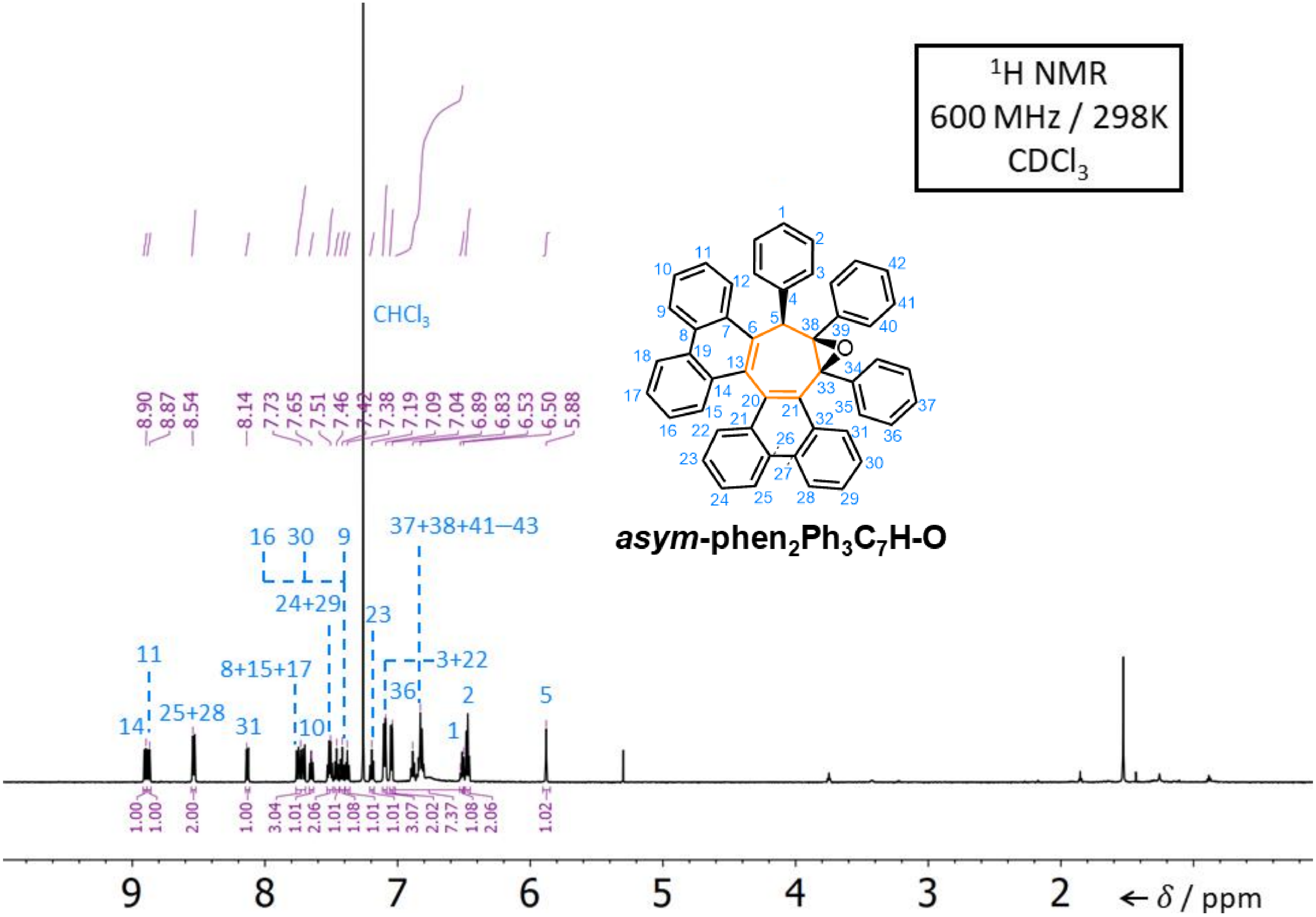

Figure S20. ^{1}H NMR Spectrum of ***asym*-phen$_2$Ph$_3$C$_7$H-O**.

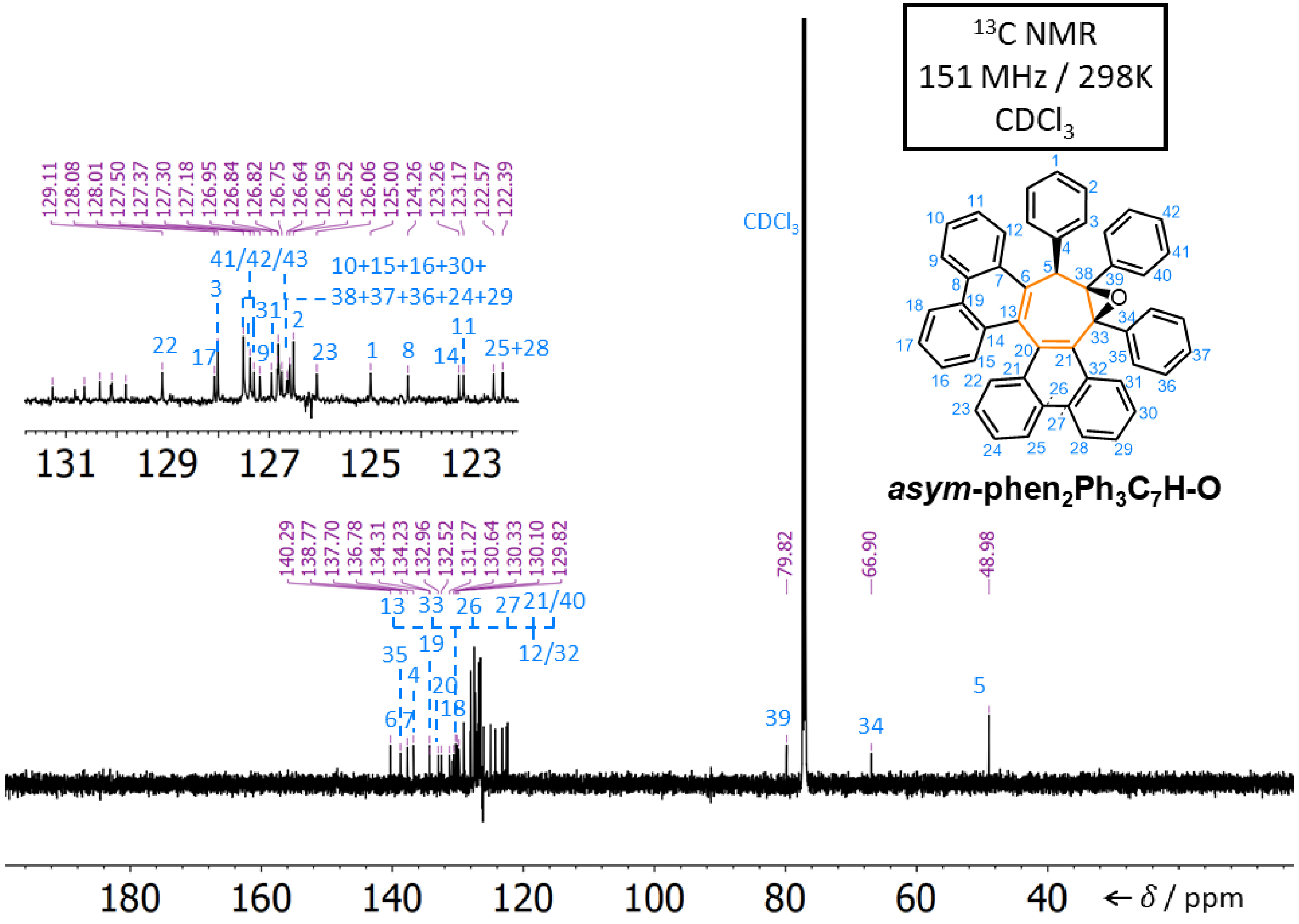

Figure S21. [12]C NMR Spectrum of ***asym*-phen$_2$Ph$_3$C$_7$H-O**

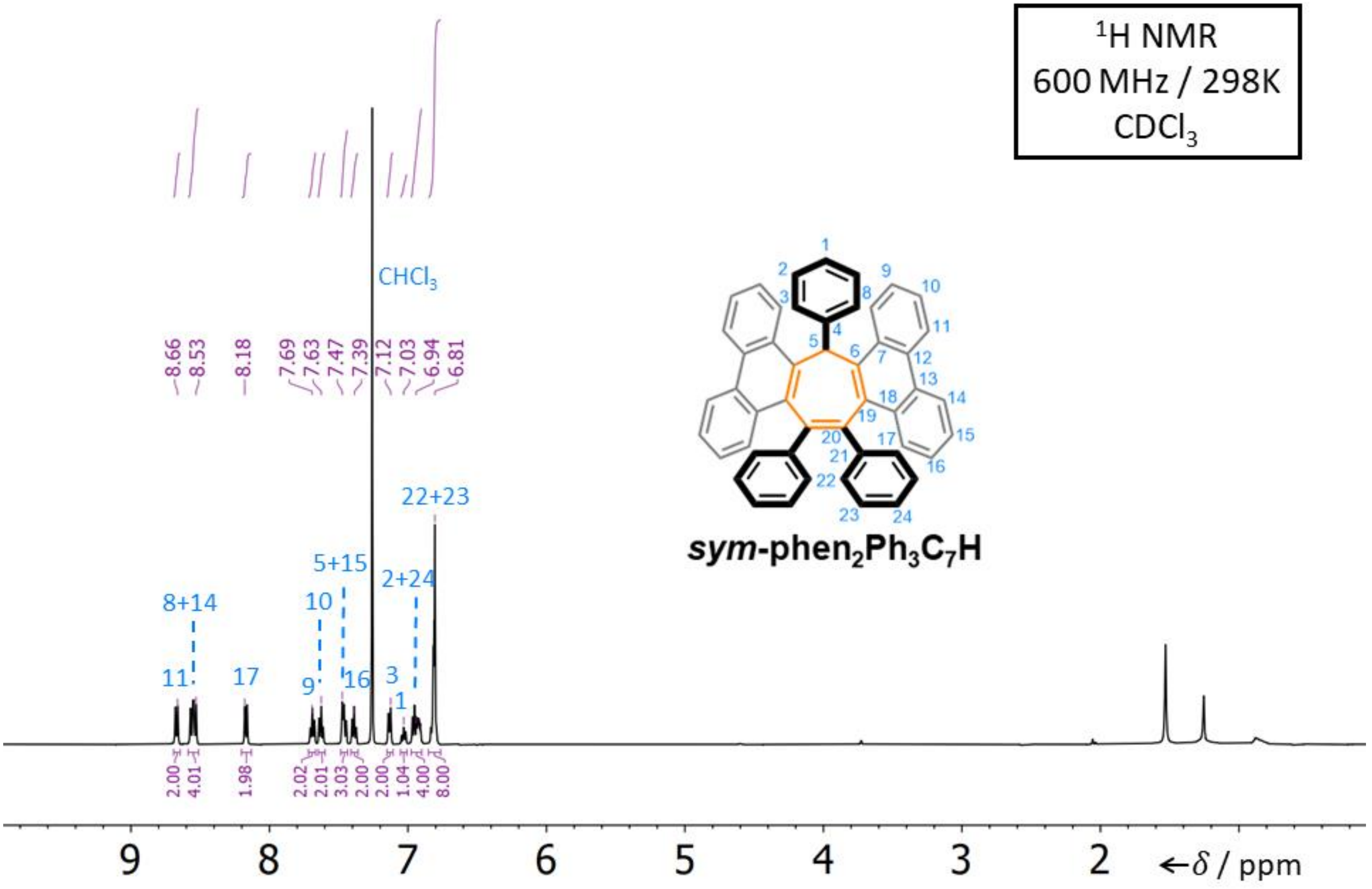

Figure S22. ^{1}H NMR Spectrum of ***sym*-Phen$_2$Ph$_3$C$_7$H**.

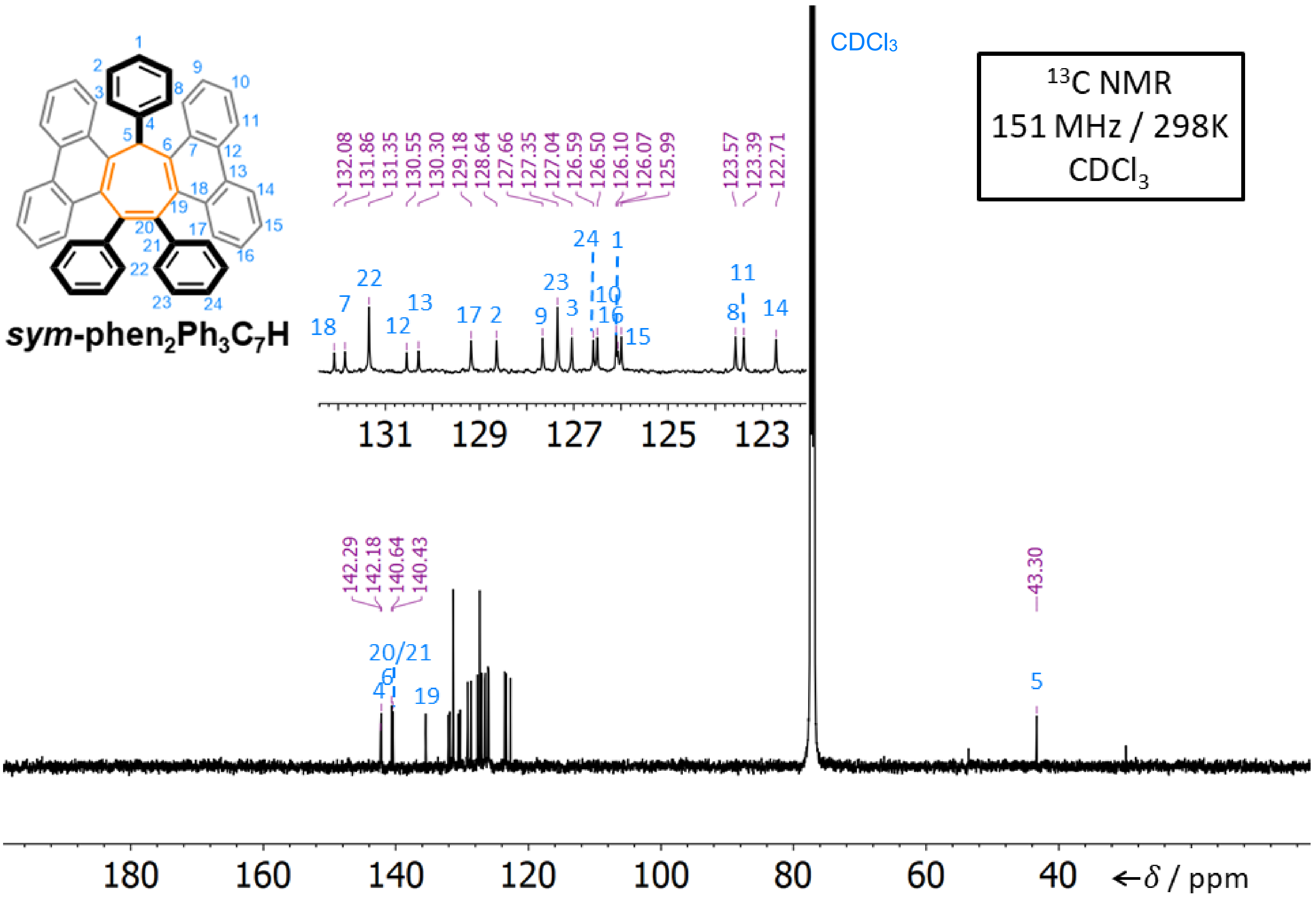

Figure S23. ^{13}C NMR Spectrum of ***sym*-Phen$_2$Ph$_3$C$_7$H**.

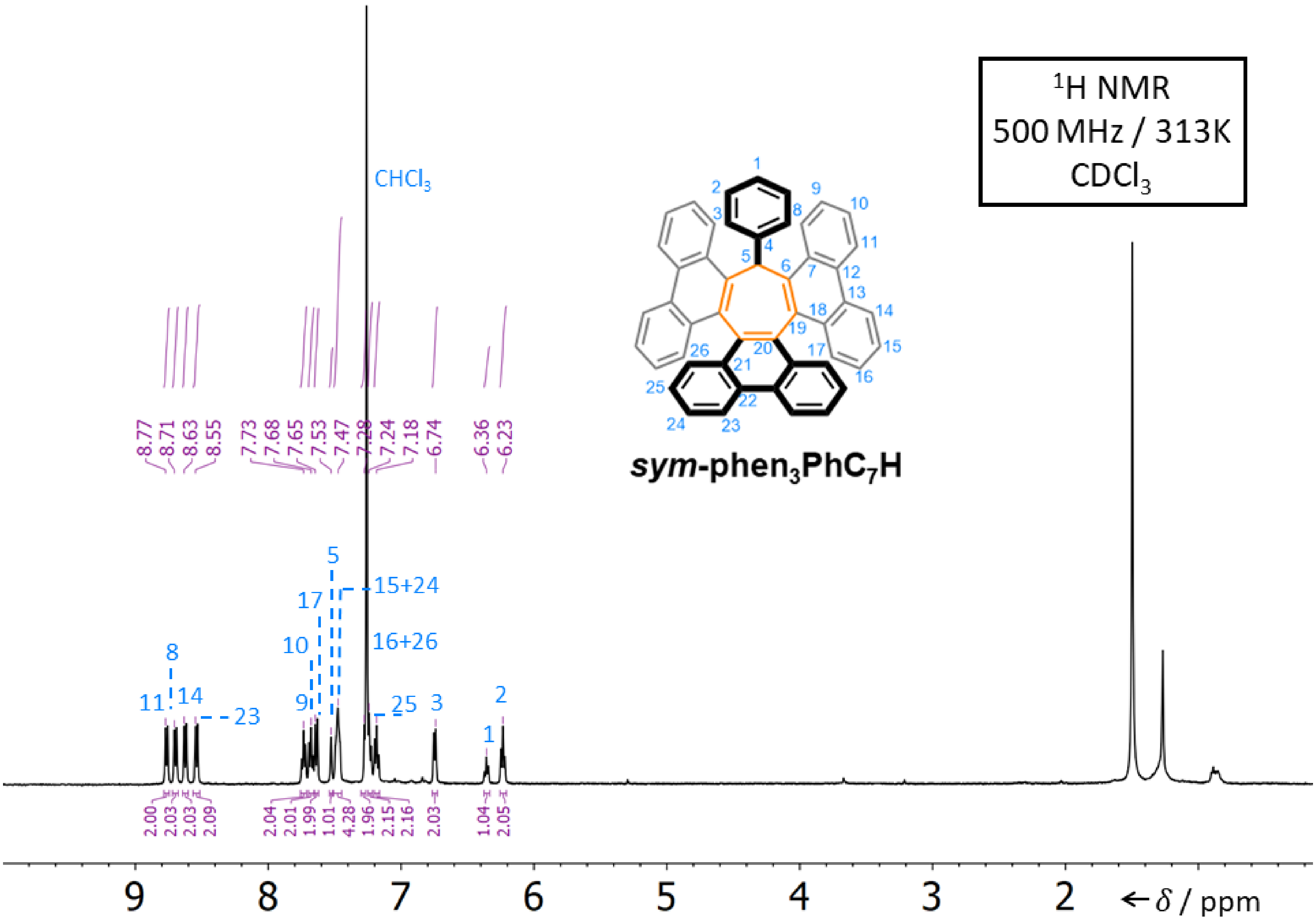

Figure S24. ^{1}H NMR Spectrum of ***sym*-phen$_3$PhC$_7$H**

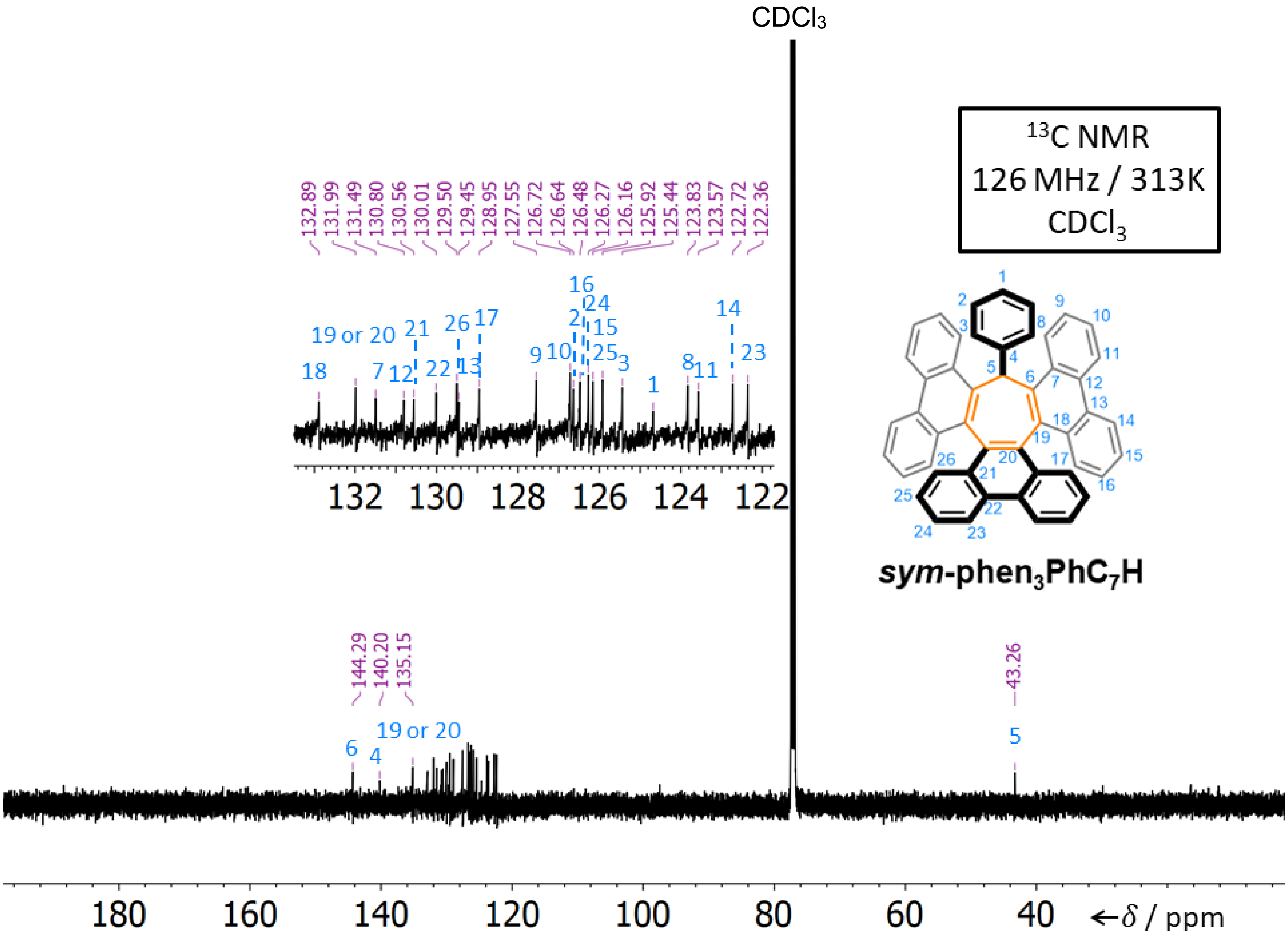

Figure S25. ^{13}C NMR Spectrum of ***sym*-phen$_3$PhC$_7$H**

3.1. Variable-Temperature (VT) NMR Spectroscopy

We performed VT NMR measurements to determine the energy barrier to rotation of phenyl rings in ***sym*-phenPh$_5$C$_7$H**. As we were not able to unambiguously assign all ^{13}C resonances based on 2D NMR spectra, we have assigned (Figure S25) the phenyl group that experiences the highest energy barrier to rotation as ring c by analogy to our investigation of **Ph$_7$C$_7$H** reported previously.[3] VT ^{13}C NMR spectra were acquired to facilitate analysis using a two-spin system model in the WinDNMR[4] software package. In order to obtain a solution of ***sym*-phenPh$_5$C$_7$H** with a low freezing point and of sufficiently high concentration (20 mg in 0.8 mL) for ^{13}C NMR analysis, a mixture of CS_2 (0.7 mL) with CD_2Cl_2 (0.1 mL) was used as solvent. A series of spectra ranging from 24 to –98 °C were recorded.

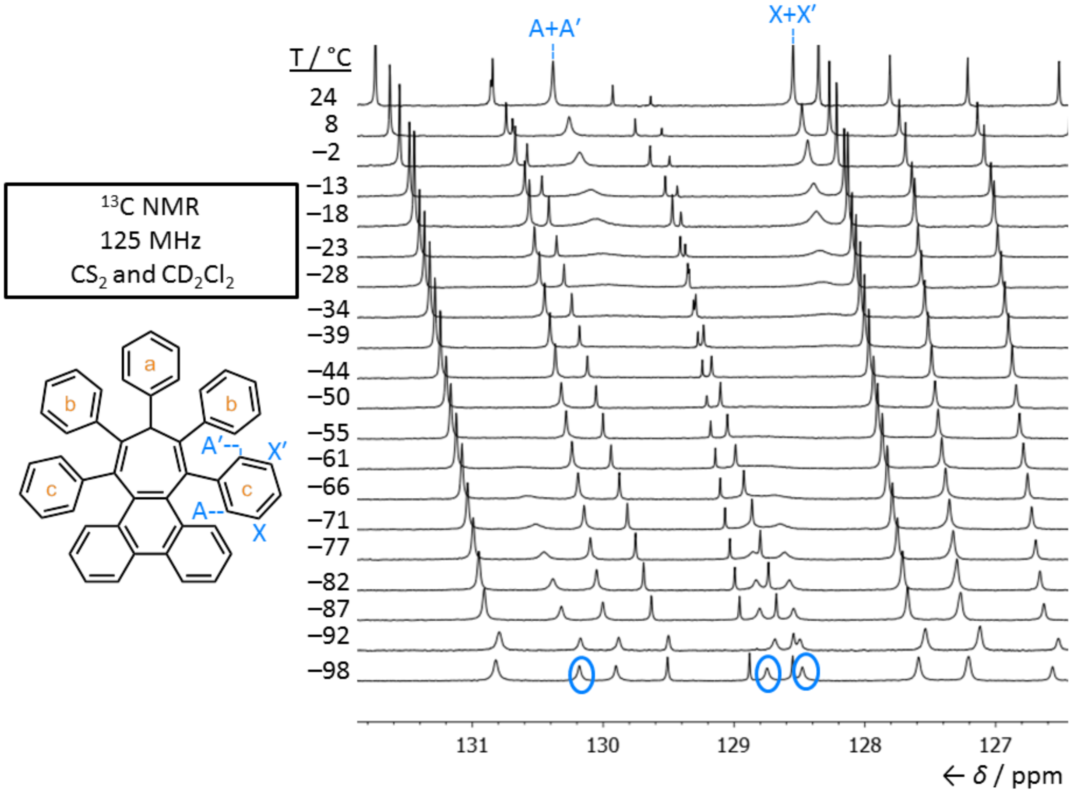

Figure S26. Partial ^{13}C VT NMR spectra of ***sym*-phenPh$_5$C$_7$H**. Peaks corresponding to the carbon pairs A/A′ and X/X′ are observed as individual, averaged signals in the fast exchange regime, but appear as distinct signals at low temperature in the slow exchange regime. Only three of the four signals can be distinguished at low temperature on account of overlapping signals; however, only one pair is needed to perform lineshape analysis.

Analysis of the ^{13}C spectra reveals that within the temperature range studied, signals corresponding to ring c carbons broaden, merge into the baseline, and then re-emerge as four separate peaks as the temperature is decreased. There are two pairs of resonances in slow exchange below –60 °C. Only three resonances can be observed as distinct peaks as the fourth overlaps with other signals. In order to determine which of the three re-emerged peaks correspond to a pair of exchanging sites, a HSQC experiment was performed at –98 °C (Figure S27). Both ^{1}H NMR and ^{13}C NMR spectra had been recorded from 24–98 °C.

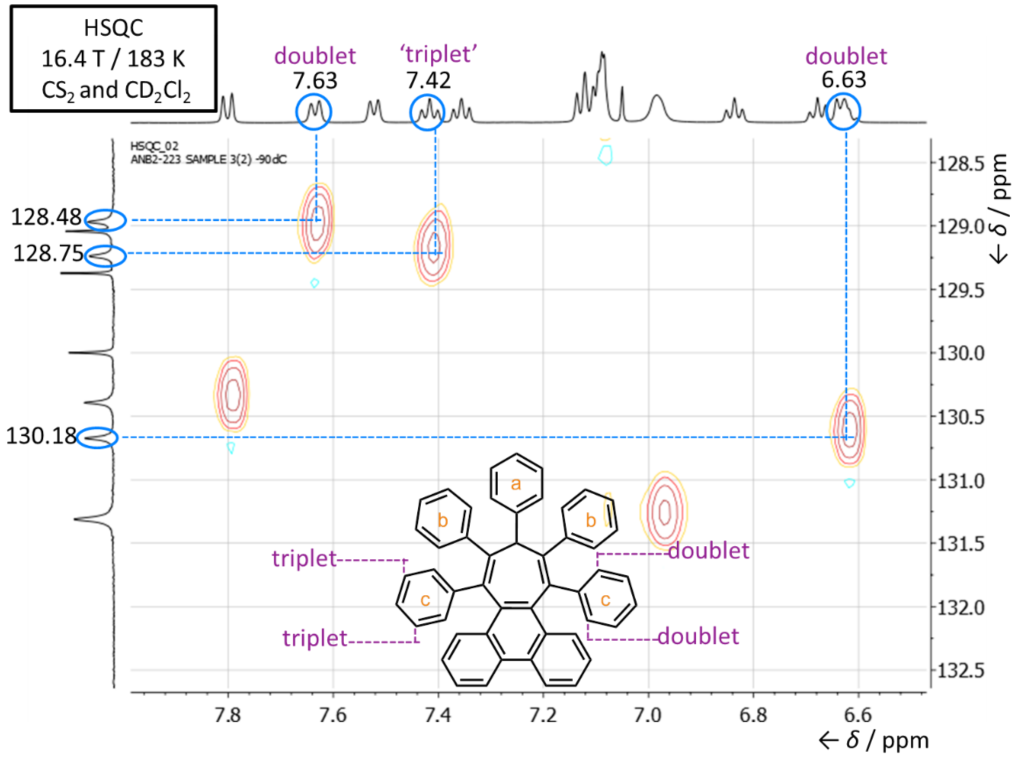

Figure S27. Partial HSQC NMR spectrum of ***sym*-phenPh$_5$C$_7$H** recorded at –98 °C. Correlations are highlighted between the ^{13}C signals observed to be in slow exchange by VT NMR (Figure S16) and the corresponding ^{1}H signals. Inset: based on the *J* coupling patterns, the different positions of ring c can be distinguished.

Analysis of the HSQC spectrum reveals that the three ^{13}C signals that have re-emerged from the baseline correlate with two doublets and a signal that appears as an apparent triplet in the ^{1}H NMR. The signals correlating to the two doublets must be the pair of exchanging nuclear environments.

The doublet at 6.63 ppm looks broad, but this is due to peaks under the doublet and this has been confirmed by integration. Thus, we can assign (Figure S28) the labels A+A′ to these resonances. We selected eight spectra for further analysis, choosing temperatures close to the transition of ring c resonances from fast to the slow exchange regimes. Lineshape analysis was performed to derive rate constants by comparison to model spectra produced using WinDNMR.

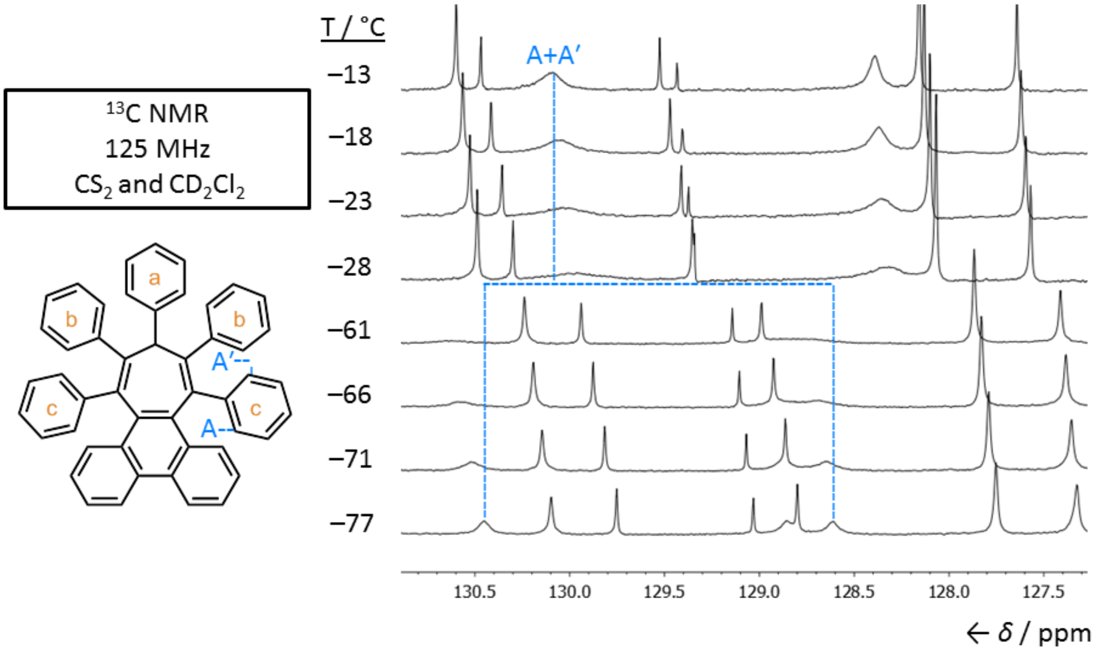

Figure S28. ^{13}C VT NMR spectra of ***sym*-phenPh$_5$C$_7$H** used for lineshape analysis. The dashed blue lines illustrate the resolution of a single peaks into a pair of peaks as temperature decreases.

Activation energy barriers for the rotation of ring c were calculated for each of the eight temperatures using equation S1:

$$\Delta G^{\ddagger} = -RT \ln \frac{k_{\mathrm{r}} h}{k_{\mathrm{B}} T}$$

Equation S1. A variation of the Eyring equation.

Where k_{r} is the measured rate constant, k_{B} is the Boltzmann constant, T is temperature, h is Planck's constant, and R is the ideal gas constant.

Table S1. Calculated activation energies ($\Delta G^{\ddagger}$) for the rotation of ring c of ***sym*-phenPh$_5$C$_7$H**.

Entry	Temperature / K (± 0.64)	$\Delta G^{\ddagger}$ / kJ mol^{-1}
1	260.4	43.3
2	255.1	43.4
3	249.7	43.2
4	244.4	43.0
5	212.5	41.8
6	207.2	42.0
7	201.9	41.8
8	196.6	41.8

A line was fitted (Figure S28) to a plot of $\Delta G^{\ddagger}$ vs T. The slope of the line corresponds to $-\Delta S^{\ddagger}$ and the y-axis intercept to $\Delta H^{\ddagger}$. The entropy of activation $\Delta S^{\ddagger}$ was calculated to be –27.9 J·mol^{-1}·K^{-1} and enthalpy of activation $\Delta H^{\ddagger}$ was calculated to be 36.2 kJ·mol^{-1}.

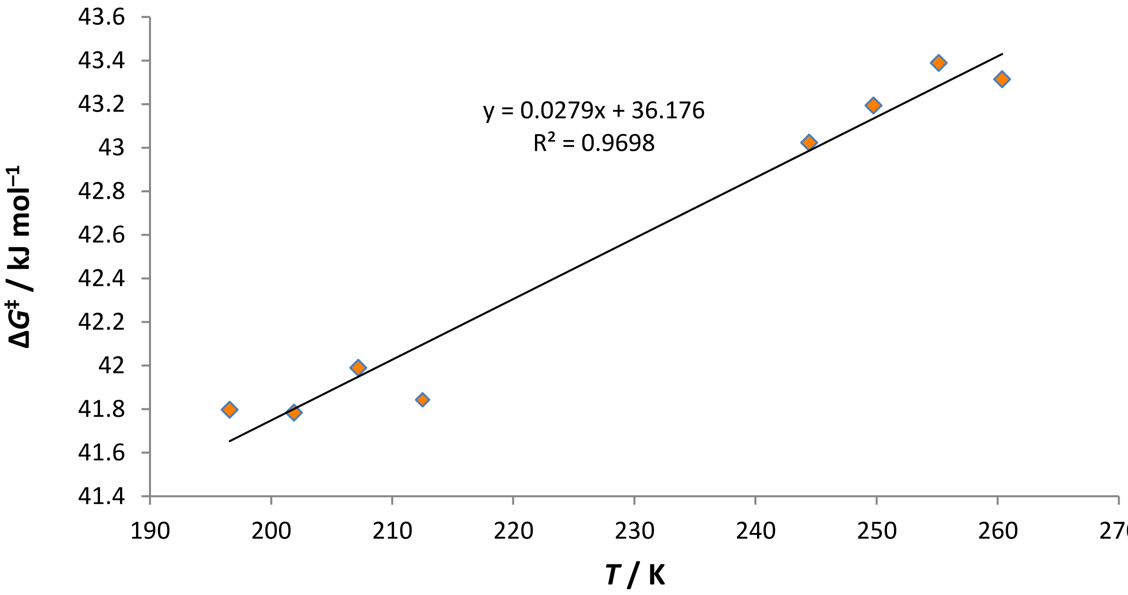

Figure S29. A plot of the experimentally measured free energy of activation $\Delta G^{\ddagger}$ for rotation of ring c of ***sym*-phenPh$_5$C$_7$H** as a function of temperature.

We also performed VT NMR measurements to determine the energy barrier to rotation of phenyl rings in **Ph$_7$C$_7$H-O**, using the same approach. Based on 2D NMR, we were able to assign ring d (Figure S30) as the phenyl group that experiences the highest energy barrier to rotation. A 20 mg

sample of **Ph$_7$C$_7$H-O** was dissolved in a mixture of CS_2 (0.7 mL) with CD_2Cl_2 (0.1 mL) as the NMR solvent and a series of ^{13}C NMR spectra ranging from 24–87 °C were recorded.

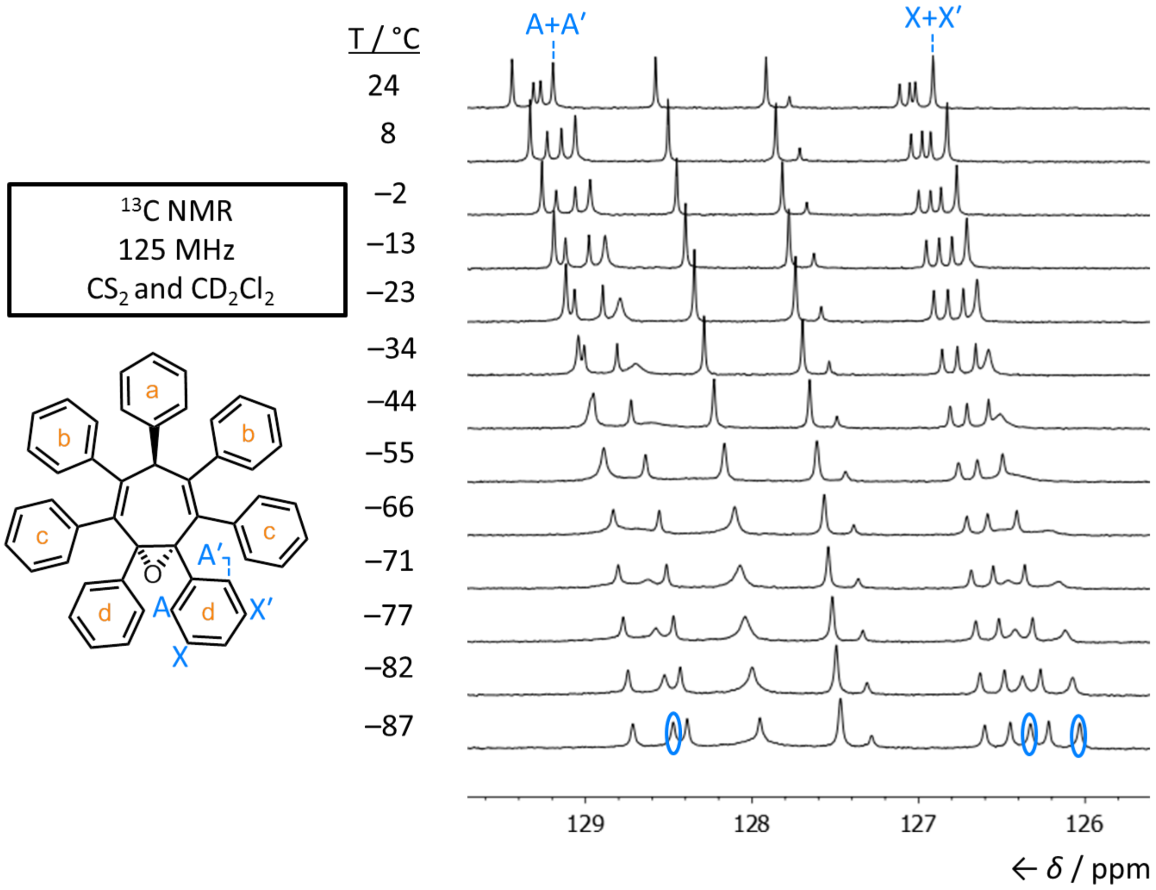

Figure S30. Partial ^{13}C VT NMR spectra of **Ph$_7$C$_7$H-O**. Peaks corresponding to the carbon pairs A/A′ and X/X′ are observed as individual, averaged signals in the fast exchange regime, but appear as distinct signals at low temperature in the slow exchange regime. Only three of the four signals can be distinguished at low temperature on account of overlapping signals; however, only one pair is needed to perform lineshape analysis.

Analysis of the ^{13}C spectra reveals that, within the temperature range studied, signals corresponding to ring d carbons broaden, merge into the baseline, and then re-emerge as four separate peaks as the temperature is decreased made up of two pairs of resonances in slow exchange. Only three resonances are observed as distinct peaks because the fourth overlaps with other signals. In order to determine which of the three re-emerged peaks are a pair a HSQC spectrum was acquired (Figure S30) at –87 °C. Both ^{1}H NMR and ^{13}C NMR spectra had been recorded from 24 °C to –87 °C.

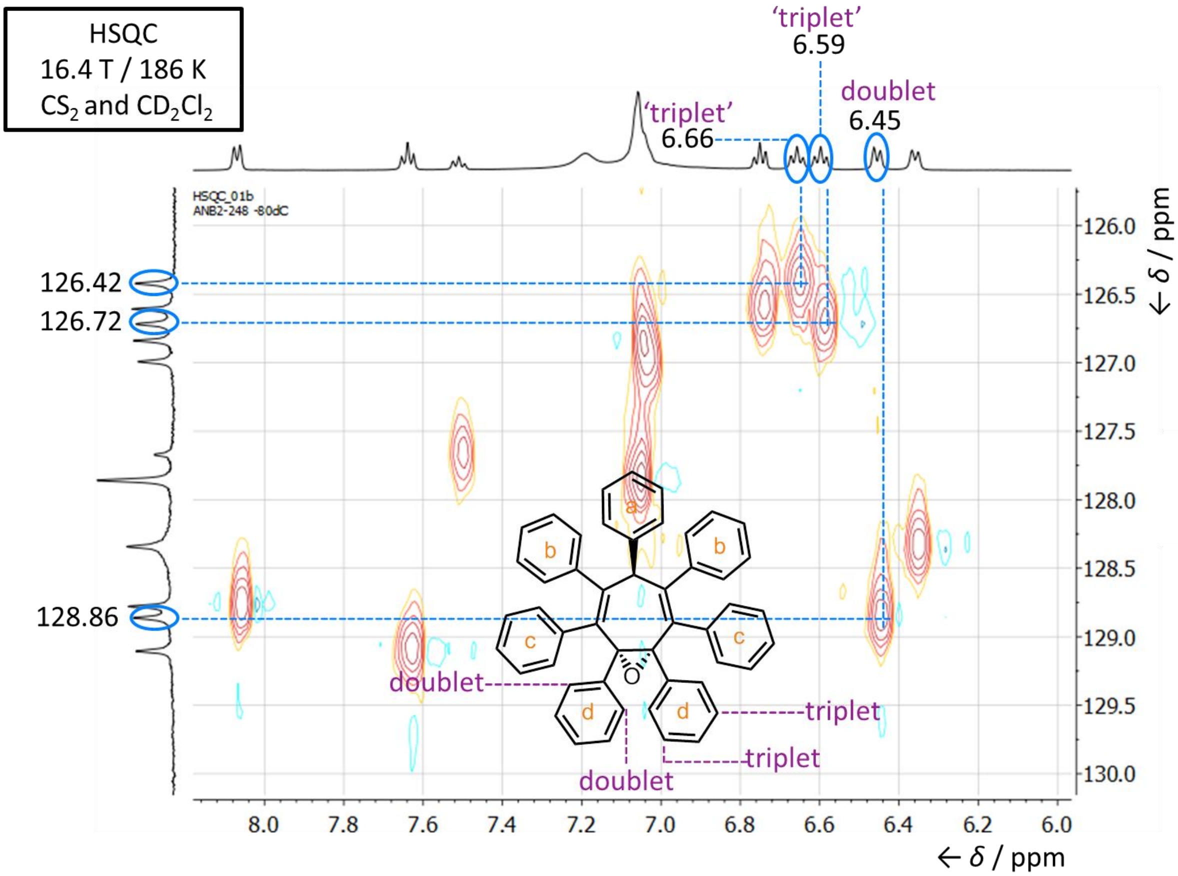

Figure S31. Partial HSQC NMR spectrum of **Ph_7C_7H-O** recorded at –87 °C. Correlations are highlighted between the ^{13}C signals observed to be in slow exchange by VT NMR (Figure S29) and the corresponding ^{1}H signals. Inset: based on the *J* coupling patterns, the different positions of ring d can be distinguished.

By matching the *J* coupling patterns of the ^{1}H signals following the approach described above for ***sym*-phenPh$_5$C$_7$H**, we can assign (Figure S32) the ^{13}C signals of **Ph_7C_7H-O** corresponding to X and X′. We selected five ^{13}C NMR spectra for further analysis, choosing temperatures close to the transition of ring d resonances from fast to the slow exchange regimes. As above, line shape analysis was performed to derive rate constants by comparison to model spectra produced using WinDNMR and activation energy barriers were calculated using equation S1.

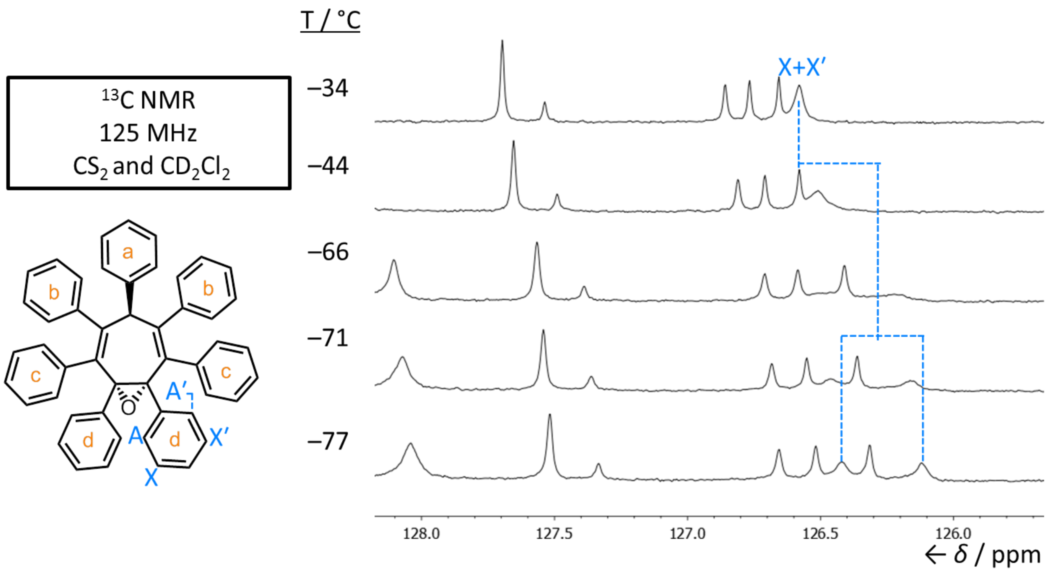

Figure S32. ^{13}C VT NMR spectra of **Ph_7C_7H-O** used for lineshape analysis. The dashed blue lines illustrate the resolution of a single peak into a pair of peaks as temperature decreases.

Table S2. Calculated activation energies ($\Delta G^{\ddagger}$) for the rotation of ring d of **Ph_7C_7H-O**.

Entry	Temperature / K (± 0.64)	$\Delta G^{\ddagger}$ / kJ mol^{-1}
1	239.2	42.2
2	228.5	42.3
3	207.2	42.7
4	201.9	42.9
5	196.2	43.6

A line was fitted (Figure S29) to a plot of $\Delta G^{\ddagger}$ vs T. The entropy of activation $\Delta S^{\ddagger}$ was measured to be +27.0 J mol^{-1} K^{-1} and enthalpy of activation $\Delta H^{\ddagger}$ was measured as 48.5 kJ mol^{-1}.

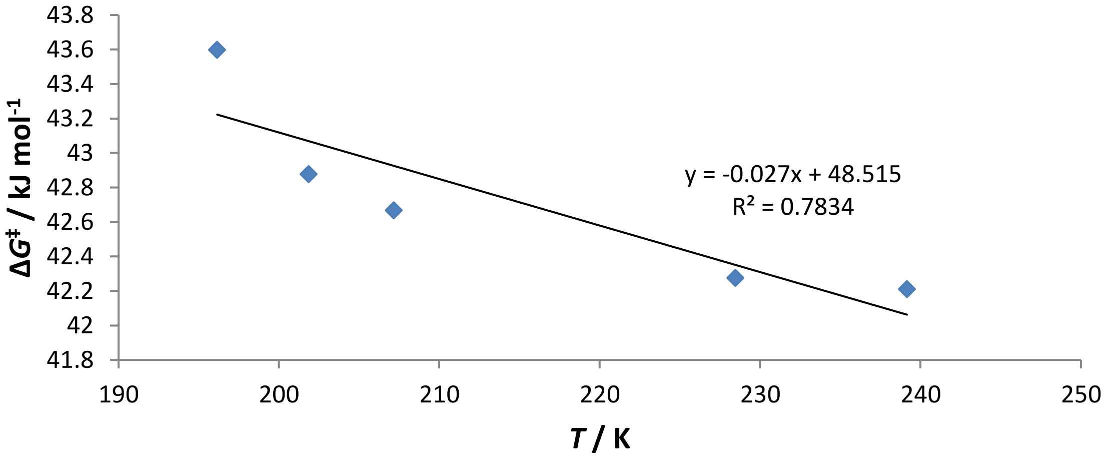

Figure S33. A plot of the experimentally measured free energy of activation $\Delta G^{\ddagger}$ for rotation of ring d of **Ph_7C_7H-O** as a function of temperature.

4. X-Ray Crystallographic Analysis

Analysis of all crystal structures and their packing are shown (Figure S34-47) with the crystal system, space group, unit cell parameters, bond lengths, bond angles and dihedral angles reported below. Direct comparisons of important bond lengths and centroid-centroid distance between the phenyl ring bound to the tertiary sp^3-centre and the opposite phenyl ring are outlined in Table S3 below. Notably, the C_5–C_6 distance is 1.47–1.49 Å for the triene compounds but 1.50–1.51 Å for the epoxides. This increase of 2–4 pm is characteristic of the increased single–bond character in the nonconjugated epoxides.

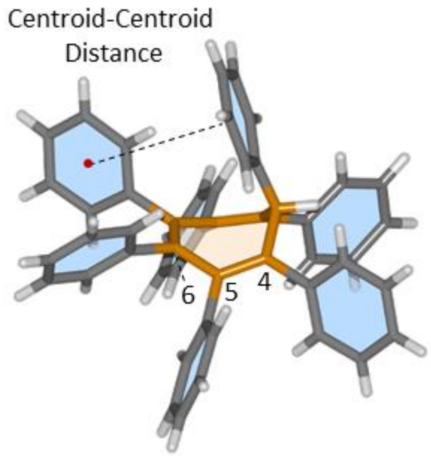

Figure S34. Solid-state structure of **Ph_7C_7H** viewed side-on to the seven-membered ring showing the centroid-centroid distance on the two rings associated with through-space dimers.

Table S3. Summarized bond lengths and centroid distances for the molecular rotors.

Rotor	Centroid-Centroid distance (Å)	C_4-C_5 distance (Å)	C_5-C_6 distance (Å)
C_7Ph_7H-O	5.21	1.34	1.50
sym-phenPh_5C_7H-O	4.99	1.36	1.51
asym-phenPh_5C_7H-O	5.07	1.34	1.50
C_7Ph_7H	5.15	1.35	1.47
sym-phenPh_5C_7H	4.57	1.35	1.49
asym-phenPh_5C_7H	5.02	1.35	1.49
sym-phen$_2Ph_3C_7H$-O	5.23	1.36	1.51

Analysis of the single-crystal X-ray structure of **Ph_7C_7H** has been reported previously.[3]

4.1 $Ph_3C_3 \cdot HCl_2$

Crystals of **$Ph_3C_3 \cdot HCl_2$** suitable for X-ray diffraction were grown by slow cooling of a saturated MeCN solution.

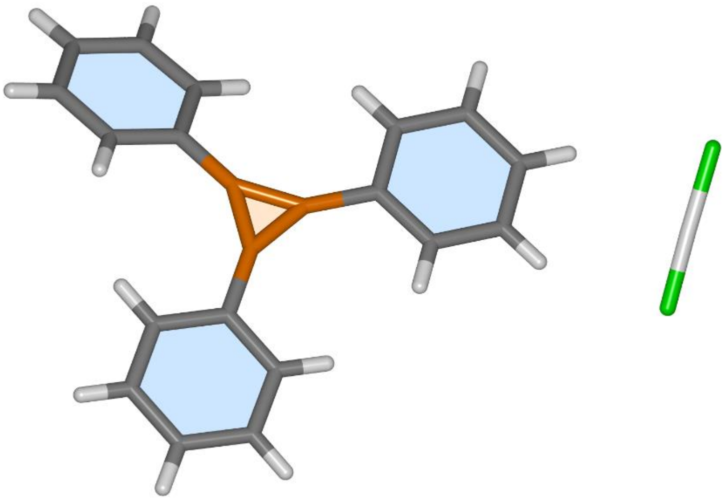

Figure S35. Solid-state structure of **$Ph_3C_3 \cdot HCl_2$**.

Crystal System: Triclinic
Space group: P-1
Unit Cell Parameters: a = 8.1608(7) Å, b = 9.0459(8) Å, c = 12.2953(10) Å, α = 95.447(3)°, β = 100.331(3)°, γ = 103.571(3)°, V = 859.06(13) $Å^3$, Z = 2

4.2 Ph_7C_7H-O

Crystals of **Ph_7C_7H-O** suitable for X-ray diffraction were grown by slow cooling of a saturated MeCN solution.

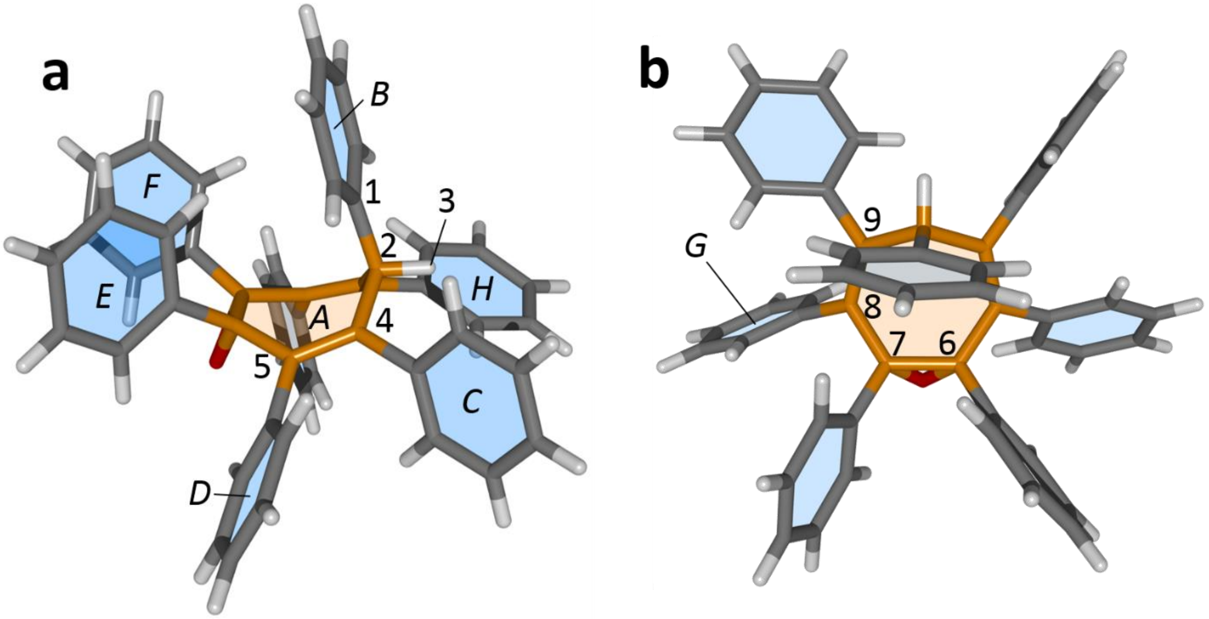

Figure S36. Solid-state structure of **Ph_7C_7H-O** viewed (a) side-on to the cycloheptatriene and (b) from above the cycloheptatriene. Selected atoms are labelled numerically, the planes of the carbocyclic rings are labelled by italicized uppercase letters. As central ring *A* is puckered, individual planes α^n are defined by a carbon vertex *n* and its two nearest neighbors within the ring, e.g., A^5 is the plane defined by atoms 4, 5, and 6.

Crystal System: Monoclinic
Space group: P21/c
Unit Cell Parameters: a = 15.9229(7) Å, b = 12.6701(5) Å, c = 17.9495(7) Å, β = 108.8982(16)°, V = 3426.02(24) Å3, Z = 4
Bond lengths (Å): C2–C4, 1.53; C4–C5, 1.34; C5–C6, 1.50; C6–C7, 1.49; C7–C8, 1.50; C8–C9, 1.34; C2–C9, 1.54.
Bond angles (°): C1–C2–H3, 104.4; C1–C2–C4, 117.6; C1–C2–C9, 113.9; C4–C2–C9, 110.6; C2–C4–C5, 123.5; C4–C5–C6, 123.7; C5–C6–C7, 123.4; C6–C7–C8, 122.6; C7–C8–C9, 124.5; C2–C9–C8, 123.4; C6–O–C7, 61.6.
Dihedral angles (°): A2–B, 36.1; A4–C, 60.2; A5–D, 55.9; A6–E, 76.6; A7–F, 73.0; A8–G, 78.6; A9–H, 52.5; B–C, 60.9; C–D, 53.9; D–E, 62.2; E–F, 58.9; F–G, 58.7; G–H, 64.8; B–H, 53.2.

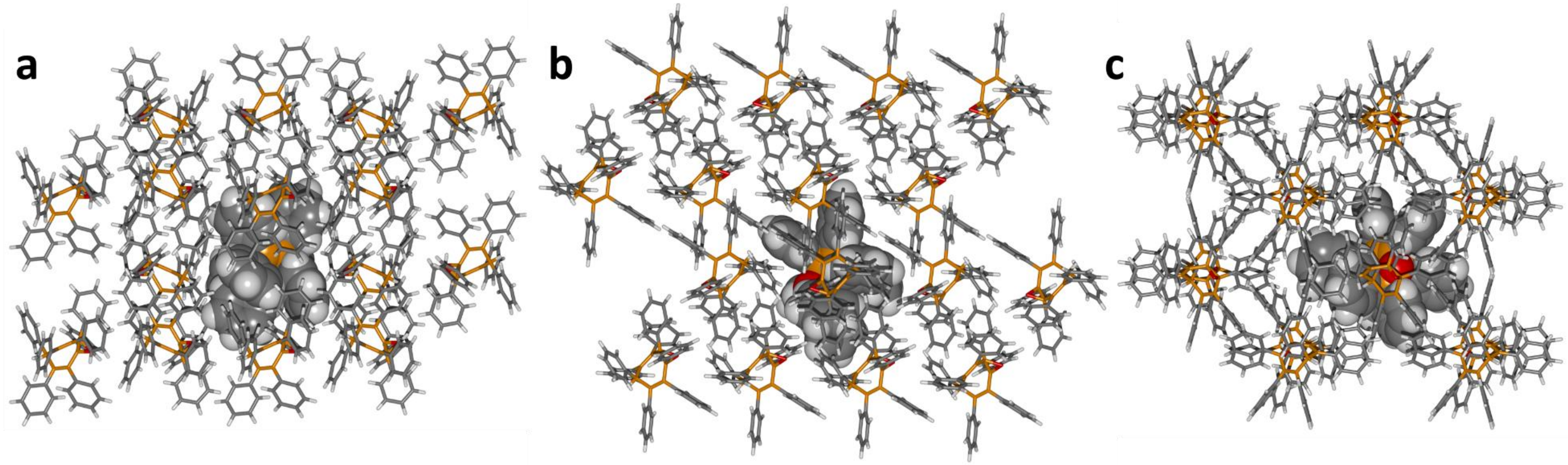

Figure S37. Solid-state superstructure of **Ph_7C_7H-O**. A central molecule (space filling representation) is shown embedded in a section of the lattice made up of 2×2×2 unit cells in order to illustrate the crystal packing. Projections are viewed along the crystallographic (a) *a*-, (b) *b*-, and (c) *c*-axes.

4.3 *sym*-phenPh_5C_7H

Crystals of ***sym*-phenPh$_5$C$_7$H** suitable for X-ray diffraction were grown by slow cooling of a saturated MeCN solution of pure ***sym*-phenPh$_5$C$_7$H**.

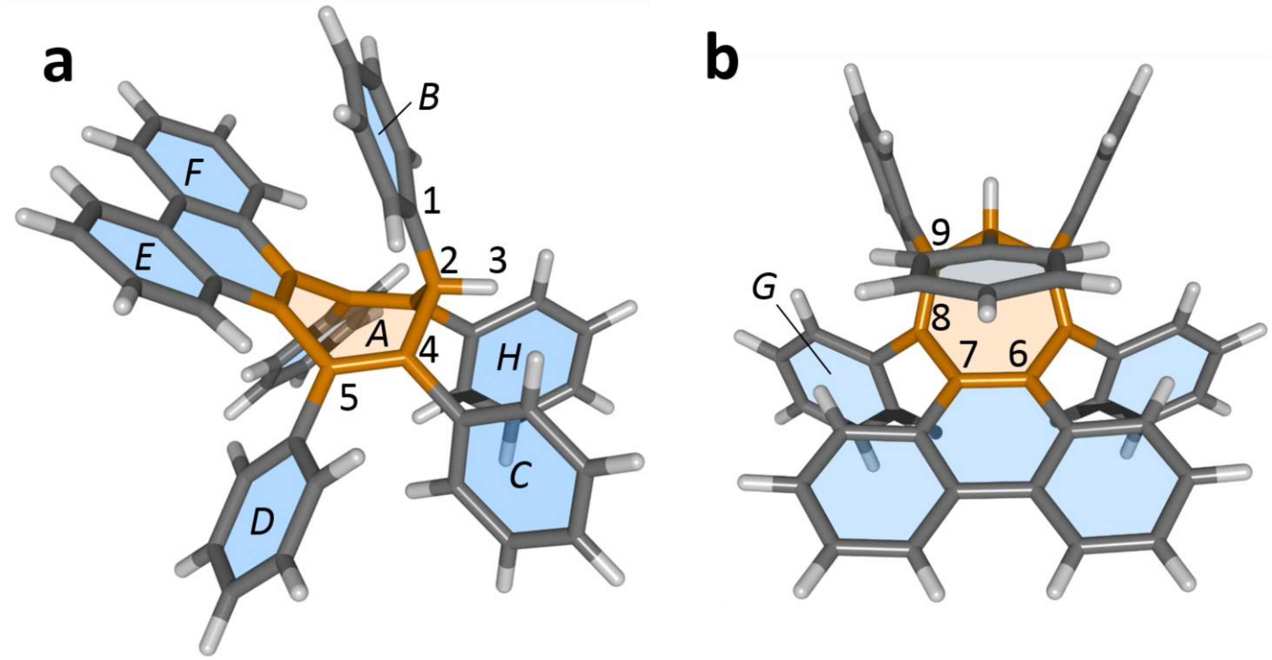

Figure S38. Solid-state structure of ***sym*-phenPh$_5$C$_7$H** viewed (a) side-on to the cycloheptatriene and (b) from above the cycloheptatriene. Selected atoms are labelled numerically, the planes of the carbocyclic rings are labelled by italicized uppercase letters. As central ring *A* is puckered, individual planes α^n are defined by a carbon vertex *n* and its two nearest neighbors within the ring, e.g., A^5 is the plane defined by atoms 4, 5, and 6.

Crystal System: Triclinic
Space group: P-1
Unit Cell Parameters: a = 9.4506(6) Å, b = 9.4870(6) Å, c = 19.2332(12) Å, α = 91.297(2)°, β = 103.684(2)°, γ = 91.779(2)°, V = 1673.85(18) Å3, Z = 2
Bond lengths (Å): C2–C4, 1.53; C4–C5, 1.35; C5–C6, 1.49; C6–C7, 1.38; C7–C8, 1.48; C8–C9, 1.36; C2–C9, 1.53.
Bond angles (°): C1–C2–H3, 107.3; C1–C2–C4, 116.6; C1–C2–C9, 116.5; C4–C2–C9, 99.9; C2–C4–C5, 119.8; C4–C5–C6, 120.9; C5–C6–C7, 121.5; C6–C7–C8, 121.3; C7–C8–C9, 120.9; C2–C9–C8, 119.6.
Dihedral angles (°): A2–B, 48.4; A4–C, 59.2; A5–D, 51.5; A6–E, 6.6; A7–F, 6.3; A8–G, 46.2; A9–H, 50.6; B–C, 65.0; C–D, 54.7; D–E, 83.2; E–F, 11.3; F–G, 82.6; G–H, 47.3; B–H, 73.6.

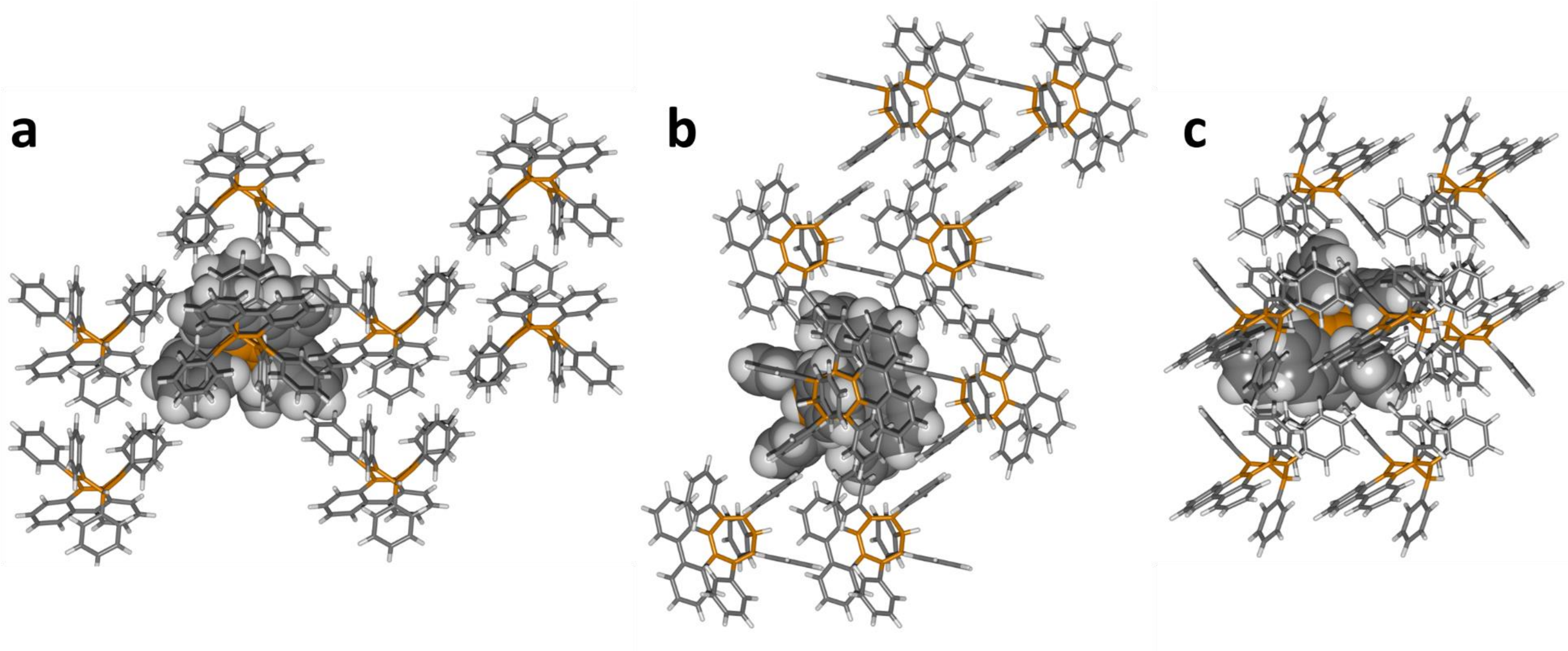

Figure S39. Solid-state superstructure of ***sym*-phenPh$_5$C$_7$H**. A central molecule (space filling representation) is shown embedded in a section of the lattice made up of 2×2×2 unit cells in order to illustrate the crystal packing. Projections are viewed along the crystallographic (a) *a*-, (b) *b*-, and (c) *c*-axes.

4.4 *asym*-phenPh$_5$C$_7$H

A single crystal of ***asym*-phenPh$_5$C$_7$H** suitable for X-ray diffraction were grown by slow cooling of a saturated MeCN solution of a 2:1 mixture of ***asym*-phenPh$_5$C$_7$H** and ***sym*-phenPh$_5$C$_7$H**.

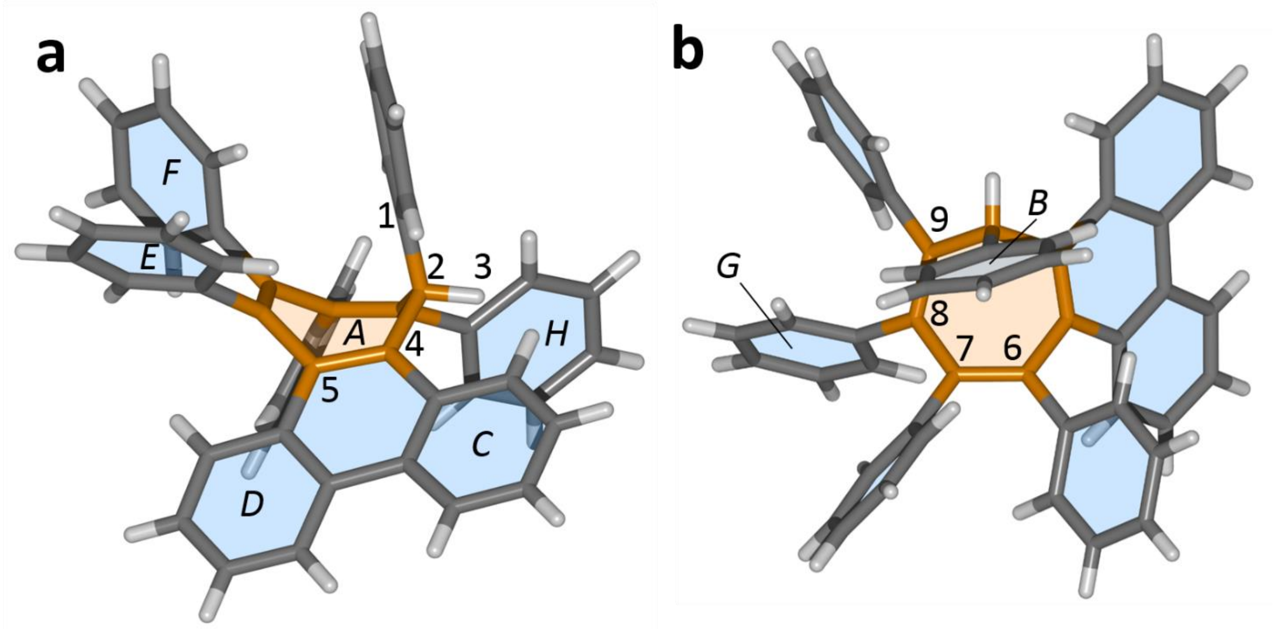

Figure S40. Solid-state structure of ***asym*-phenPh$_5$C$_7$H** viewed (a) side-on to the cycloheptatriene and (b) from above the cycloheptatriene. Selected atoms are labelled numerically, the planes of the carbocyclic rings are labelled by italicized uppercase letters. As central ring *A* is puckered, individual planes α^n are defined by a carbon vertex *n* and its two nearest neighbors within the ring, e.g., A^5 is the plane defined by atoms 4, 5, and 6.

Crystal System: Triclinic
Space group: P-1
Unit Cell Parameters: a = 9.6538(4) Å, b = 18.4913(7) Å, c = 20.4317(7) Å, α = 74.979(2)°, β = 77.015(2)°, γ = 74.871(2)°, V = 3351.98(22) Å^3, Z = 4
Bond lengths (Å): C2–C4, 1.53; C4–C5, 1.36; C5–C6, 1.49; C6–C7, 1.36; C7–C8, 1.49; C8–C9, 1.35; C2–C9, 1.53.
Bond angles (°): C1–C2–H3, 105.8; C1–C2–C4, 115.6; C1–C2–C9, 115.6; C4–C2–C9, 107.4; C2–C4–C5, 119.9; C4–C5–C6, 120.9; C5–C6–C7, 122.4; C6–C7–C8, 122.6; C7–C8–C9, 120.8; C2–C9–C8, 122.3.
Dihedral angles (°): A^2–*B*, 42.0; A^4–*C*, 11.4; A^5–*D*, 10.4; A^6–*E*, 47.5; A^7–*F*, 62.5; A^8–*G*, 69.5; A^9–*H*, 49.1; *B*–*C*, 76.6; *C*–*D*, 7.3; *D*–*E*, 87.2; *E*–*F*, 50.3; *F*–*G*, 63.4; *G*–*H*, 48.5; *B*–*H*, 74.6.

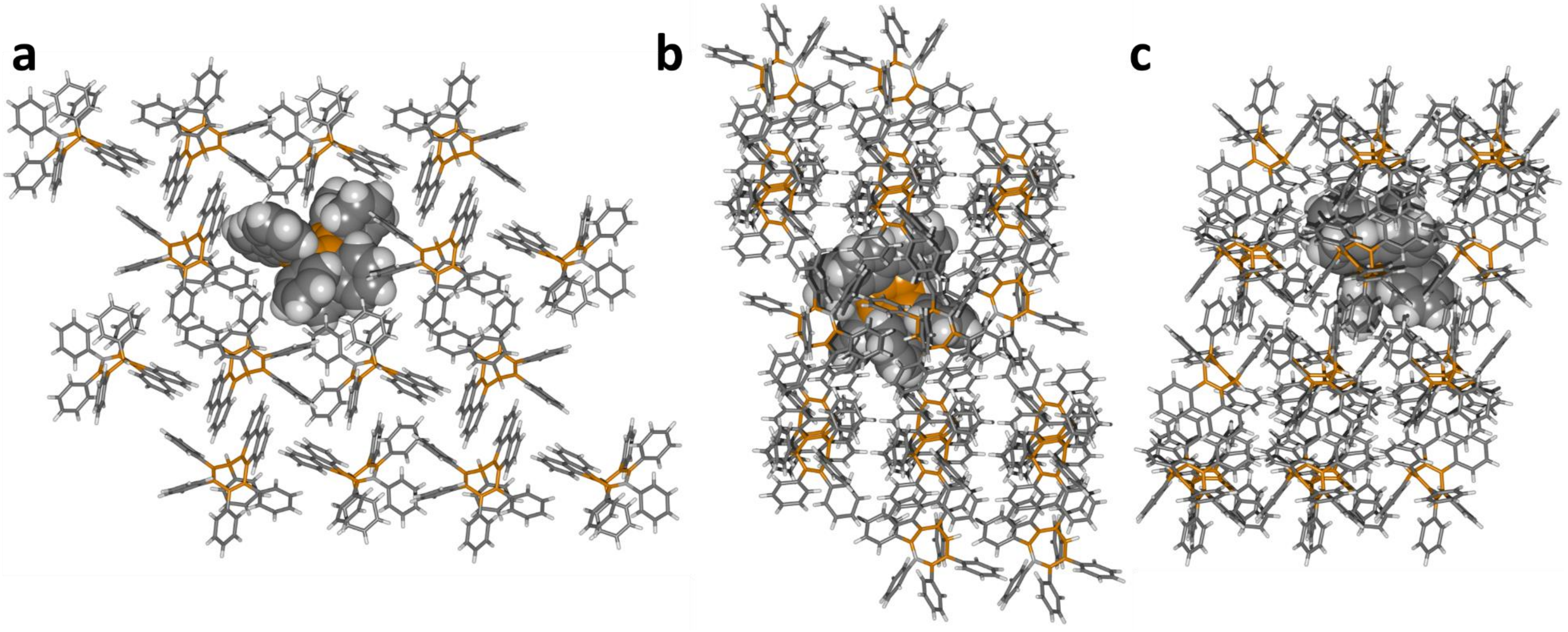

Figure S41. Solid-state superstructure of ***asym*-phenPh$_5$C$_7$H**. A central molecule (space filling representation) is shown embedded in a section of the lattice made up of 2×2×2 unit cells in order to illustrate the crystal packing. Projections are viewed along the crystallographic (a) *a*-, (b) *b*-, and (c) *c*-axes.

4.5 *sym*-phenPh_5C_7H-O

A single crystal of ***sym*-phenPh_5C_7H-O** suitable for X-ray diffraction were grown by slow cooling of a saturated MeCN solution.

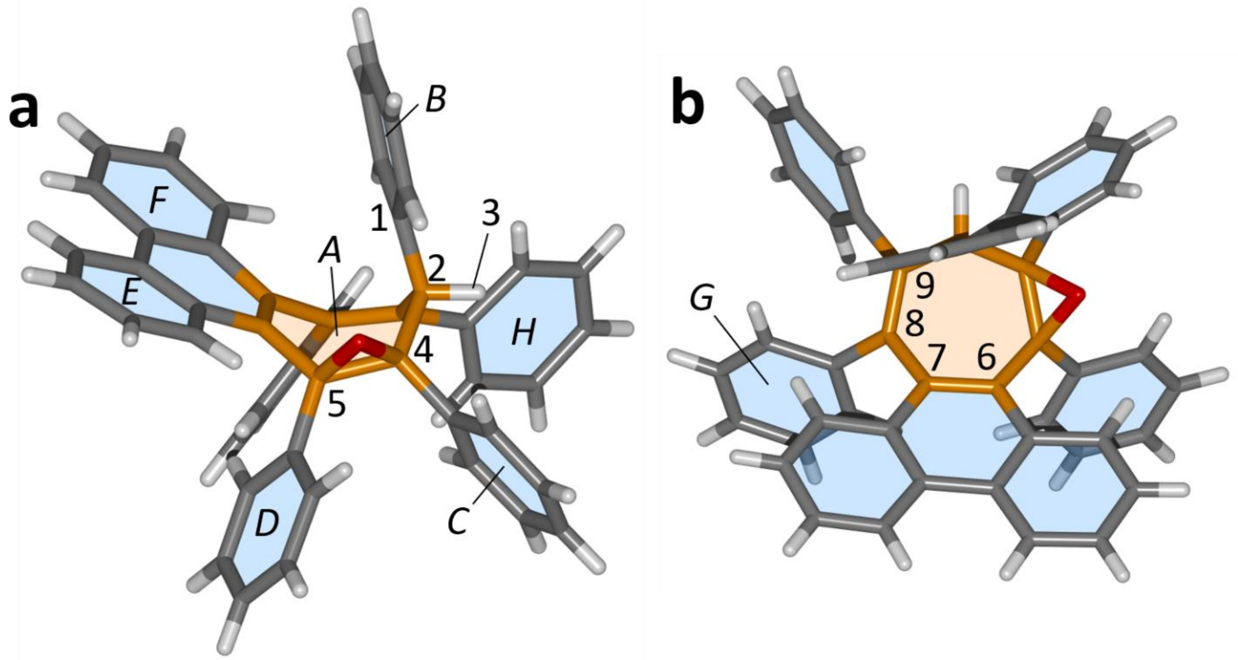

Figure S42. Solid-state structure of ***sym*-phenPh_5C_7H-O** viewed (a) side-on to the cycloheptatriene and (b) from above the cycloheptatriene. Selected atoms are labelled numerically, the planes of the carbocyclic rings are labelled by italicized uppercase letters. As central ring *A* is puckered, individual planes α^n are defined by a carbon vertex *n* and its two nearest neighbors within the ring, e.g., A^5 is the plane defined by atoms 4, 5, and 6.

Crystal System: Monoclinic
Space group: $P2_1/c$
Unit Cell Parameters: a = 9.6891(6) Å, b = 31.178(2) Å, c = 11.5786(8) Å, α = β = 109.132(3)° V = 3304.55(38) $Å^3$, Z = 4
Bond lengths (Å): C2–C4, 1.55; C4–C5, 1.48; C5–C6, 1.50; C6–C7, 1.37; C7–C8, 1.51; C8–C9, 1.35; C2–C9, 1.53.
Bond angles (°): C1–C2–H3, 105.3; C1–C2–C4, 118.8; C1–C2–C9, 114.5; C4–C2–C9, 106.5; C2–C4–C5, 120.1; C4–C5–C6, 119.3; C5–C6–C7, 122.6; C6–C7–C8, 123.5; C7–C8–C9, 121.7; C2–C9–C8, 120.3; C4–O–C5, 61.2.
Dihedral angles (°): A^2–*B*, 37.7; A^4–*C*, 81.6; A^5–*D*, 51.5; A^6–*E*, 5.9; A^7–*F*, 10.4; A^8–*G*, 55.2; A^9–*H*, 47.1; *B*–*C*, 30.4; *C*–*D*, 63.3; *D*–*E*, 82.4; *E*–*F*, 10.1; *F*–*G*, 83.4; *G*–*H*, 41.8; *B*–*H*, 72.0.

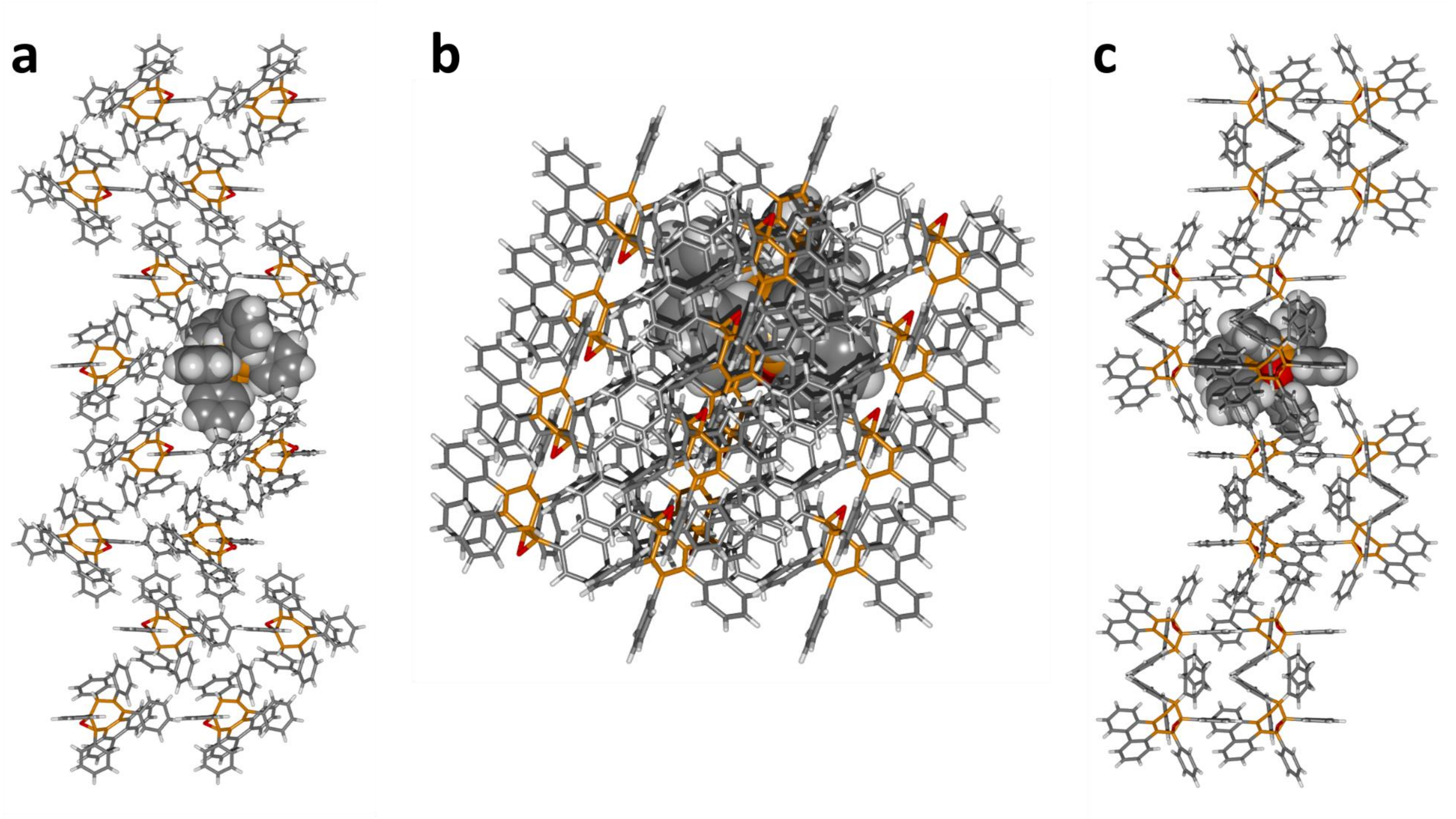

Figure S43. Solid-state superstructure of ***sym*-phenPh$_5$C$_7$H-O**. A central molecule (space filling representation) is shown embedded in a section of the lattice made up of 2×2×2 unit cells in order to illustrate the crystal packing. Projections are viewed along the crystallographic (a) *a*-, (b) *b*-, and (c) *c*-axes.

4.6 *asym*-phenPh_5C_7H-O

A single crystal of ***asym*-phenPh_5C_7H-O** suitable for X-ray diffraction were grown by slow cooling of a saturated MeCN solution.

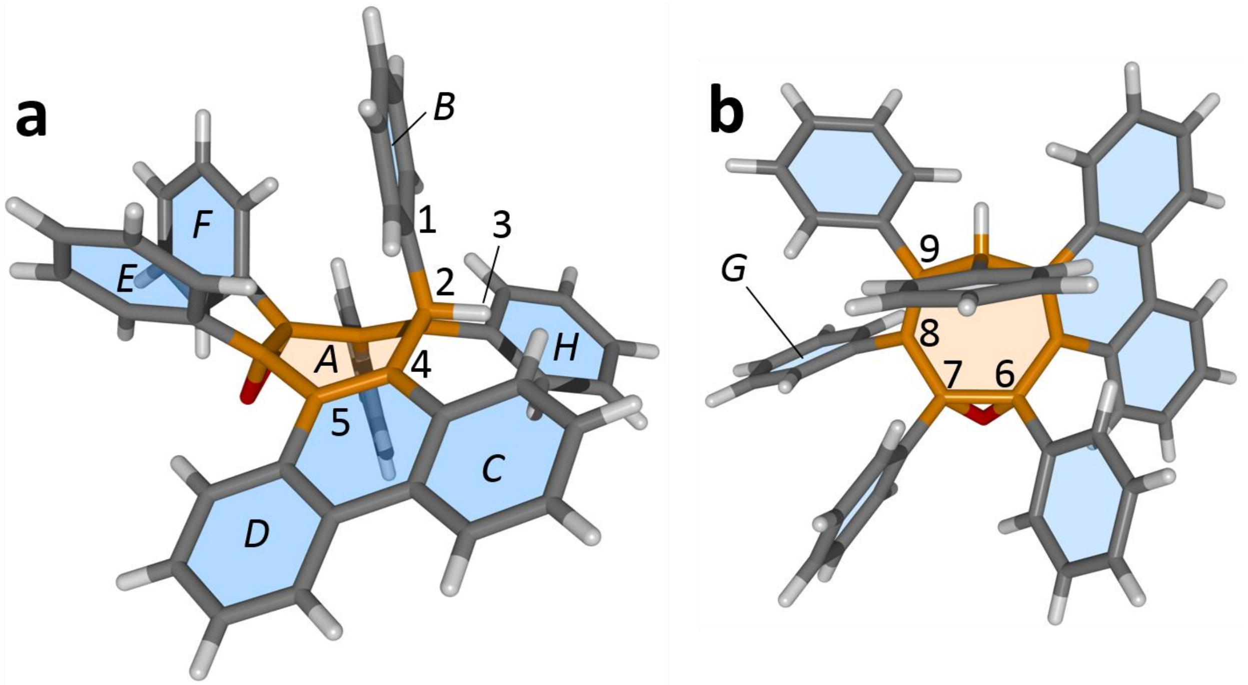

Figure S44. Solid-state structure of ***asym*-phenPh_5C_7H-O** viewed (a) side-on to the cycloheptatriene and (b) from above the cycloheptatriene. Selected atoms are labelled numerically, the planes of the carbocyclic rings are labelled by italicized uppercase letters. As central ring *A* is puckered, individual planes α^n are defined by a carbon vertex *n* and its two nearest neighbors within the ring, e.g., A^5 is the plane defined by atoms 4, 5, and 6.

Crystal System: Orthorhombic
Space group: $P2_12_12_1$
Unit Cell Parameters: a = 10.1203(6) Å, b = 16.5120(9) Å, c = 20.0664(11) Å, V = 3353.22(33) $Å^3$, Z = 4
Bond lengths (Å): C2–C4, 1.53; C4–C5, 1.36; C5–C6, 1.51; C6–C7, 1.49; C7–C8, 1.50; C8–C9, 1.34; C2–C9, 1.53.
Bond angles (°): C1–C2–H3, 103.8; C1–C2–C4, 114.8; C1–C2–C9, 115.8; C4–C2–C9, 113.0; C2–C4–C5, 121.8; C4–C5–C6, 123.4; C5–C6–C7, 123.4; C6–C7–C8, 122.7; C7–C8–C9, 123.2; C2–C9–C8, 125.5; C6–O–C7, 61.6.
Dihedral angles (°): A^2–*B*, 34.4; A^4–*C*, 9.4; A^5–*D*, 2.9; A^6–*E*, 47.7; A^7–*F*, 88.4; A^8–*G*, 76.3; A^9–*H*, 56.9; *B*–*C*, 76.0; *C*–*D*, 6.3; *D*–*E*, 88.3; *E*–*F*, 53.1; *F*–*G*, 40.5; *G*–*H*, 59.3; *B*–*H*, 56.2.

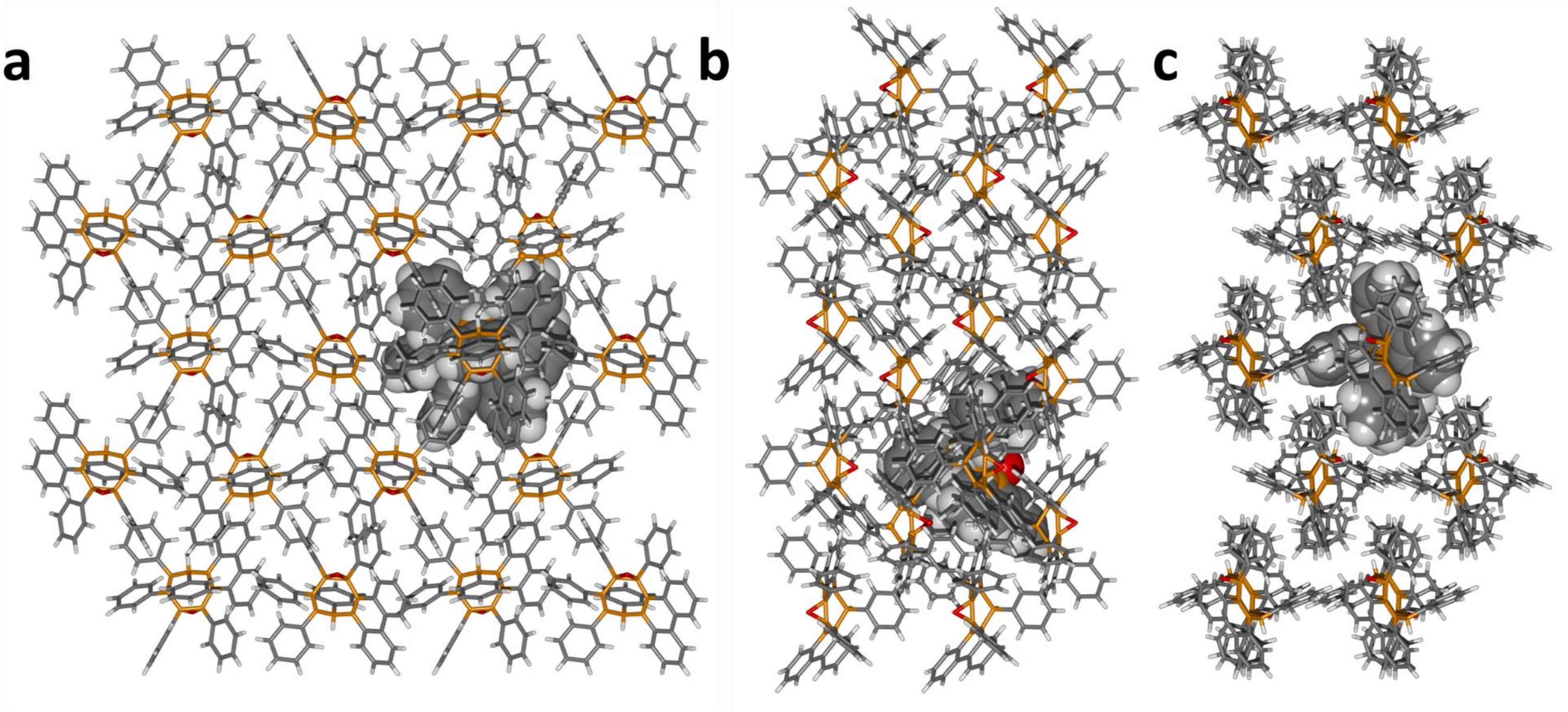

Figure S45. Solid-state superstructure of ***asym*-phenPh$_5$C$_7$H-O**. A central molecule (space filling representation) is shown embedded in a section of the lattice made up of 2×2×2 unit cells in order to illustrate the crystal packing. Projections are viewed along the crystallographic (a) *a*-, (b) *b*-, and (c) *c*-axes.

4.7 sym-phen$_2$Ph$_3$C$_7$H-O

A single crystal of ***sym*-phenPh$_5$C$_7$H-O** suitable for X-ray diffraction were grown by slow cooling of a saturated MeCN solution.

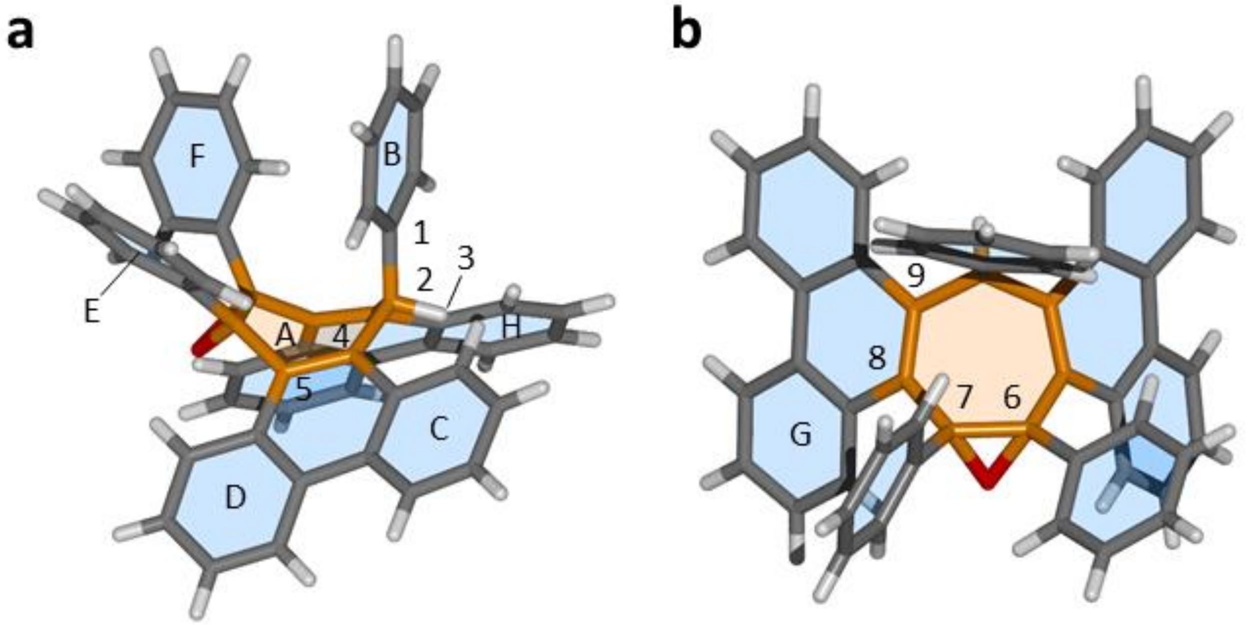

Figure S46. Solid-state structure of ***sym*-phen$_2$Ph$_3$C$_7$H-O** viewed (a) side-on to the seven-membered ring and (b) from above the seven-membered ring. Selected atoms are labelled numerically, the planes of the carbocyclic rings are labelled by italicized uppercase letters. As central ring *A* is puckered, individual planes α^n are defined by a carbon vertex *n* and its two nearest neighbours within the ring, e.g., A^5 is the plane defined by atoms 4, 5, and 6.

Crystal System: Monoclinic
Space group: $P2_1/n$
Unit Cell Parameters: a = 10.4829(6) Å, b = 13.2982(7) Å, c = 22.9502(13) Å, V = 3197.6(3) Å^3, Z = 4
Bond lengths (Å): C2–C4, 1.54; C4–C5, 1.36; C5–C6, 1.51; C6–C7, 1.50; C7–C8, 1.50; C8–C9, 1.37; C2–C9, 1.54.
Bond angles (°): C1–C2–H3, 102.9; C1–C2–C4, 115.4; C1–C2–C9, 117.6; C4–C2–C9, 112.4; C2–C4–C5, 122.9; C4–C5–C6, 121.4; C5–C6–C7, 120.7; C6–C7–C8, 119.41; C7–C8–C9, 121.3; C2–C9–C8, 124.0; C6–O–C7, 59.21.
Dihedral angles (°): A^2–*B*, 34.1; A^4–*C*, 12.9; A^5–*D*, 4.4; A^6–*E*, 67.6; A^7–*F*, 104.2; A^8–*G*, 10.9; A^9–*H*, 44.8; *B*–*C*, 97.4; *C*–*D*, 11.4; *D*–*E*, 76.7; *E*–*F*, 4.3; *F*–*G*, 73.9; *G*–*H*, 14.4; *B*–*H*, 77.2.

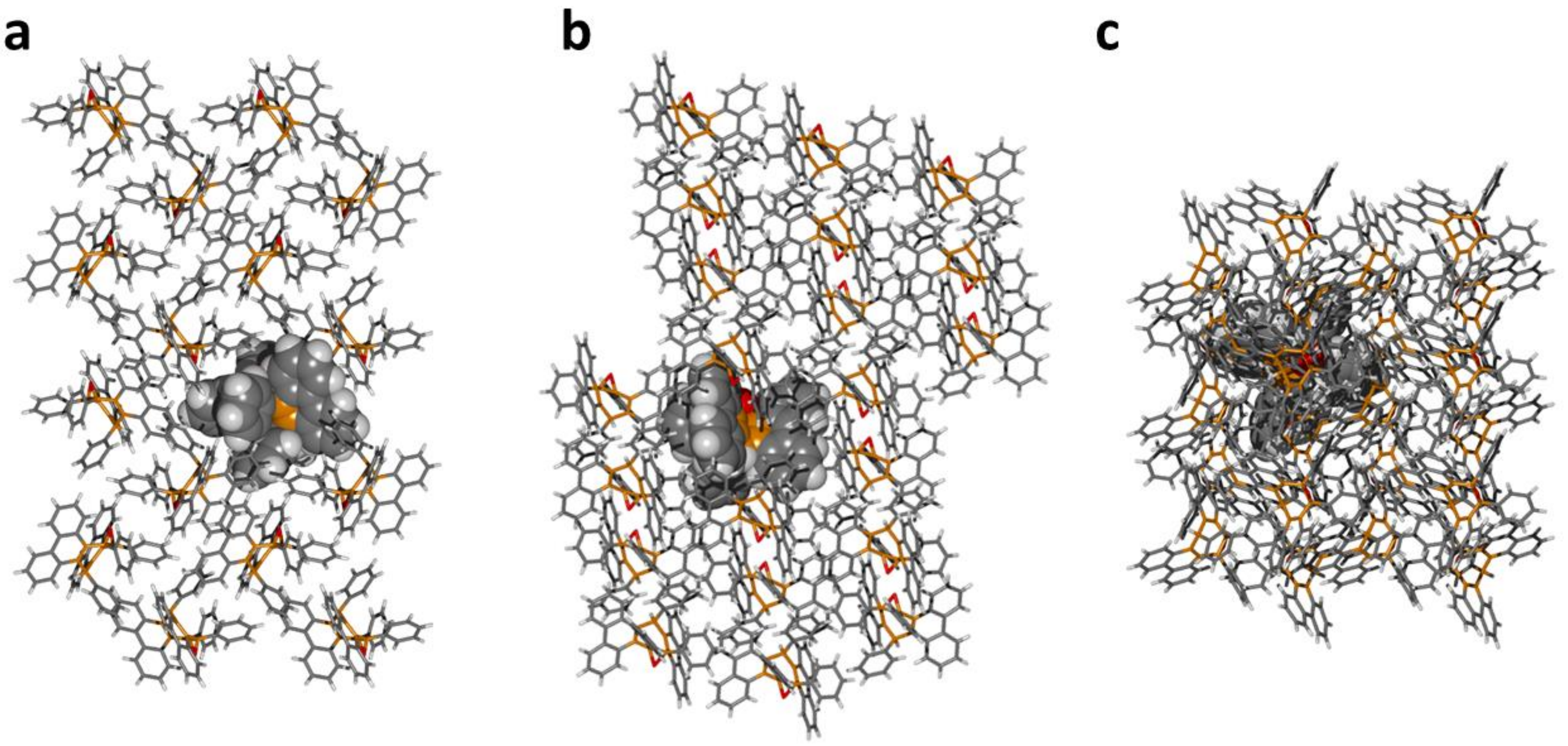

Figure S47. Solid-state superstructure of ***sym*-phen$_2$Ph$_3$C$_7$H-O**. A central molecule (space filling representation) is shown embedded in a section of the lattice made up of 2×2×2 unit cells in order to illustrate the crystal packing. Projections are viewed along the crystallographic (a) *a*-, (b) *b*-, and (c) *c*-axes.

5. UV-Vis Absorption Spectra

All spectra show (Figure S48) that the UV-Vis absorption characteristics of the carbocycles are independent of the choice of solvent. UV-Vis measurements were performed using anhydrous solvents – CH_2Cl_2, methylcyclohexane (MCH), MeCN, MeOH, tetrahydrofuran (THF) or 2-methyltetrahydrofuran (2-MeTHF). Spectra were measured in 10 mm path-length cuvette at room temperature. 10 μM Sample concentrations of carbocycles **Ph_7C_7H** and 20 μM sample concentrations of carbocycles (**Ph_7C_7H-O**, ***sym*-phenPh_5C_7H**, ***asym*-phenPh_5C_7H**, ***sym*-phenPh_5C_7H-O** and ***asym*-phenPh_5C_7H-O**) were used for all measurements in Figure S42. The intensities were plotted as molar absorptivity ε defined by the formula:

$$\varepsilon = \frac{I}{c \cdot l}$$

where *I* is the measured intensity, *c* is the concentration of sample, and *l* is path length of the cuvette.

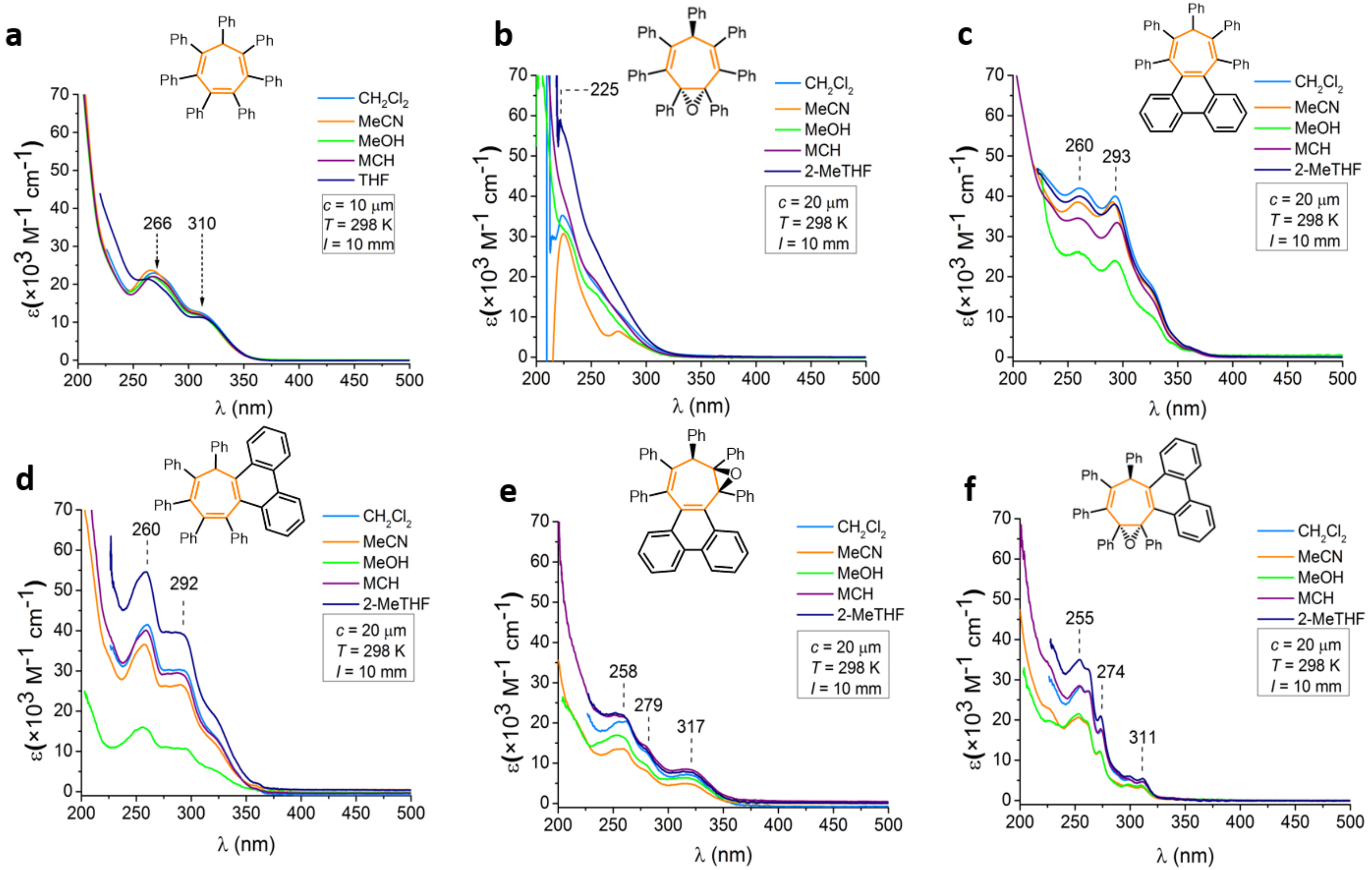

Figure S48. UV-Vis absorption spectra of solutions of (a) **Ph_7C_7H,** (b) **Ph_7C_7H-O**, (c) ***sym*-phenPh_5C_7H**, (d) ***asym*-phenPh_5C_7H**, (e) ***sym*-phenPh_5C_7H-O**, (f) ***asym*-phenPh_5C_7H-O** in a series of non-polar and polar media.

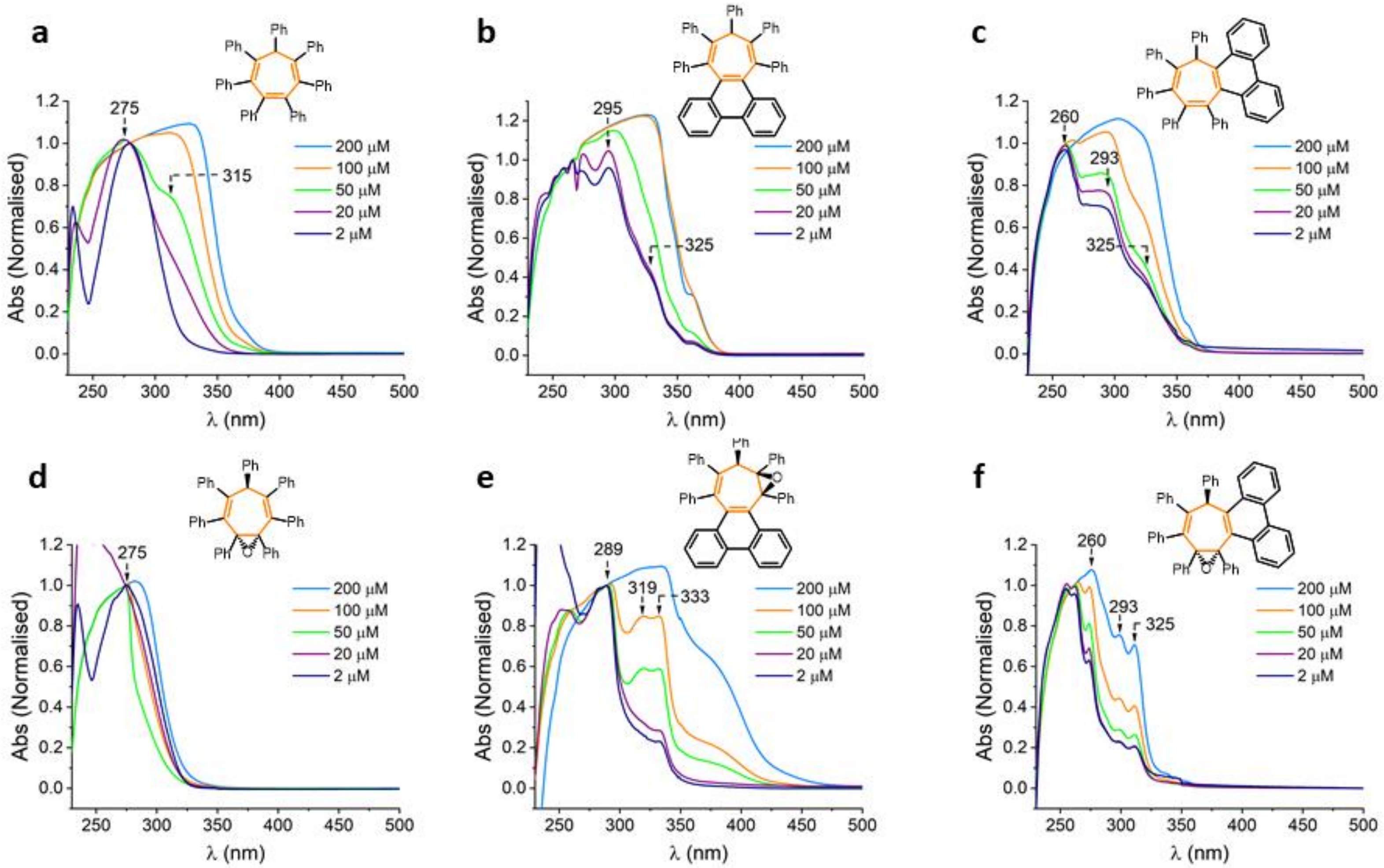

Figure S49. UV-Vis absorption spectra of solutions of (a) **Ph_7C_7H** (b) **Ph_7C_7H-O** (c) ***sym*-phenPh$_5$C$_7$H** (d) ***asym*-phenPh$_5$C$_7$H**, (e) ***sym*-phenPh$_5$C$_7$H-O**, (f) ***asym*-phenPh$_5$C$_7$H-O** at a series of concentrations in 2-MeTHF.

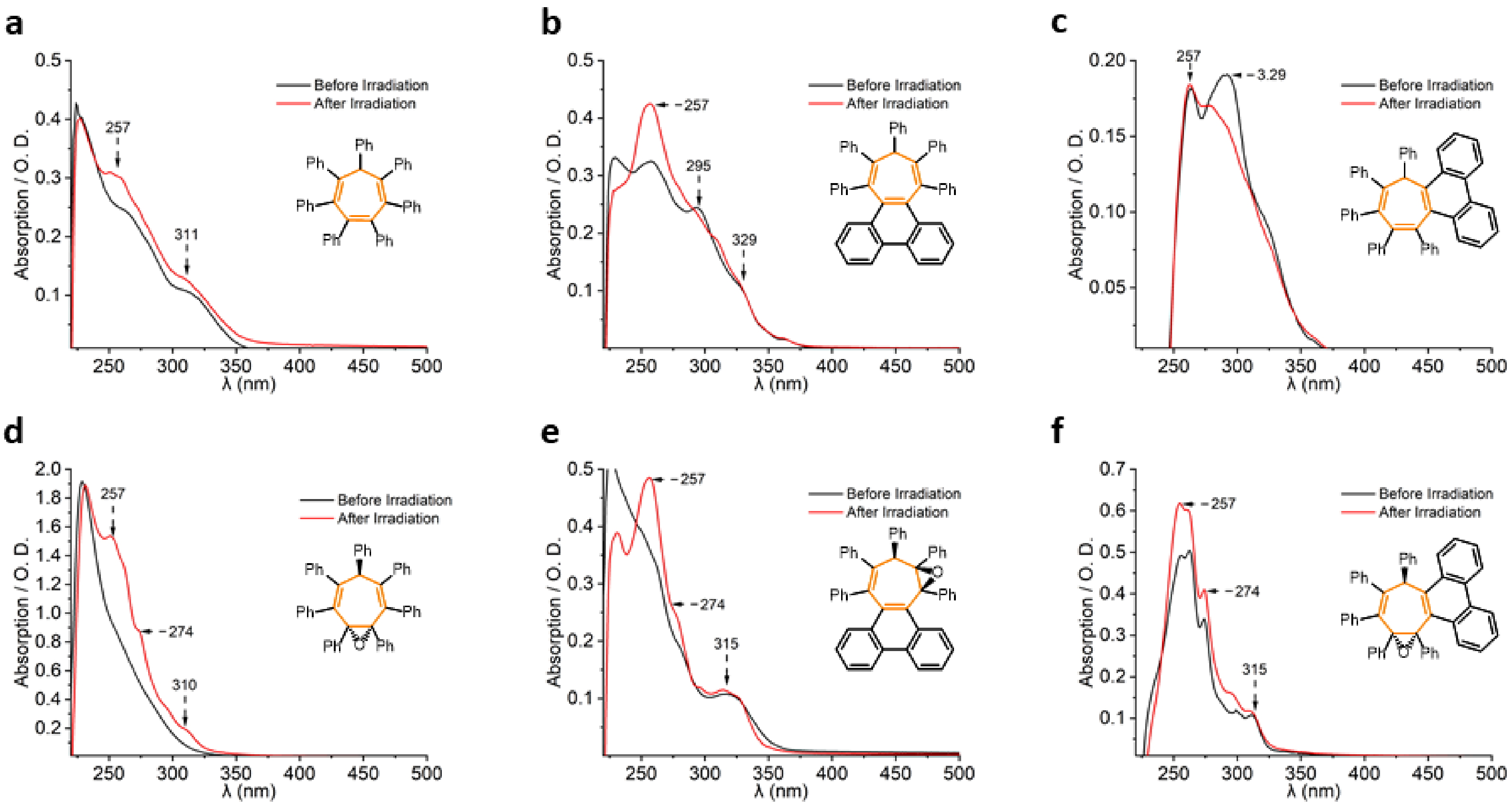

Figure S50. UV-Vis absorption spectra of solutions of a) **Ph_7C_7H** b) ***sym*-phenPh$_5$C$_7$H** c) ***asym*-phenPh$_5$C$_7$H**, d) **Ph_7C_7H-O** e) ***sym*-phenPh$_5$C$_7$H-O**, f) ***asym*-phenPh$_5$C$_7$H-O** in MeCN before and after irradiation under 4.0 eV light for 10 min for d-f and 1h for a-c.

6. Variable-Temperature Fluorescence

Samples for VT fluorescence were prepared using 200 μM THF stock solutions. The desired quantity was measured into a vial, evaporated to dryness, then the solid residue diluted to 20 μM and 2 μM concentration in the solvent used for the measurements. Anhydrous 2-MeTHF were used as the solvent system because it forms a stable organic glass which is UV-vis transparent at low temperatures. The samples were always kept at each temperature for at least 10 min to equilibrate prior to recording the spectra. The results of VT fluorescence are summarized in Figures S51-54.

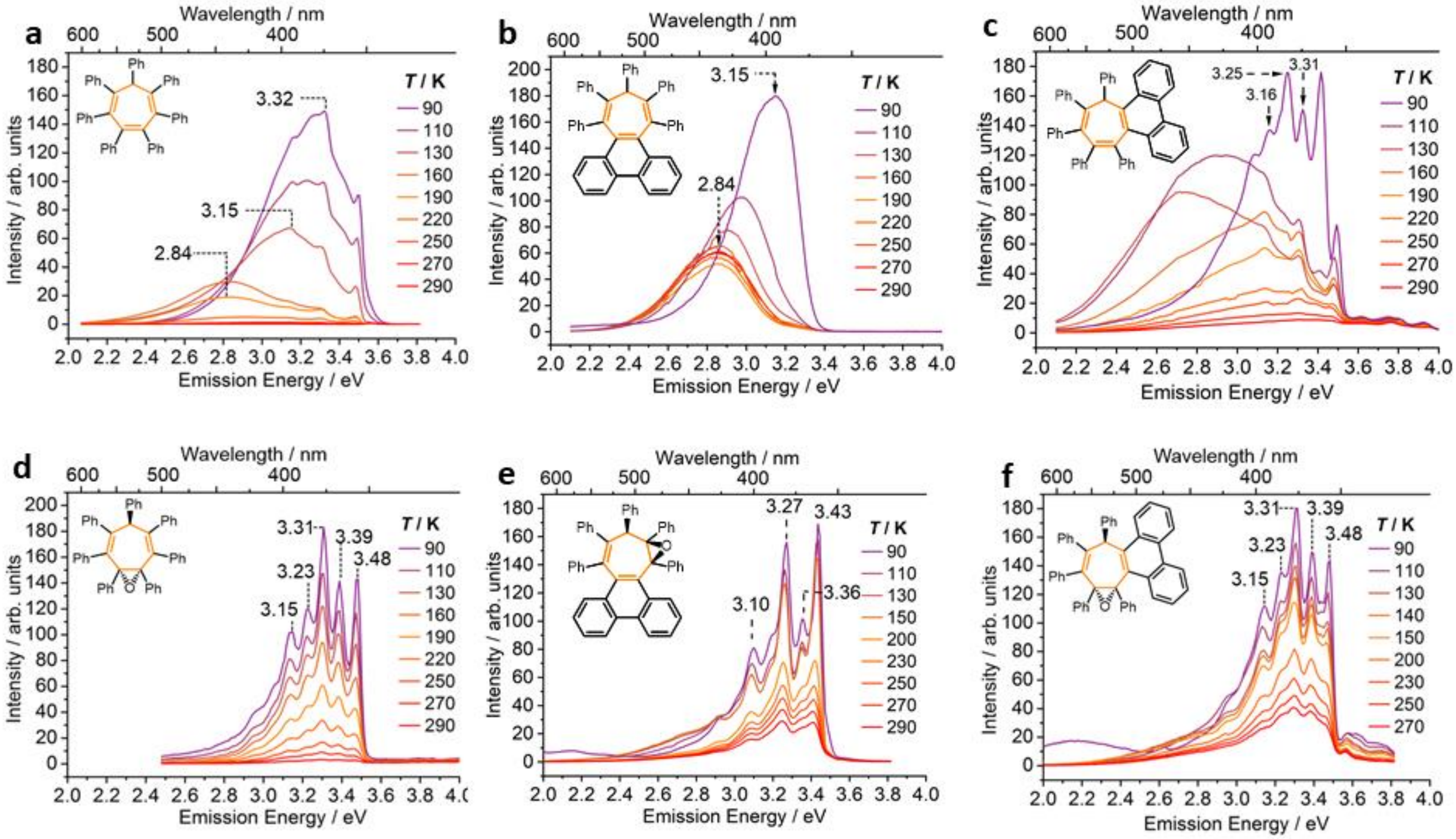

Figure S51. VT fluorescence spectra of 2-MeTHF solutions (*l* = 10 mm, *T* = 90–290 K) of (a) **Ph_7C_7H** λ_{ex} = 315 nm, *c* = 10 μM; (b) **Ph_7C_7H-O** λ_{ex} = 280 nm, *c* = 20 μM; (c) ***sym*-phenPh_5C_7H** λ_{ex} = 300 nm, *c* = 20 μM; (d) ***asym*-phenPh_5C_7H** λ_{ex} = 315 nm, *c* = 20 μM; (e) ***sym*-phenPh_5C_7H-O** λ_{ex} = 315 nm, *c* = 20 μM; (f) ***asym*-phenPh_5C_7H-O** λ_{ex} = 315 nm, *c* = 20 μM. Inset in panel (a): legend for temperatures in all panels.

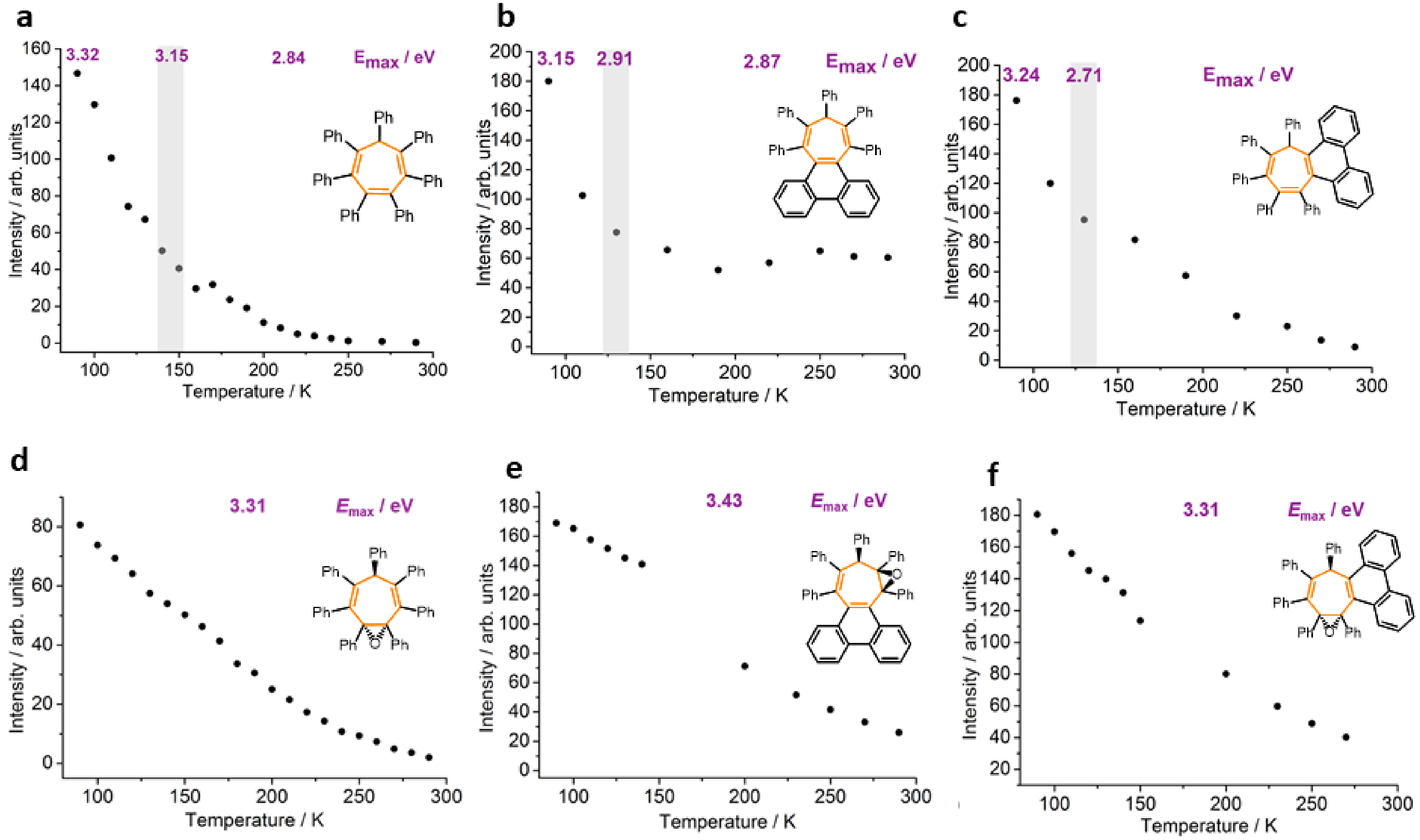

Figure S52. Peak emission intensities from VT fluorescence spectra of 2-MeTHF solutions (l = 10 mm, T = 90–290 K) of (a) **Ph_7C_7H** λ_{ex} = 315 nm, c = 10 μM; (b) **Ph_7C_7H-O** λ_{ex} = 280 nm, c = 20 μM; (c) ***sym*-phenPh_5C_7H** λ_{ex} = 300 nm, c = 20 μM; (d) ***asym*-phenPh_5C_7H** λ_{ex} = 315 nm, c = 20 μM; (e) ***sym*-phenPh_5C_7H-O** λ_{ex} = 315 nm, c = 20 μM; (e) ***asym*-phenPh_5C_7H-O** λ_{ex} = 315 nm, c = 20 μM. The emission energy of the most intense peak at each temperature was chosen. These energies are given on each panel. The most intense emission peaks of **Ph_7C_7H** and ***sym*-phenPh_5C_7H** vary with temperature, so the plots are divided into sections corresponding to different peak energies.

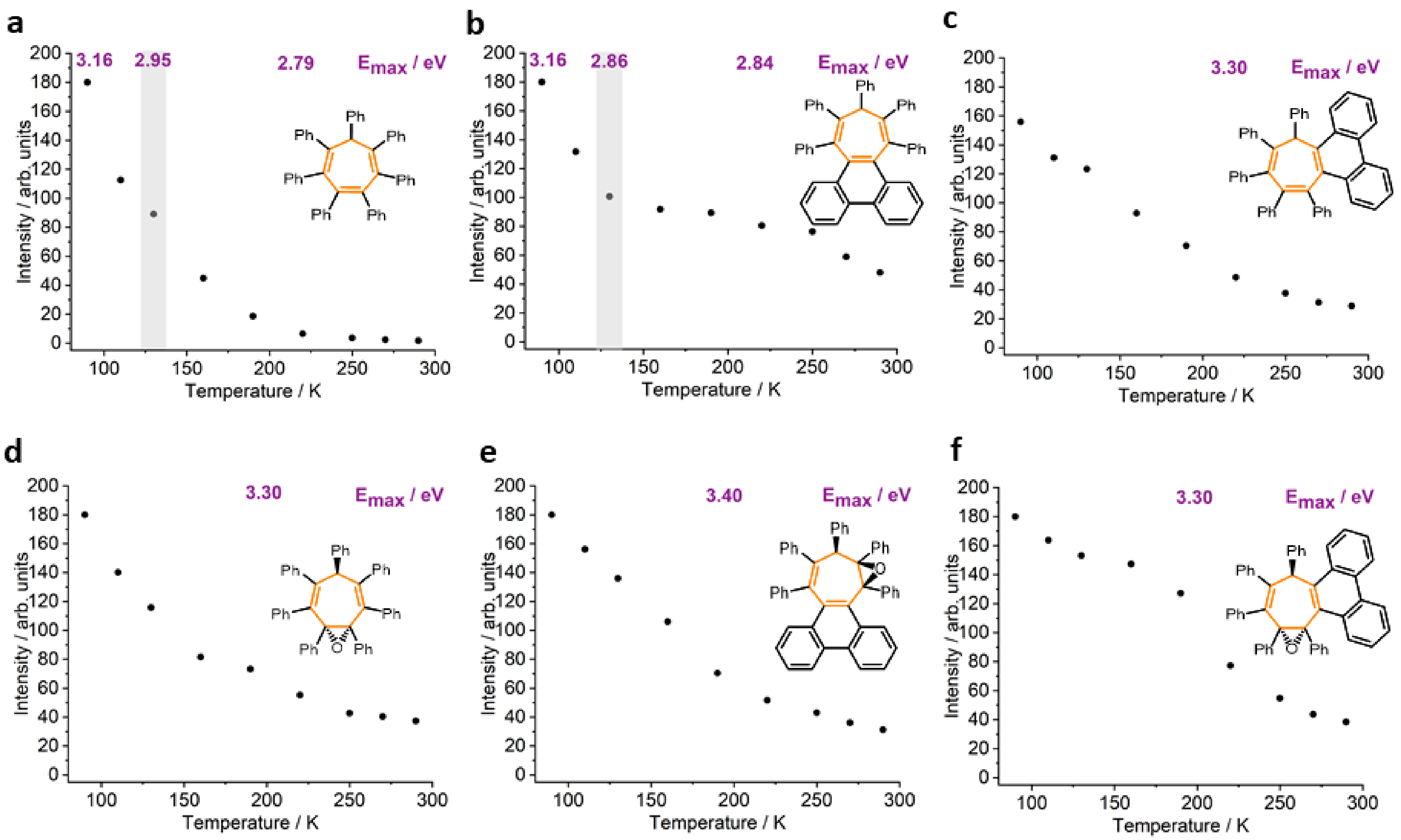

Figure S53. Peak emission intensities from VT fluorescence spectra of 2-MeTHF solutions (*l* = 10 mm, *T* = 90–290 K) of (a) $\mathbf{Ph_7C_7H}$ λ_{ex} = 315 nm, *c* = 2 µM; (b) $\mathbf{Ph_7C_7H\text{-}O}$ λ_{ex} = 280 nm, *c* = 2 µM; (c) ***sym*-phenPh$_5$C$_7$H** λ_{ex} = 300 nm, *c* = 2 µM; (d) ***asym*-phenPh$_5$C$_7$H** λ_{ex} = 315 nm, *c* = 2 µM; (e) ***sym*-phenPh$_5$C$_7$H-O** λ_{ex} = 315 nm, *c* = 2 µM; (e) ***asym*-phenPh$_5$C$_7$H-O** λ_{ex} = 315 nm, *c* = 2 µM. The emission energy of the most intense peak at each temperature was chosen. These energies are given on each panel. The most intense emission peaks of $\mathbf{Ph_7C_7H}$ and ***sym*-phenPh$_5$C$_7$H** vary with temperature, so the plots are divided into sections corresponding to different peak energies.

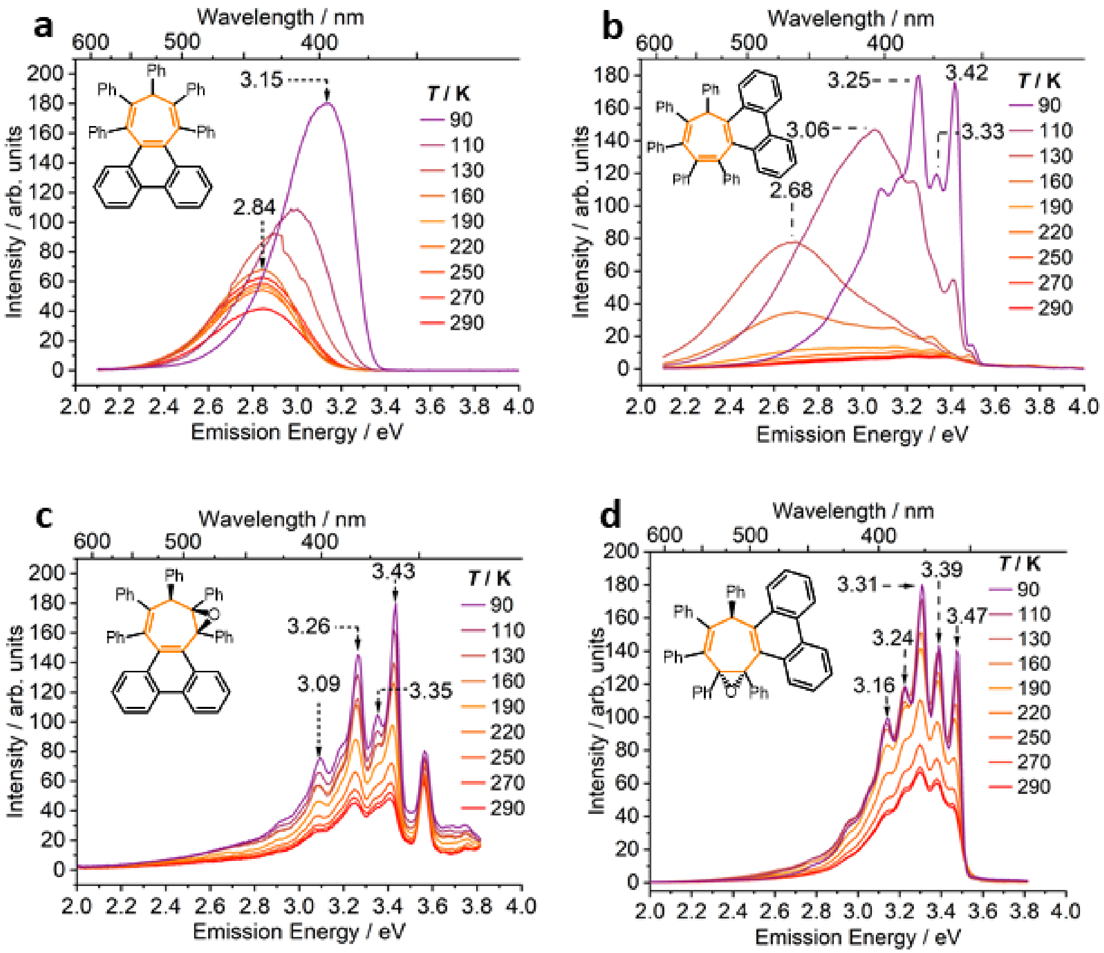

Figure S54. VT fluorescence spectra of 2-MeTHF solutions (*l* = 10 mm, *T* = 90–290 K) of (a) ***sym*-phenPh$_5$C$_7$H** λ_{ex} = 300 nm, *c* = 200 µM; (b) ***asym*-phenPh$_5$C$_7$H** λ_{ex} = 300 nm, *c* = 200 µM; (c) ***sym*-phenPh$_5$C$_7$H-O** λ_{ex} = 315 nm, *c* = 200 nM; (d) ***asym*-phenPh$_5$C$_7$H-O** λ_{ex} = 315 nm, *c* = 200 µM. Inset in panel (a): legend for temperatures in all panels.

6.1. Temperature-dependent Solid-state Photoluminescence

The solid-state photoluminescence of carbocycles **Ph_7C_7H, Ph_7C_7H-O**, ***sym*-phenPh$_5$C$_7$H**, ***asym*-phenPh$_5$C$_7$H**, ***sym*-phenPh$_5$C$_7$H-O** and ***asym*-phenPh$_5$C$_7$H-O**) were measured at a temperature range (290–90 K). The molecules were dispersed as a 1% optically clear ZEONEX® cyclic olefin polymer matrix. The results are shown in Figure S55.

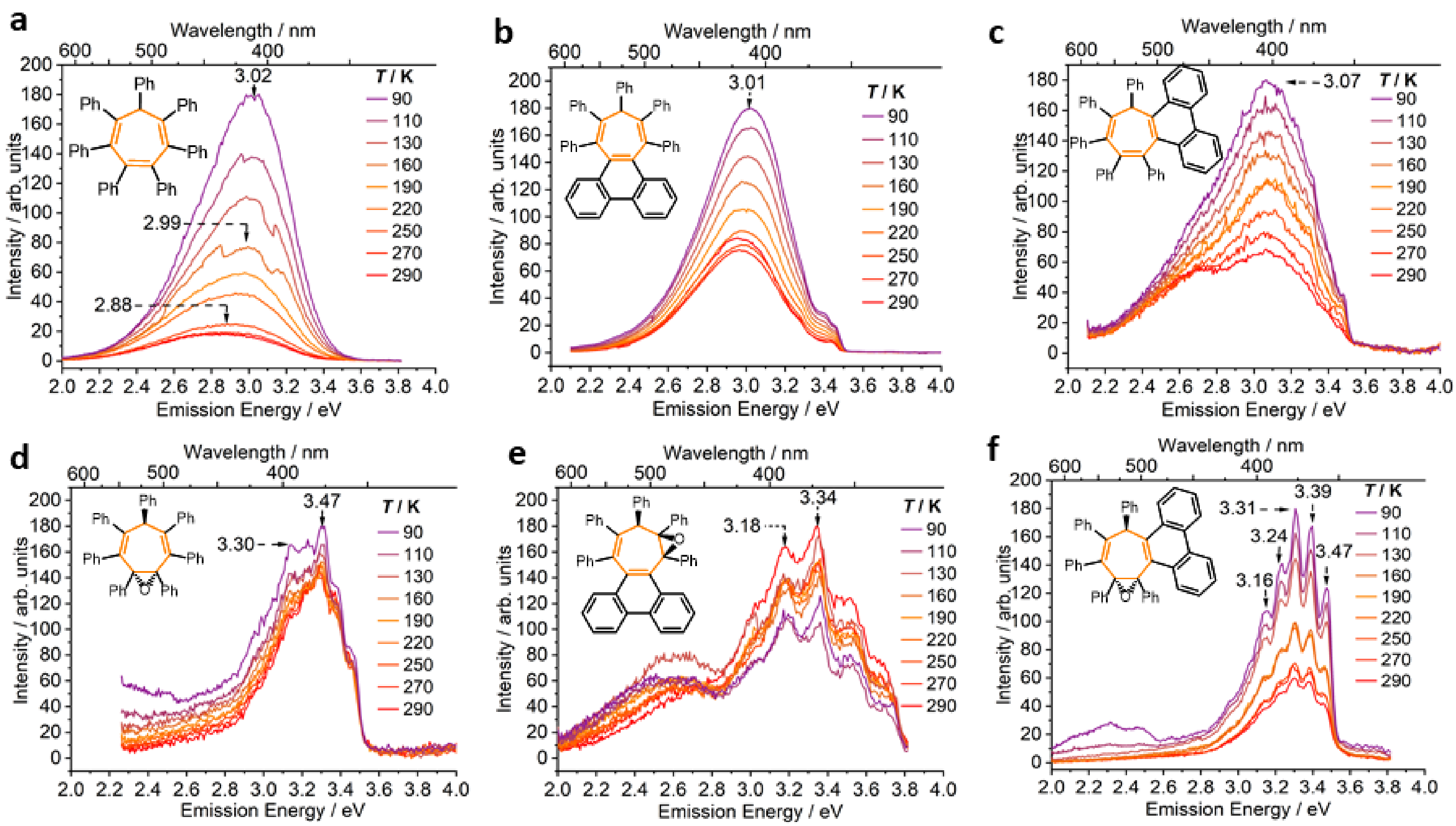

Figure S55. VT fluorescence spectra of ZEONEX matrices containing (a) **Ph_7C_7H** λ_{ex} = 315 nm; (b) **Ph_7C_7H-O** λ_{ex} = 280 nm; (c) ***sym*-phenPh_5C_7H** λ_{ex} = 300 nm; (d) ***asym*-phenPh_5C_7H** λ_{ex} = 315 nm; (e) ***sym*-phenPh_5C_7H-O** λ_{ex} = 315 nm; (f) ***asym*-phenPh_5C_7H-O** λ_{ex} = 315 nm. Inset in panel (a): legend for temperatures in all panels.

7. Fluorescence Solvatochromism

Fluorescence spectra were acquired (Figure S55) for solutions of different polarities. The samples for fluorescence were prepared using 200 μM CH_2Cl_2 stock solutions. The desired quantity was measured into a vial, evaporated to dryness, then the solid residue diluted to 20 μM concentration in the solvent used for the measurements. Although the solvents used cover a large range of polarities, the change in emission wavelength is below 0.1 eV, with the exception of ***asym*-phenPh$_5$C$_7$H** in methylcyclohexane (MCH) where the fluorescence is slightly more red-shifted (presumably as a result of phenanthrene dimer formation in the apolar solvent system). The small magnitude of these differences indicates that the observed emission does not arise from charge transfer states. Such states would be much more influenced by solvent polarity and emission wavelength would vary by a larger amount.

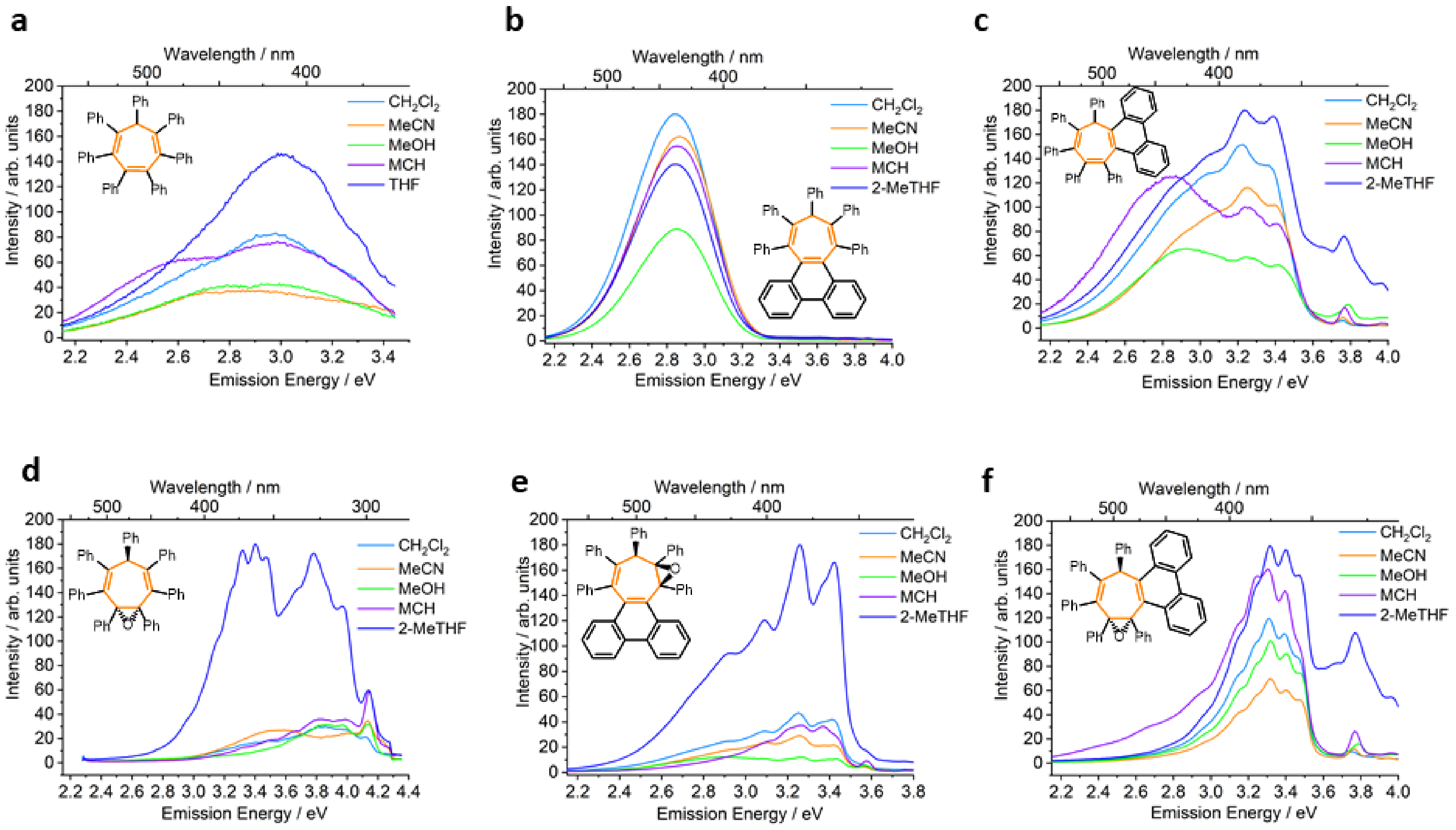

Figure S56. Fluorescence solvatochromism of (a) **Ph_7C_7H** (b) ***sym*-phenPh_5C_7H** (c) ***asym*-phenPh5C7H** (d) **Ph_7C_7H-O,** (e) ***sym*-phenPh_5C_7H-O** (f) ***asym*-phenPh_5C_7H-O** in MCH, CH_2Cl_2, MeOH, MeCN and 2-MeTHF or THF.

8. Phenanthrene

Fluorescence spectra were acquired for molecular phenanthrene. A concentration study (Figure S57a) demonstrated the formation of phenanthrene dimers at concentrations >20 μM showing vibronic emission similar to high concentration samples of ***asym*-phenPh$_5$C$_7$H**. Variable temperature fluorescent spectroscopy of a 1% ZEONEX film (Figure S57b) shows similar vibronic structure and E_{max} to both 2 μM and 20 μM 2-MeTHF samples (Figure S57 c/d) with no observed bathochromic shift as the temperature decreases. This suggests that forming phenanthrene-like units in our molecular rotors via photocyclization, may give rise to vibronic emission from exciting the reversible intermediate form **IM** or oxidized phenanthene containing products. Also, this indicates that the formation of vibronic emission in epoxides is a result of the more favorable formation of phenanthrene moieties.

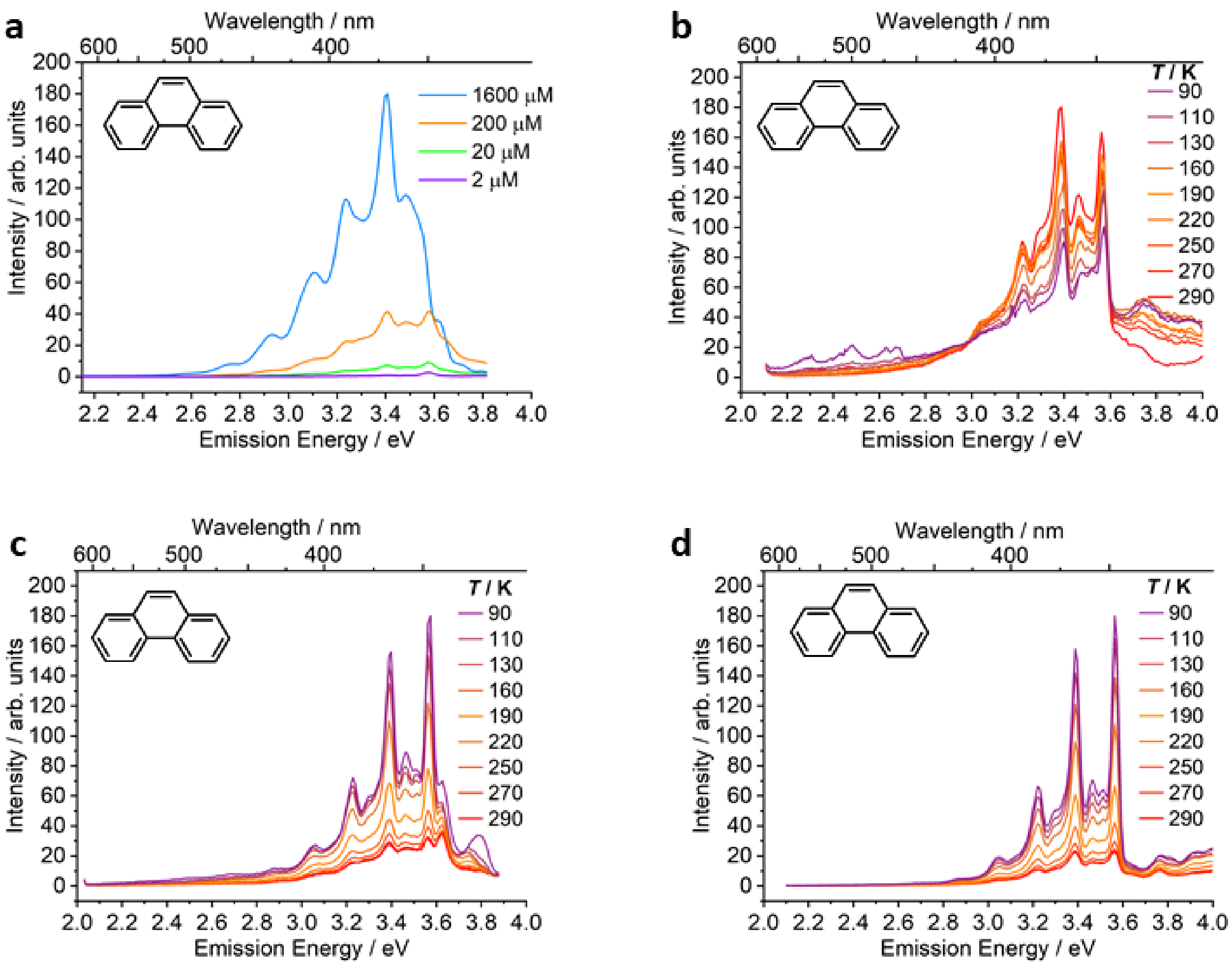

Figure S57. (a) Fluorescence spectra of **phenanthrene** (l = 10 mm, T = 290 K); Insert in panel (a): Legend for concentration. VT fluorescence spectra of phenanthrene in a ZEONEX film (b) **phenanthrene** λ_{ex} = 300 nm. VT fluorescence spectra of phenanthrene in a 2-MeTHF solutions (l = 10 mm, T = 90–290 K) of (c) **phenanthrene** λ_{ex} = 310 nm, c = 20 μM; (d) **phenanthrene** λ_{ex} = 300 nm, c = 20 μM; Inset in panel (b-d): legend for temperatures.

9. Photooxidation

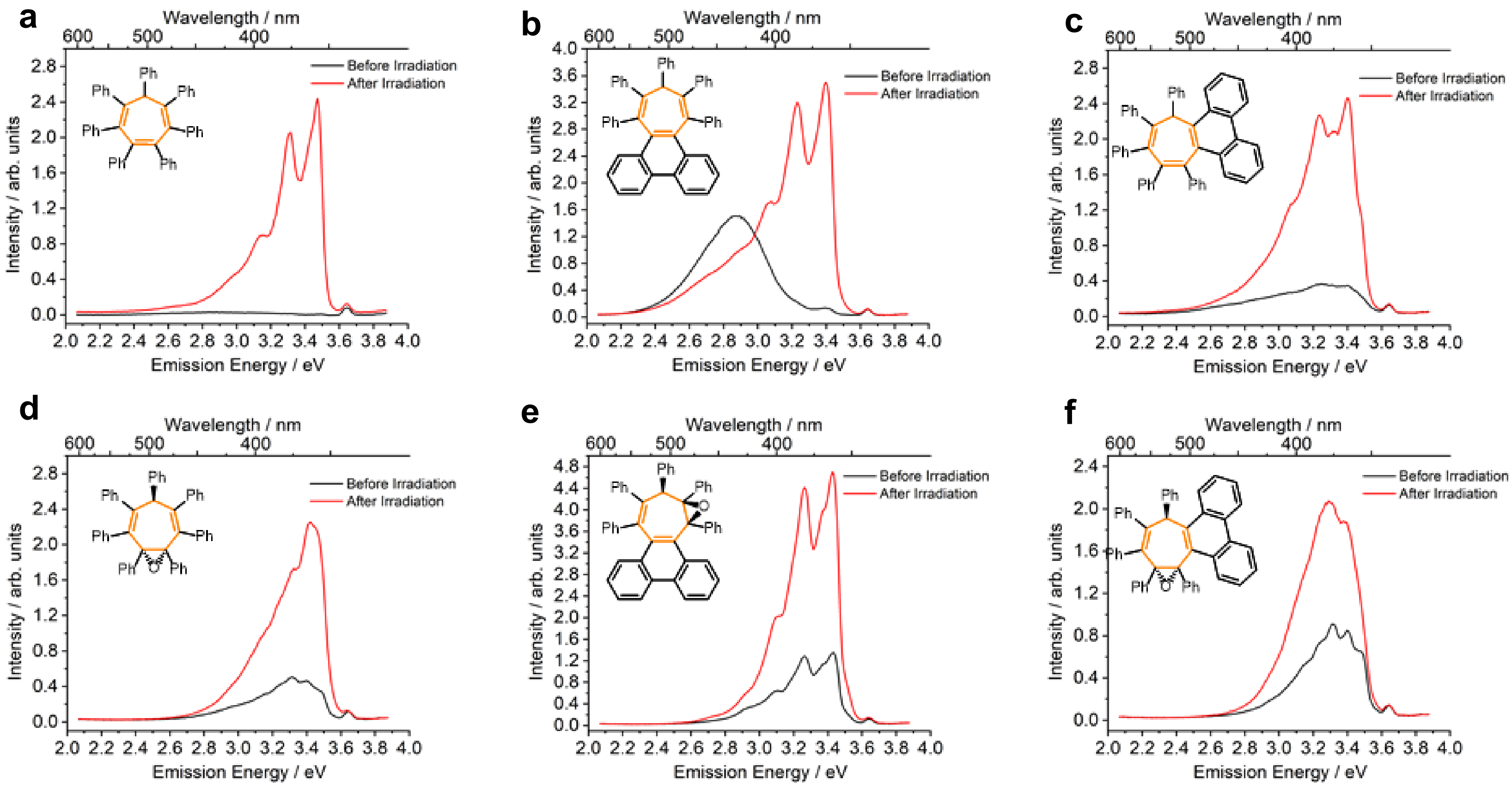

Figure S58. Room temperature emission spectra of 2 μM 2-MeTHF solutions of a) **Ph_7C_7H**, b) ***sym*-phenPh$_5$C$_7$H** c) ***asym*-phenPh$_5$C$_7$H** d) **Ph_7C_7H-O** e) ***sym*-phenPh$_5$C$_7$H-O** f) ***asym*-phenPh$_5$C$_7$H-O,** showing changes in the emission profile before (black) and after (red) prolonged exposure to 4.0 eV light. **a–c** shows the result of >1 h irradiation whereas **d–f** show the result of <5 min irradiation.

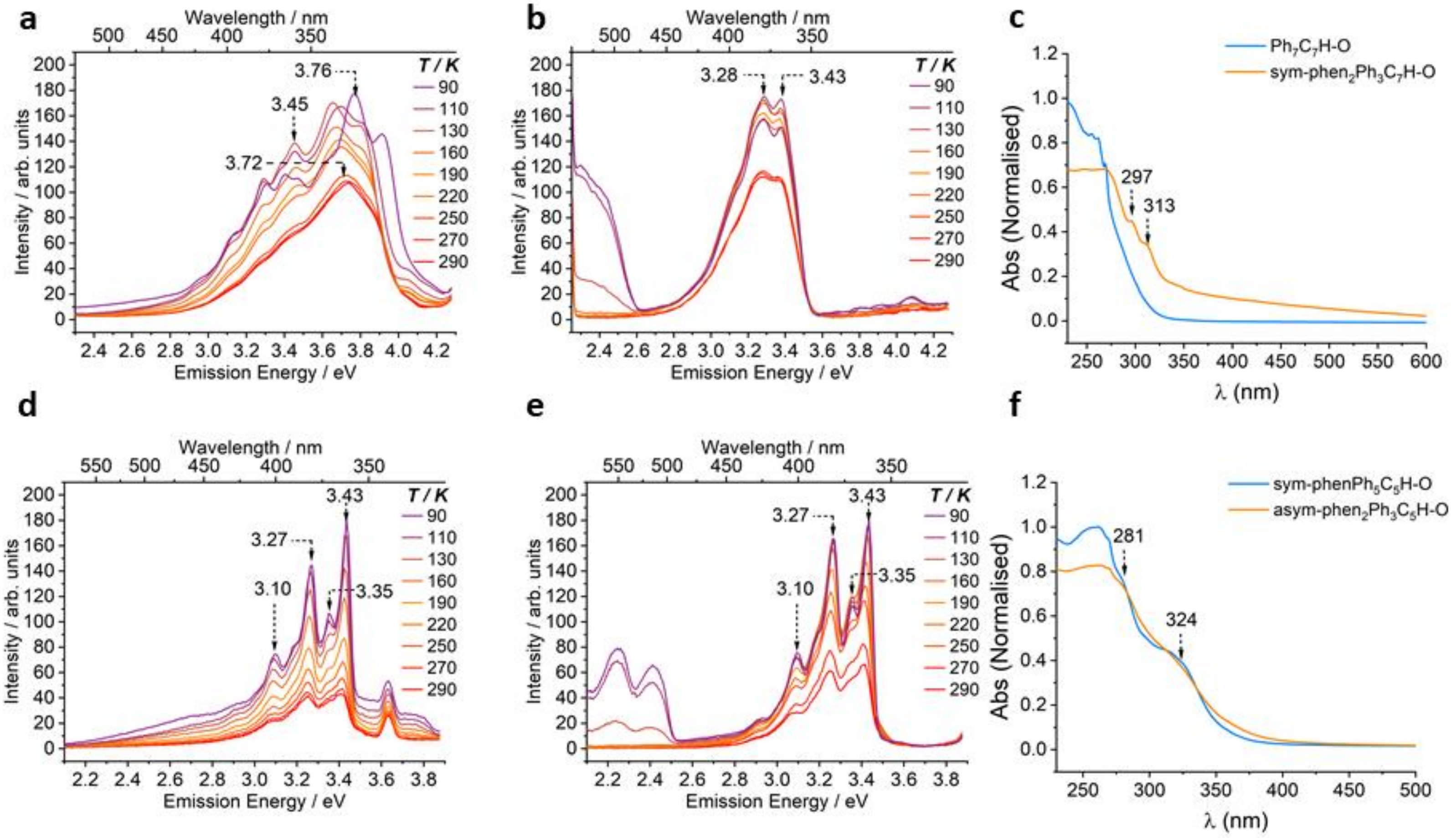

Figure S59. VT fluorescence spectra of photooxidised molecular rotors in 2-MeTHF solutions (*l* = 10 mm, *T* = 90–290 K) of (a) ***sym*-phen$_2$Ph$_3$C$_7$H-O** λ_{ex} = 310 nm, *c* = 20 μM; (d) **a*sym*-phen$_2$Ph$_3$C$_7$H-O** λ_{ex} = 310 nm, *c* = 20 μM; VT fluorescence spectra of photooxidised molecular rotors 1% ZEONEX film of (b) ***sym*-phen$_2$Ph$_3$C$_7$H-O** λ_{ex} = 310 nm, e) **a*sym*-phen$_2$Ph$_3$C$_7$H-O** λ_{ex} = 310 nm; Inset in panel (a,b,d and e): legend for temperatures. UV-absorption spectra in 1% ZEONEX film of c) ***sym*-phen$_2$Ph$_3$C$_7$H-O** f) **a*sym*-phen$_2$Ph$_3$C$_7$H-O**.

10. Time-Correlated Single Photon Counting (TCSPC)

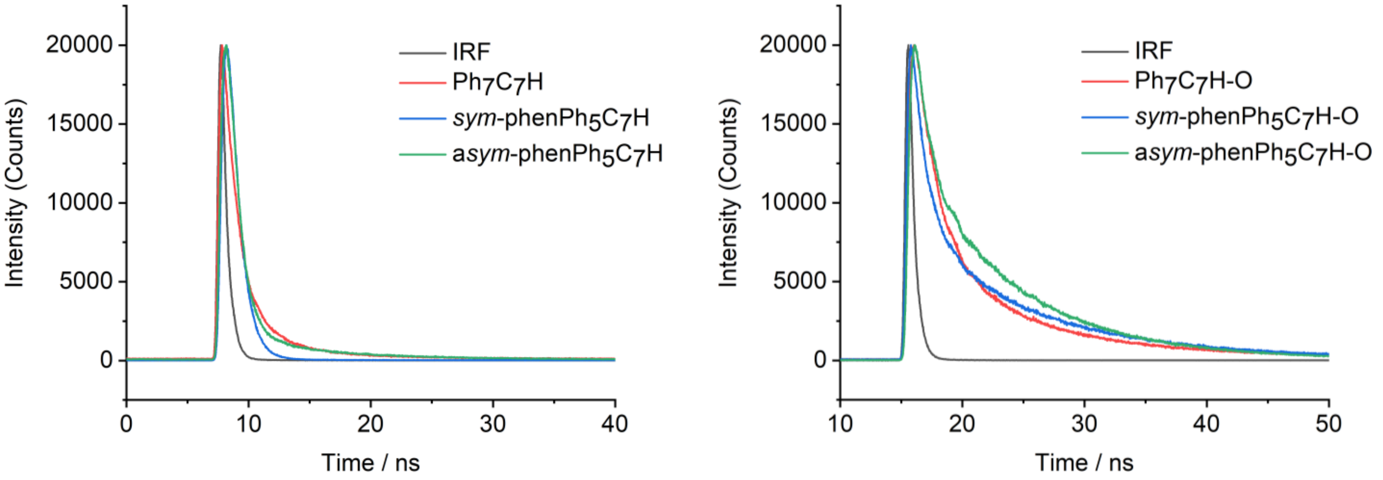

Figure S60. TCSPC decays of molecular rotors in a 2-MeTHF solutions (*l* = 10 mm, *T* = 290 K, *c* = 2 µM) of a) **Ph_7C_7H** λ_{em} = 450 nm, ***sym*-$phenPh_5C_7H$,** λ_{em} = 440 nm and ***asym*-$phenPh_5C_7H$** λ_{em} = 370 nm b) **Ph_7C_7H-O** λ_{em} = 370 nm, ***sym*-$phenPh_5C_7H$-O** λ_{em} = 370 nm and ***asym*-$phenPh_5C_7H$-O** λ_{em} = 370 nm.

Table S4. Lifetimes from the exponential fits for the days of molecular rotors in 2-MeTHF solutions. Assosiated IRF set labeled, after rotor name in each case. IRF data outlined in Table S5. [a]Single exponentional fit y = y0 + A1*exp(-(x-x0)/t1) used.

Rotor	IRF	R^2	y = y0 + A1*exp(-(x-x0)/t1) + A2*exp(-(x-x0)/t2)			
			a1	τ1 /ns	a2	τ2 /ns
C_7Ph_7H-O	A	0.997	0.48233	2.68932	0.20257	12.6032
sym-$phenPh_5C_7H$-O	A	0.997	0.45193	1.98772	0.32792	11.90997
asym-$phenPh_5C_7H$-O	A	0.999	0.22227	1.5013	0.5146	8.96569
C_7Ph_7H	B	0.997	0.65983	1.2868	0.09034	6.53826
sym-$phenPh_5C_7H$	B	0.996	0.85205	0.91697	0.00233	20.43031
asym-$phenPh_5C_7H$	B	0.997	2.96577	0.95146	0.08254	8.16175
sym-$phen_3PhC_7H$[a]	C	0.998	1	8.41089	-	-

Table S5. Lifetimes from the exponential fits for the days of molecular rotors in 2-MeTHF solutions.

IRF	R^2	double z = (x-xc)/w - w/t0; y = y0 + A/t0 * exp(0.5*(w/t0)^2-(x-xc)/t0)*(erf(z/sqrt(2))+1)/2;)				
		y0	A	xC	w	t0
A	0.997	0±0	0.88151	15.36179	0.17497	0.48048
B	0.997	0±0	0.88217	7.52282	0.16955	0.49436
C	0.998	0±0	0.87172	23.23267	0.16619	0.49784

11. New Photocyclized Rotors

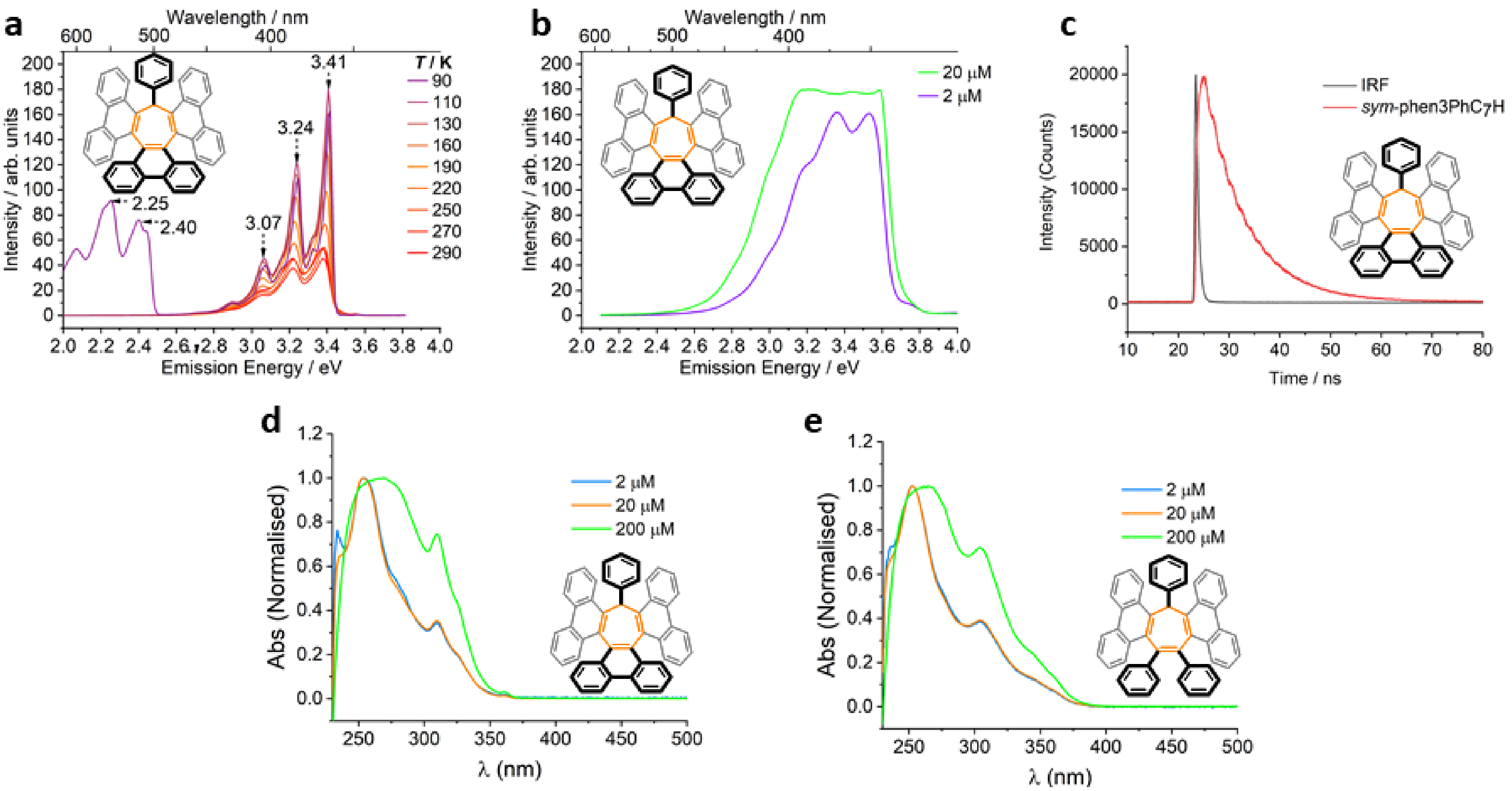

Figure S61. a) VT fluorescence spectra of photooxidised molecular rotors in a 2-MeTHF solutions (*l* = 10 mm, *T* = 90–290 K) of ***sym*-phen$_3$PhC$_7$H** λ_{ex} = 315 nm, *c* = 2 µM; b) Fluorescence spectra of ***sym*-phen$_3$PhC$_7$H** (*l* = 10 mm, *T* = 290 K); Insert in panel (b): Legend for concentration; (c) TCSPC decays of molecular rotors in a 2-MeTHF solutions (*l* = 10 mm, *T* = 290 K, *c* = 2 µM) of a) ***sym*-phen$_3$PhC$_7$H** λ_{em} = 365 nm; UV-Vis absorption spectra of solutions of (d) ***sym*-phen$_3$PhC$_7$H** (e) ***sym*-phen$_2$Ph$_3$C$_7$H** at a series of concentrations in 2-MeTHF.

12. Hyperchromism

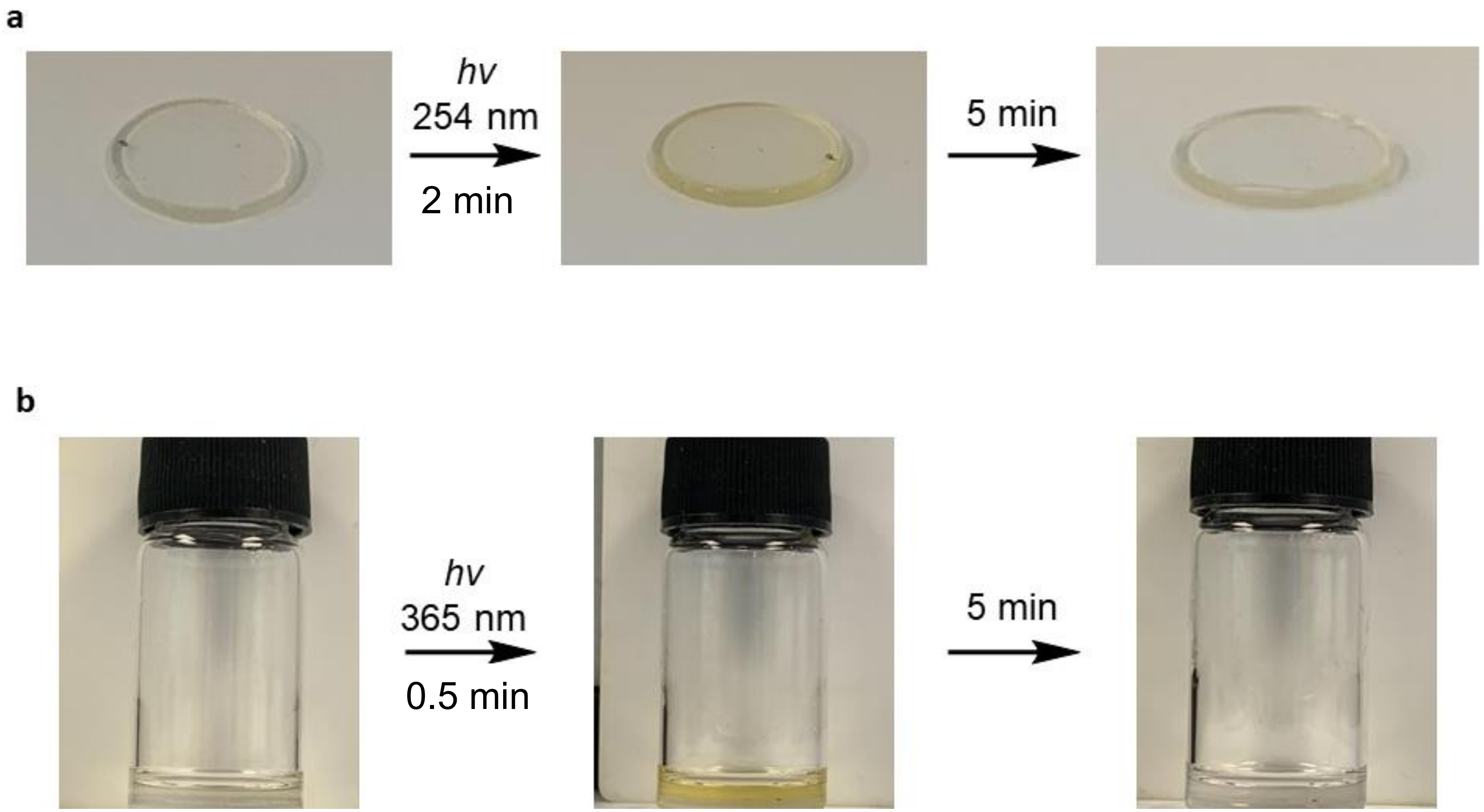

Figure S62. Photographs demonstrating the hyperchromic effect in ***sym*-phenPh$_5$C$_7$H-O** through reversible yellowing upon short irradiation with 365 nm light; *a)* 1% ZEONEX Film; b) 1.5 mmol solution in PhMe.

13. Computational Details

Ground state geometry optimization of ***sym*-phen$_2$Ph$_3$C$_7$H-O** was conducted *in vacuo* employing with SCS-MP2 method (spin-component-scaled Møller-Plesset perturbation theory of second order)[13] along with an SVP basis set. The S_1 excited-state geometry optimization was performed with SCS-ADC(2) method (spin-component-scaled adiabatic diagrammatic construction up to second order)[14] with an SVP basis set. All electronic transitions were calculated with SCS-ADC(2) using a TZVP basis set, on the S_0 or S_1 geometries obtained with SCS-MP2/SVP or SCS-ADC(2)/SVP. All calculations were performed with the Turbomole 7.4.1 program package.[15]

To further investigate the shape of the S_1 emission band, we included non-Condon effects in our calculations by using the nuclear ensemble approach.[16] We optimized the S_1 geometry of ***sym*-phen$_2$Ph$_3$C$_7$H-O** using linear-response time-dependent density functional theory (LR-TDDFT) within the Tamm-Dancoff approximation, the ωB97X-D functional, and a 6-31G* basis set. The minimum-energy geometry obtained on S_1 shows a similar electronic character as the one calculated with SCS-ADC(2). At the LR-TDDFT/TDA S_1 geometry, LR-TDDFT/TDA/ωB97X-D/6-31G* gives a vertical S_1/S_0 energy of 3.98 eV, while SCS-ADC(2)/TZVP (on the same geometry) indicates a transition energy of 3.64 eV. Frequencies were obtained at the same level of theory and used to calculate an approximated Wigner distribution for the lowest vibrational states of the S_1 electronic state. 100 geometries were sampled from this Wigner distribution. For each of these geometries, S_1/S_0 emission energy and oscillator strength were calculated at the LR-TDDFT/TDA/ωB97X-D/6-31G* level of theory. An emission spectrum accounting for non-Condon effects is then obtained by grouping all the transitions, each broadened by a Gaussian with a width of 0.1 eV. The obtained emission spectrum (Figure S63) reproduces the width of the

experimental one. The NEA spectrum was obtained with Newton-X 2.0[17] interfaced with Gaussian 09 revision D.01 for the electronic structure.[18]

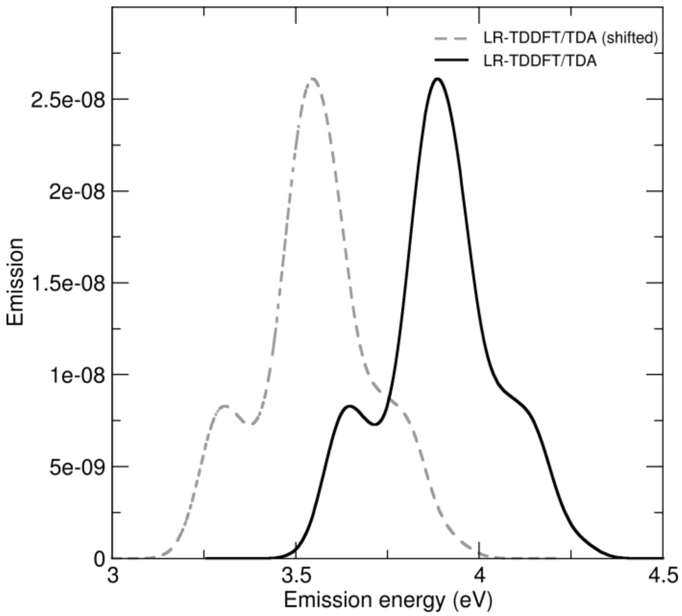

Figure S63. Emission spectrum calculated with the NEA using LR-TDDFT/TDA/ωB97X-D/6-31G* (see text for details). The shifted spectrum corresponds to the original LR-TDDFT/TDA spectrum shifted by the energy difference (0.34 eV) between the transition calculated with SCS-ADC(2)/TZVP and LR-TDDFT/TDA/ωB97X-D/6-31G* at the S_1 optimized geometry (obtained with LR-TDDFT/TDA/ωB97X-D/6-31G*).

14. References

1 O. V. Dolomanov, L. J. Bourhis, R. J. Gildea, J. A. K. Howard and H. Puschmann, *J. Appl. Crystallogr.*, **2009**, 42, 339–341.

2 G. M. Sheldrick, *Acta Crystallogr. Sect. A Found. Crystallogr.*, **2008**, 64, 112–122.

3 J. Sturala, M. K. Etherington, A. N. Bismillah, H. F. Higginbotham, W. Trewby, J. A. Aguilar, E. H. C. Bromley, A.-J. Avestro, A. P. Monkman and P. R. McGonigal, *J. Am. Chem. Soc.*, **2017**, 139, 17882–17889.

4 H. J. Reich, *J. Chem. Educ.*, **1995**, 72, 1086.

5 J. Da Chai and M. Head-Gordon, *Phys. Chem. Chem. Phys.*, **2008**, 10, 6615–6620.

6 W. J. Hehre, K. Ditchfield and J. A. Pople, *J. Chem. Phys.*, **1972**, 56, 2257–2261.

7 P. C. Hariharan and J. A. Pople, *Theor. Chim. Acta*. **1973**, 28, 213–222.

8 A. Dreuw and M. Wormit, *Wiley Interdiscip. Rev. Comput. Mol. Sci.* **2015**, 5, 82–95.

9 A. Schäfer, H. Horn and R. Ahlrichs, *J. Chem. Phys.* **1992**, 97, 2571–2577.

10 T. H. Dunning, *J. Chem. Phys.* **1989**, 90, 1007–1023.

11 F. Furche, R. Ahlrichs, C. Hättig, W. Klopper, M. Sierka and F. Weigend, *Wiley Interdiscip. Rev. Comput. Mol. Sci.* **2014**, 4, 91–100.

12 W. Humphrey, A. Dalke and K. Schulten, *J. Mol. Graph.* **1996**, 14, 33–38.

13 S. Grimme, L. Goerigk and R. F. Fink. *Wiley Interdiscip. Rev. Comput. Mol. Sci.*, **2012**, 2, 886-906.

14 A. Dreuw and M. Wormit. *Wiley Interdiscip. Rev. Comput. Mol. Sci*. **2015**, 5, 82-95.

15 F. Furche, R. Ahlrichs, C. Hättig, W. Klopper, M. Sierka and F. Weigend. *Wiley Interdiscip. Rev. Comput. Mol. Sci*, **2013**, 4, 91-100.

16 Crespo-Otero, R.; Barbatti, M. *Theor. Chem. Acc*. **2012**, 131, 1237.

17 Barbatti, M.; Ruckenbauer, M.; Plasser, F.; Pittner, J.; Granucci, G.; Persico, M.; Lischka, H. *Wiley Interdiscip. Rev. Comput. Mol. Sci*. **2014**, 4, 26−33.

18 Frisch, M. J. et al. Gaussian 09, Revision D.01; Gaussian Inc.: Wallingford CT, **2013**.
